# Measurement of the p*K*_a_ Values of Organic Molecules in Aqueous–Organic Solvent Mixtures by ^1^H NMR without External Calibrants

**DOI:** 10.1021/acs.analchem.3c02771

**Published:** 2023-10-13

**Authors:** Matthew Wallace, Nduchi Abiama, Miranda Chipembere

**Affiliations:** School of Pharmacy, University of East Anglia, Norwich Research Park, Norwich NR4 7TJ, U.K.

## Abstract

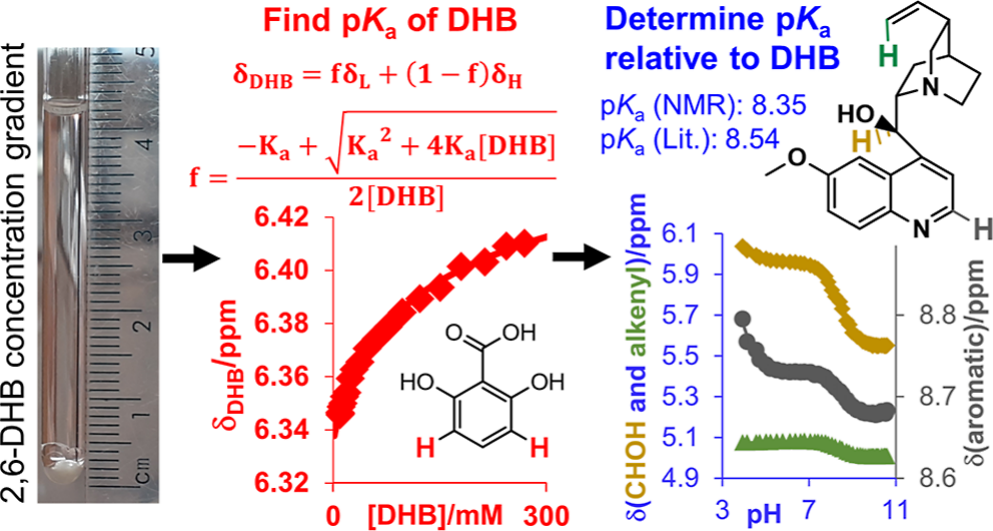

Aqueous–organic solvent mixtures are commonly used for reactions or analyses, where the components of a system are insoluble in pure water. The acid dissociation constant is an important property to measure in these media as it determines the charge state, solubility, and reactivity of a molecule. While NMR spectroscopy is an established tool for the measurement of p*K*_a_ in water, its use in aqueous–organic solvents is greatly hindered by the requirement for external calibrants on which a working pH scale is set. Such calibrants include buffer solutions, “anchor” molecules with known p*K*_a_ values, and pH electrodes that have undergone lengthy calibration procedures in the solvent mixture of interest. However, such calibrations are often inconvenient to perform, while literature p*K*_a_ data covering the required range may not be available at the solvent composition or the temperature of interest. Here, we present a method to determine p*K*_a_ in aqueous–organic solvents directly by NMR. We first determine p*K*_a_ of an organic acid such as 2,6-dihydroxybenzoic acid (2,6-DHB) by measuring its ^1^H chemical shift as a function of concentration along a concentration gradient using chemical shift imaging (CSI). Using 2,6-DHB as a reference, we then determine p*K*_a_ of less acidic molecules in single CSI experiments via the variation of their ^1^H chemical shifts along pH gradients. As proof of concept, we determine the p*K*_a_ values of organic acids and bases up to p*K*_a_ 10 in 50% (v/v) 1-propanol/water, 50% (v/v) dimethyl sulfoxide/water, and 30% (v/v) acetonitrile/water and obtain good agreement with the literature values.

## Introduction

Mixtures of water and organic solvents are commonly used as media for analytical procedures,^1^ synthetic reactions,^2^ and work with biological tissues.^3^ The properties of these mixtures differ from those of the pure solvents, allowing modulation of the polarity, viscosity, and freezing point via the solvent composition.^3^ These properties in turn modulate the solubility,^4^ acidity/basicity,^5^ and reactivity^2^ of the dissolved molecules. The acid dissociation constant of an organic molecule (*K*_a_, normally expressed as the negative logarithm, p*K*_a_) is very sensitive to the solvent composition and can be changed by more than one log unit relative to its aqueous value by inclusion of 50 wt % of an organic solvent.^6–8^ Literature data are only available in a small number of solvent mixtures, while extrapolations of p*K*_a_ between different solvent mixtures are only possible within narrow classes of compounds and require reliable reference data.^9,10^ For these reasons, it is often necessary to measure the p*K*_a_ values of organic molecules experimentally when working in aqueous–organic solvents.

The electrochemical measurement of pH requires careful calibration of electrodes^5^ either using solutions of known pH in the solvent mixture of interest^11–14^ or by applying a specific correction to the pH reported by an electrode that has been calibrated using conventional aqueous buffers.^5,15^ Alternatively, pH may be determined from the NMR chemical shifts or UV/vis spectra of compounds with known p*K*_a_ values.^8,16^ However, electrode corrections and p*K*_a_ data are only available in a small number of aqueous–organic mixtures.^5,15^ Finally, p*K*_a_ values can be measured without knowledge of pH using conductometric methods.^17^ However, such approaches require high-purity materials, and only one compound may be analyzed per titration.

**Scheme 1:**
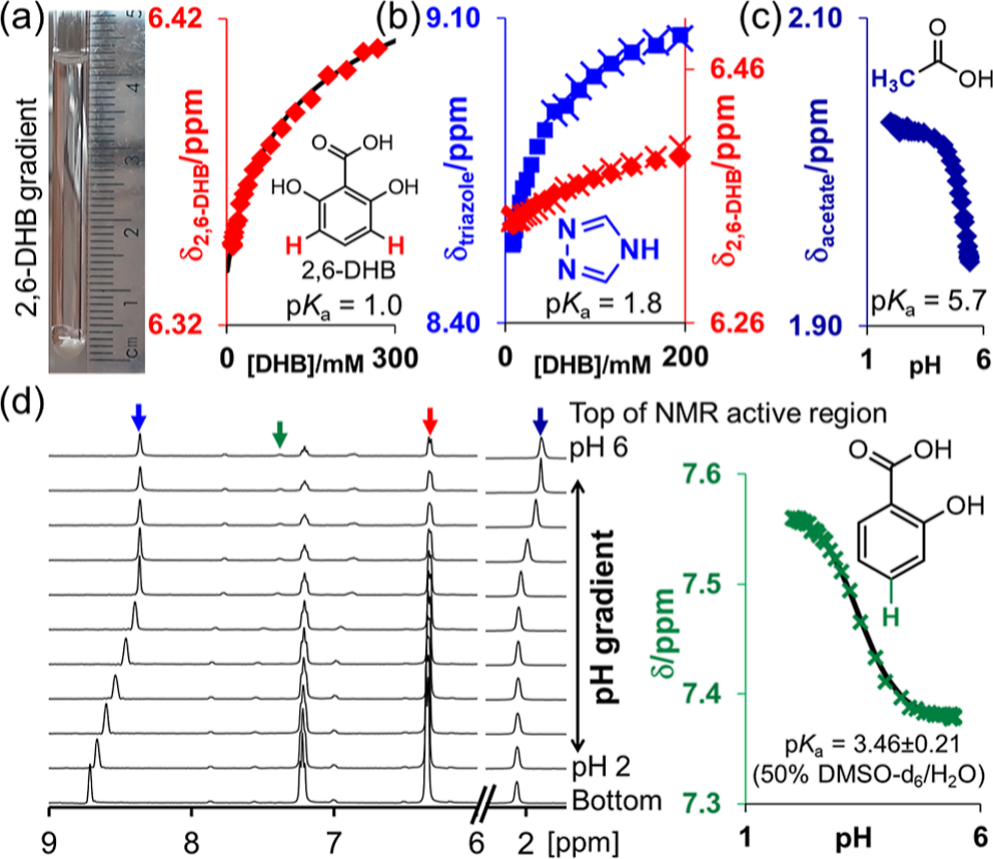
Method to Determine p*K*_a_ of Organic Molecules in Aqueous–Organic Mixtures Using CSI and Concentration Gradients of 2,6-DHB The ^1^H chemical shift of 2,6-DHB is measured as a function of concentration in the absence of base (a) and the presence of 40 mM 1,2,4-triazole (b) to determine the p*K*_a_ of both compounds. The p*K*_a_ values of other indicator molecules are determined relative to triazole and 2,6-DHB (c) allowing the determination of pH from their ^1^H chemical shifts and p*K*_a_ of other organic molecules using pH gradients and CSI (d).

Here, we demonstrate how p*K*_a_ values can be determined directly by ^1^H chemical shift imaging (CSI) NMR in mixtures of compounds, without the use of electrochemical measurements or literature p*K*_a_ data. In our method, a concentration gradient of 2,6-dihydroxybenzoic acid (2,6-DHB) is established in an NMR tube by placing solid acid at the base of the tube and layering the solvent mixture of interest on top. Spatially resolved ^1^H spectra are recorded at different positions along the gradient using CSI and a p*K*_a_ value extracted in a single experiment by analysis of how the ^1^H chemical shift of 2,6-DHB varies with concentration. The experiment is then repeated in the presence of 1,2,4-triazole and a p*K*_a_ of this compound is determined, and the p*K*_a_ of 2,6-DHB is verified by considering the degree of proton transfer from 2,6-DHB to triazole. The p*K*_a_ values of organic molecules spanning p*K*_a_ 3–10 are then determined relative to 1,2,4-triazole and 2,6-DHB, allowing the determination of pH between 1 and 12 from their ^1^H chemical shifts. We can, thus, establish a working set of indicator molecules to determine pH in an aqueous–organic mixture by running four ^1^H CSI experiments (Scheme 1). These indicators allow the p*K*_a_ of organic molecules to be determined in single 20 min CSI experiments from the variation of their ^1^H chemical shift along the pH gradients.^16,18^

All experiments can be run under full automation on standard high-field NMR equipment, allowing for a convenient calibration of the indicators at the solvent composition and the temperature of interest. As proof of concept, we determine the p*K*_a_ of a range of acids and bases in 50% (v/v) 1-propanol/water, 50% (v/v) dimethyl sulfoxide-*d*_6_ (DMSO)/water, and 30% (v/v) acetonitrile-*d*_3_ (CD_3_CN)/water at 298 K. The uncertainties in the fitted p*K*_a_ values are less than 0.5 units in most cases, while agreement with literature data is obtained within these uncertainties. While NMR is an established tool to determine the relative p*K*_a_ values of organic molecules,^19^ to the best of our knowledge, the direct measurement of absolute p*K*_a_ by NMR has not previously been described. All processing can be performed using the automation routines provided in Section S10 of the Supporting Information and the spreadsheet accompanying this work.

## Experimental Section

### Materials

All reagents were purchased from commercial suppliers and used as received. Phthalic acid was used as the monopotassium salt; otherwise, Na^+^ was the counterion in all experiments. Milli-Q water (18.2 MΩ cm) was used throughout the study. The DMSO-*d*_6_ and CD_3_CN had deuteration levels of 99.8 and 99%, respectively. The 50% (v/v) 1-propanol/H_2_O mixture was prepared by combining D_2_O (10 mL), H_2_O (40 mL), and 1-propanol (50 mL).

### Preparation of Samples

Solid 2,6-DHB was transferred to the base of 5 mm NMR tubes (Wilmad 528-PP) by pushing the tip of a 9 in. Pasteur pipet into the solid acid and emptying the tip at the base of the NMR tube. Four, 2 mm diameter glass beads were placed on top of the 2,6-DHB. Prior to use, the beads were rinsed with ethanol and dried. A solution containing indicators, organic analyte molecules, and DSS was layered slowly (10 s) on top of the beads up to a height of 40–45 mm from the base of the NMR tube. The sample was stood in the autosampler rack of the spectrometer (22 °C) prior to analysis. Samples for the determination of the p*K*_a_ of 2,6-DHB and 1,2,4-triazole contained 10 mM DSS to act as a reference for the chemical shift and integration. Other experiments in 50% DMSO/H_2_O and 30% CD_3_CN/H_2_O were performed with 0.4 mM DSS, where only referencing of the chemical shift was required. All experiments in 50% 1-propanol/H_2_O were performed with 10 mM DSS due to the strong background solvent signal. No 2,6-DHB was included as an indicator, with the ^1^H signal arising from the acid diffusing up the NMR tube. Analytes were included as neutral bases or as sodium salts.

**Figure 1:**
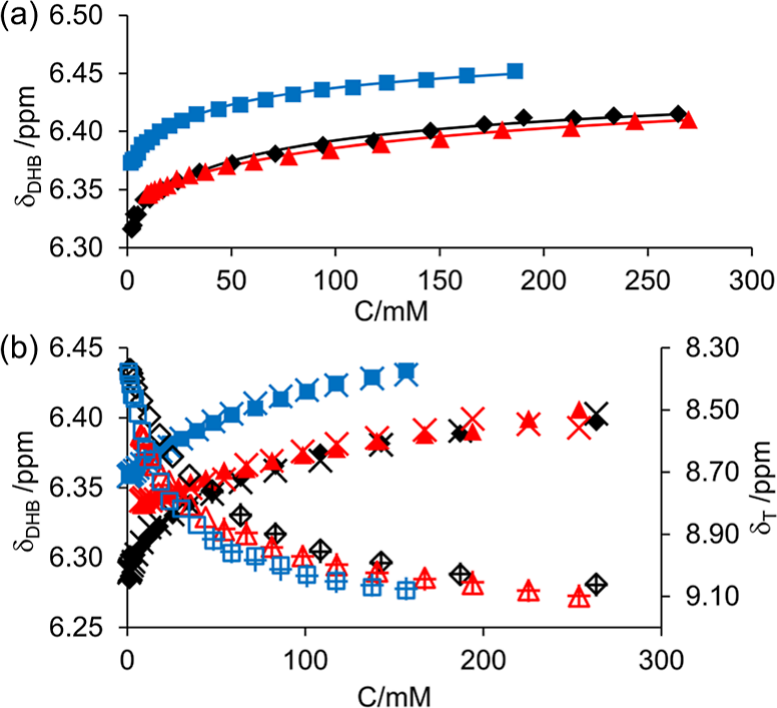
Plot of ^1^H chemical shift of 2,6-DHB (solid symbols) vs *C* in the absence (a) and presence (b) of 40 mM 1,2,4-triazole in 50% 1-propanol/H_2_O (black diamond), 50% DMSO/H_2_O (red triangle), and 30% CD_3_CN/H_2_O (blue square). Solid lines are fits to eqs 1–4. ^1^H chemical shift of 1,2,4-triazole (open symbols). Fits to eq 7 (vertical cross) and eqs 2 and 8 (diagonal cross).

The time, *t* (hours), at which the gradient will have developed was estimated based on the viscosity, η (mPa s), of the solvent mixture as *t* = αη(*Z*_0_^2^ – *Z*_b_^2^)/ln(*C*_b_/*C*_0_). *Z*_b_ is the height of the bottom of the NMR-active region, and *Z*_0_ is the height of the midpoint of our sample above the top of the 2,6-DHB (9 and 19 mm in our instrument, respectively, with the top of the acid lying approximately 1–2 mm above the absolute base of the NMR tube—see the photograph of freshly prepared sample on Scheme 1a). *C*_b_ and *C*_0_ are the concentrations at these positions. α is calculated as 0.1 h mPa^–1^ s^–1^ mm^–2^ at 22 °C based on a self-diffusion coefficient of 2,6-DHB in 50% 1-propanol/H_2_O of 2.8 × 10^–10^ m^2^ s^–1^ at 298 K (Section S2, Supporting Information). Acceptable gradients are obtained at times calculated with the ratio *C*_b_/*C*_0_ between 400 and 6, giving theoretical concentrations (*C*_top_) less than 2 mM at the top of the NMR active region (*Z*_top_, 29 mm above 2,6-DHB in our instrument). The time windows are, thus, 12–41, 14–47, and 4–13 h for 50% 1-propanol/H_2_O, 50% DMSO/H_2_O, and 30% CD_3_CN/H_2_O, respectively. The p*K*_a,0_ values determined over these time windows agree within uncertainties (Section S2). For determination of the p*K*_a,0_ of imidazole (IM) in 50% 1-propanol/water, where an excess of a much stronger base was present [20 mM of dimethylglycine (DMG) Na salt] and only 32 points were collected in the CSI data set, a more gentle pH gradient was employed where appreciable acid was present at *Z*_top_ (*C*_b_/*C*_top_ = 20, *t* = 66 h). The mass, m, of 2,6-DHB is calculated based on the desired *C*_b_ as , where *r* is the tube radius (2.1 mm), *M*_r_ is the molecular mass of 2,6-DHB (154.12 g/mol), and *D* is the diffusion coefficient of 2,6-DHB. For determination of the p*K*_a,0_ of 2,6-DHB and triazole (Figure 1), 8–9 mg of 2,6-DHB was used, giving *C*_b_ between approximately 120 and 200 mM over the time window. A total of 4–5 mg was used for calibration of the other indicators (Table 1) and to determine the p*K*_a_ of the organic analytes (Table 2), unless otherwise stated, giving *C*_b_ between 60 and 100 mM. *C*_b_ is chosen to provide an excess of 2,6-DHB over the base at *Z*_b_.

**Table 1:** p*K*_a,0_, δ_H_, and δ_L_ for NMR Indicators

50% 1-propanol/H_2_O				50% DMSO/H_2_O			30% CD_3_CN/H_2_O		
indicator	p*K*_a,0_	δ_H_/ppm	δ_L_/ppm	p*K*_a,0_	δ_H_/ppm	δ_L_/ppm	p*K*_a,0_	δ_H_/ppm	δ_L_/ppm
2,6-DHB^a^	1.61 ± 0.29	6.4781	6.3113	1.02 ± 0.19	6.4911	6.3362	1.54 ± 0.40	6.4999	6.3680
1,2,4-triazole	1.80 ± 0.36	9.2221	8.3164^b^	1.77 ± 0.19	9.1934	8.3621^b^	2.33 ± 0.41	9.1539	8.3278^b^
DMG^c^				2.64 ± 0.21	4.0328	3.5755	2.45 ± 0.42	3.9985	3.6370
salicylic acid				3.33 ± 0.22	7.5679	7.3772			
glycolic acid	4.47 ± 0.36	4.1722	3.9171^d^	4.82 ± 0.30	4.0994	3.7583^d^	4.43 ± 0.46	4.1716	3.8643^d^
acetic acid	5.47 ± 0.38	2.0516	1.9160^d^	5.72 ± 0.31	2.0293	1.8048^d^	5.46 ± 0.54	2.0583	1.8485^d^
IM	5.93 ± 0.44	8.7791	7.7155				6.61 ± 0.56	8.6442	7.7254
2MI	6.87 ± 0.47	2.6227	2.3574	6.66 ± 0.35	2.5790	2.3181	7.56 ± 0.59	2.5717	2.3233
4CN	8.54 ± 0.48	7.5160	7.2840	8.29 ± 0.37	7.6617	7.3510	8.44 ± 0.61	7.6370	7.4052
DMG	9.50 ± 0.50	2.9373^e^	2.2328^e^	9.45 ± 0.47	3.5870^c^	2.8685^c^	9.83 ± 0.69	3.6512^c^	2.9289^c^

a p*K*_a,0_, δ_L_, and δ_H_ obtained in the absence of 1,2,4-triazole using eqs 1–4, uncertainty obtained from experiment with 40 mM 1,2,4-triazole.

b Average of δ_L_ determined in solution of triazole (40 mM) and DSS and in acidic range sample.

c CH_2_ resonance of DMG.

d Average of acidic and basic range samples in the absence of 2,6-DHB.

e Methyl resonance of DMG.

**Table 2:** Comparison of p*K*_a,0_ of Analyte Molecules Determined by ^1^H CSI with Literature Values

50%1-propanol/H_2_O				50% DMSO/H_2_O				30% CD_3_CN/H_2_O			
analyte	indicator	p*K*_a,0 NMR_	p*K*_a,0Lit_	analyte	indicator	p*K*_a,0 NMR_	p*K*_a,0 Lit_	analyte	indicator	p*K*_a,0 NMR_	p*K*_a,0Lit_
salicylic acid^a^	2,6-DHB, triazole, glycolate, acetate	4.12 ± 0.37	4.17^17^	salicylic acid^a^	2,6-DHB, triazole, DMG, glycolate, acetate	3.46 ± 0.21	3.48^8^	salicylic acid^a^	2,6-DHB, triazole, DMG, glycolate, acetate	3.69 ± 0.42	3.43,^27^ 3.74,^28^ 3.93^29^
benzoic acid^b^	2,6-DHB, triazole, glycolate, acetate, 2MI	5.52 ± 0.38	5.50^7^	benzoic acid	triazole, glycolate, acetate, 2MI	5.25 ± 0.31	5.23,^8^ 5.73^30^	benzoic acid	triazole, glycolate, acetate, 2MI	5.10 ± 0.49	4.78,^31^ 5.10,^29^ 5.23^30^
picolinic acid^b^	2,6-DHB, triazole, glycolate, acetate, 2MI	1.85^c^, 5.29 ± 0.38	2.16,^32^ 5.52^32^	Bes^d^	triazole, glycolate, acetate, 2MI	6.72 ± 0.34	7.03,^12^ 7.18^33^	phthalic acid	triazole, glycolate, acetate, 2MI	3.48 ± 0.43, 6.07 ± 0.51	3.41,^6^ 3.46,^1^ 6.53^1^
acetylacetone	IM, 2MI, DMG	9.23 ± 0.50^e^	9.71^34^	4CN^f^	2MI, DMG	8.25 ± 0.45	8.56^35^	quinine^g^	DMG, glycolate, acetate, IM, 2MI, DMG	3.55^c^, 8.35 ± 0.65	8.54^36^
pipecolic acid	triazole, 2MI, DMG	2.33^c^, 10.34 ± 0.50	2.87,^32^ 10.47^32^	d-valine^f^	2MI, DMG	3.29^c^, 9.29 ± 0.47	9.67^37^	benzylamine^h^	DMG, 2MI, DMG	8.89 ± 0.69	9.26^36^

a Acidic-range data set.

b 8–9 mg of 2,6-DHB.

c Approximate p*K*_a1_ from fitting to eq 14.

d Sample also contained DMG sodium salt (2 mM), tricine (2 mM), formate (4 mM), and *tert*-butylamine (10 mM), which were found unsuitable for use as indicators. A total of 5–6 mg of 2,6-DHB.

e Value corrected for enol-ketone tautomerization.

f Sample also contained NaOH (10 mM), d-valine Na salt (2 mM), and 4CN sodium salt (20 mM).

g Basic-range data set.

h Sample contained NaOH (10 mM) in addition to indicators. A total of 3–4 mg of 2,6-DHB.

### NMR

All experiments were performed at 298 K on a Bruker 500 MHz Avance III spectrometer, locking and shimming to D_2_O, DMSO-*d*_6_, or CD_3_CN. ^1^H chemical shift images were acquired using a gradient phase encoding sequence based on that of Trigo-Mouriño et al.^20^ For 50% 1-propanol/H_2_O, a spin-echo sequence (π/2–τ–π–gp acquire) was used, where gp is a magnetic field gradient pulse of 166 μs duration that was ramped from −8 to 8 G/cm in 32 steps. τ is a delay to balance the gradient pulse and gradient recovery delay (200 μs). π/2 was 10 μs. Eight scans were acquired at each gradient increment with a signal acquisition time and relaxation delay of 2.15 and 3.0 s, respectively, and a sweep width of 20 ppm. A spoil gradient pulse (600 μs, 25 G/cm) and recovery delay (200 μs) were executed before the π/2 pulse. The spoil and phase encoding gradient pulses were in the form of smoothed squares. Sixteen dummy scans were performed prior to signal acquisition, giving a total experimental time of 23 min 30 s. The vertical range of the CSI experiment (cnst0, Section S11) was set to 3.2 cm, giving a theoretical resolution of 1.0 mm.

Experiments in 50% DMSO/H_2_O and 30% CD_3_CN/H_2_O were performed with suppression of the H_2_O resonance using a CSI sequence incorporating excitation sculpting with perfect-echo^21^ (Bruker library, zgesgppe) and 4 ms Gaussian inversion pulses. To determine the p*K*_a,0_ of 2,6-DHB and 1,2,4-triazole, CSI data sets were collected with the same gradient, acquisition time, and relaxation delay described above for experiments in 50% 1-propanol/H_2_O. To determine the p*K*_a_ values of other compounds in these solvents and acetylacetone in 50% 1-propanol/H_2_O, the encoding gradient pulse was ramped from −16.5 to 16.5 G/cm in 64 steps with a signal acquisition time and relaxation delay of 1.17 and 1.0 s giving a total experimental time of 19 min 38 s. The vertical range of the CSI experiment was set to 3.0 cm.

### Data Processing

^1^H spectra and CSI data sets were processed with an exponential line broadening factor of 3 Hz and 64 K or 32 K points, respectively. CSI data sets were processed in phase-sensitive mode, with phase, baseline correction, and referencing to DSS (0 ppm) performed automatically using the scripts and routines described in Section S10, Supporting Information. Only rows 7–27 (32-point CSI data set) or 14–57 (64-point data set), covering the region approximately 11–31 mm from the base of the NMR tube, were used for analysis to avoid artifacts relating to off coil effects. Calculations are performed by the spreadsheet accompanying this work. The concentration of 2,6-DHB was determined from the integrals, h, of 2,6-DHB (3,5-position of aromatic ring) and DSS (methyl) as *C* = *kh*_2,6-DHB_/*h*_DSS_, where *k* = 44.0, 45.3, and 47.5 mM for 50% 1-propanol/H_2_O, 50% DMSO/H_2_O, and 30% CD_3_CN/H_2_O, respectively (Section S1, Supporting Information).

## Results and Discussion

### Determination of p*K*_a_ of 2,6-DHB

We first determine the p*K*_a_ of 2,6-DHB in the solvent mixture by layering a solution of sodium 3-(trimethylsilyl)-1-propanesulfonate (DSS, 10 mM) on top of 8–9 mg of 2,6-DHB. Dissolution and diffusion of the 2,6-DHB establishes a concentration gradient that can be predicted based on the viscosity of the solvent mixture (Section S2, Supporting Information). In the absence of other acids or bases, the fraction of 2,6-DHB in its deprotonated state, f, is given by eq 1
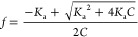
1where *K*_a_ is concentration based and *C* is the total concentration of 2,6-DHB at each position along the sample. Assuming a fast exchange on the ^1^H NMR time scale, the chemical shift of 2,6-DHB, δ_DHB_, is given by
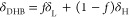
2where δ_H_ and δ_L_ are the limiting chemical shifts of the protonated and deprotonated states, respectively. *C* is obtained by integrating the ^1^H resonance of the 3,5-position of the aromatic ring of 2,6-DHB against the methyl resonance of DSS at each position along the sample using CSI. The molar ionic strength, *I*, of the solution at each position is calculated as the sum of the concentrations of DSS and dissociated 2,6-DHB. The activity coefficient of a univalent ion, γ, is obtained from eq 3
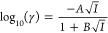
3where *A* is taken as 1.32, 0.546, and 0.755 and *B* as 3.09, 2.22, and 4.45 for 50% 1-propanol/H_2_O, 50% DMSO/H_2_O, and 30% CD_3_CN/H_2_O, respectively (Section S3, Supporting Information).^22–24^ In the absence of activity data, ionic strength can be ignored (*A* = 0) and our method will yield p*K*_a_ values uncorrected for ionic strength (Section S4, Supporting Information). The concentration-based *K*_a_ is calculated from the thermodynamic p*K*_a_ (p*K*_a,0_) using eq 4
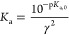
4

The p*K*_a,0_ of 2,6-DHB is obtained by fitting the chemical shift of the 3,5-position to eqs 1–4, with p*K*_a,0_, δ_H_, and δ_L_ as free variables (Figure 1a). p*K*_a,0_ is obtained as 1.61, 1.02, and 1.54 in 50% 1-propanol/H_2_O, 50% DMSO/H_2_O, and 30% CD_3_CN/H_2_O, respectively, in agreement with 1.80 interpolated from the data of Papadopoulos and Avranas in 50% 1-propanol/H_2_O (Section S5, Supporting Information).^17^ Fitting the 4-position of 2,6-DHB yields the same p*K*_a,0_ (Section S6, Supporting Information).

### Determination of p*K*_a_ of 1,2,4-Triazole

To verify that the change in the ^1^H chemical shift of 2,6-DHB with concentration is due to dissociation, the experiment is repeated in the presence of 40 mM 1,2,4-triazole. The pH is calculated from eq 5 using the values of p*K*_a,0_, δ_H_, and δ_L_ determined for 2,6-DHB in the absence of triazole.
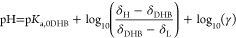
5

The ionic strength is calculated from the ^1^H chemical shift of 2,6-DHB and the concentration of DSS (eq 6).
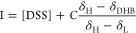
6

The p*K*_a,0_ of 1,2,4-triazole is obtained by fitting the chemical shift of the CH resonance of triazole (δ_T_) to eq 7.
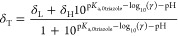
7δ_L_ is taken as the ^1^H chemical shift of 1,2,4-triazole in the solution prior to layering on top of the 2,6-DHB. Fitting to eq 7 is only performed for points where the uncertainty in the pH determined from the chemical shift of 2,6-DHB is less than 0.1 units based on the parameters of 2,6-DHB determined in the absence of triazole. To verify the p*K*_a,0_ of 2,6-DHB, *f* is calculated from the pH and total concentration (*T*, 40 mM) of 1,2,4-triazole using eq 8 (Section S7, Supporting Information).
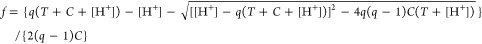
8where  and [H^+^] = γ^–1^10^–pH^. The chemical shift of 1,2,4-triazole is fitted to eq 7 with p*K*_a,0 triazole_ and δ_H_ as free parameters and the chemical shift of 2,6-DHB to eqs 2 and 8 with p*K*_a,0 DHB_*, δ_H_, and δ_L_ as free parameters. The uncertainty in the p*K*_a,0_ of 2,6-DHB is taken as the difference between pK*_a,0 DHB_ and p*K*_a,0 DHB_ as in the ideal case, the two values are equal. This uncertainty is used to calculate the average uncertainty in the pH in the fitting of eq 7, which is taken as the uncertainty in p*K*_a,0 triazole_ (Section S7, Supporting Information). Fitting data for the three solvent mixtures yield p*K*_a,0_ values of triazole of 1.80 ± 0.36, 1.77 ± 0.19, and 2.33 ± 0.41 in 50% 1-propanol/H_2_O, 50% DMSO/H_2_O, and 30% CD_3_CN/H_2_O, respectively (Figure 1b). The p*K*_a_ values determined by homogeneous mixing of DSS, 2,6-DHB, and triazole agree with the CSI-derived values within fitted uncertainties (Section S1, Supporting Information).

### Determination of p*K*_a_ of Indicators

Having determined the p*K*_a,0_ of 2,6-DHB and 1,2,4-triazole in a solvent mixture, we can now obtain δ_H_, δ_L_, and p*K*_a,0_ for a series of organic molecules with p*K*_a_ values up to 10. This set of indicators allows us to determine pH up to 12 via their ^1^H chemical shifts.^18^ We chose a set of molecules possessing only one or two singlet ^1^H resonances: DMG, glycolate, acetate, IM, and 2-methylimidazole (2MI). Sodium was the counterion in all samples. To determine δ_H_ and p*K*_a,0_ for these indicators, two CSI experiments were run to span acidic (pH < 6) and basic (pH > 6) ranges. Salicylate and 4-cyanophenol (4CN) were used as additional indicators in these experiments. For the acidic range, 1,2,4-triazole (20 mM), DMG sodium salt (2 mM), glycolate (20 mM), acetate (20 mM), and salicylate (2 mM) were layered on top of 4–5 mg of 2,6-DHB. For 50% 1-propanol/H_2_O, DMG was excluded due to spectral overlap with 1-propanol. Salicylate was included as an analyte only in 30% CD_3_CN/H_2_O and in 50% 1-propanol/H_2_O, where it was included at a concentration of 20 mM.

The pH value reported by each indicator, pH_*i*_, for which δ_H_, δ_L_, and p*K*_a,0_ are known (initially only 2,6-DHB and 1,2,4-triazole) is calculated using eq 9
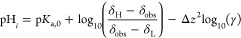
9where δ_obs_ is the observed chemical shift of the indicator and Δ*z*^2^ is the difference in the square of the charge of the indicator between the protonated and deprotonated states (−1 and 1 for 2,6-DHB and 1,2,4-triazole, respectively). For these experiments, δ_L_ of 1,2,4-triazole was redetermined in the solution of indicators from a row of the CSI data set where the pH was sufficiently low for the anionic form to be absent and the triazole to be in its neutral form, as judged relative to the ^1^H chemical shift of acetate (Figure S22, Supporting Information). γ was calculated using eq 3, with *I* at each point along the sample taken as the sum of the ionic strength of the solution of ligands in the absence of 2,6-DHB (*I*_0_), the concentration of H^+^, and the concentration of protonated nitrogen bases (eq 10)
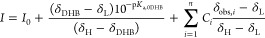
10where the summation is carried out for all nitrogen bases of concentration *C*_*i*_ for which δ_H_, δ_L_, and p*K*_a,0_ are known. The sensitivity, *S*_*i*_, of the chemical shift of each indicator with respect to pH is calculated using eq 11(18,25)
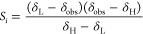
11

The pH value at each position along the sample is calculated as the sensitivity-weighted average of pH_*i*_ calculated from each indicator for which δ_H_, δ_L_, and p*K*_a,0_ are known.
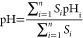
12where the summation is carried out for all indicators where the uncertainty in pH_*i*_ arising from the uncertainty in δ_obs_, δ_H_, and δ_L_ of the indicator is less than 0.05 units (Section S7, Supporting Information). This cutoff was increased to 0.1 units for the calibration of IM in 50% 1-propanol/H_2_O due to the large uncertainty in pH determined from the known indicators (acetate, glycolate, triazole, and 2,6-DHB) when the pH approached the p*K*_a_ of IM. To determine δ_H_, δ_L_, and p*K*_a,0_ for a new indicator, initial values are used to calculate pH_*i*_ for the indicator which is included in the calculation of the overall pH using eqs 9–12, provided the uncertainty in pH_*i*_ arising from the uncertainty in δ_obs_, δ_H_, and δ_L_ is less than 0.4 units. δ_H_ and p*K*_a,0_ of the indicator are obtained by fitting δ_obs_ to eq 13 for each point along the sample for which pH can be determined using the known indicators.
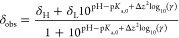
13

δ_L_ was taken as the ^1^H chemical shift measured in the solution of indicators prior to layering on 2,6-DHB, with the exception of DMG where δ_L_ for the acidic step was judged from the CSI data set relative to the ^1^H chemical shift of acetate (Figure S22, Supporting Information). The 0.4 units cutoff in the calculation of pH_*i*_ for the new indicator was increased to 0.8 if no points with acceptable uncertainty in pH_*i*_ were detected following fitting to eq 13, so that an uncertainty in p*K*_a,0_ could be calculated. This was only the case for 4CN in 50% 1-propanol/H_2_O. Inclusion of the new indicator in the calculation of pH from eq 12 helps avoid the fitting to eq 13 being thrown by the high uncertainty when pH determined from the known indicators approaches their upper limit of quantitation. The uncertainty in p*K*_a,0_ for an indicator is calculated as the highest uncertainty in p*K*_a,0_ of the known indicators, plus the average difference between pH_*i*_ of the new indicator and the pH calculated from eq 12 using only the known indicators. Once δ_H_, δ_L_, and p*K*_a,0_ and the uncertainty in p*K*_a,0_ have been determined, the parameters of the next indicator with the higher p*K*_a_ can be determined until the full set is established. For 50% DMSO/H_2_O, salicylate was used to improve the fitting of glycolate to eq 13 by bridging the gap in the p*K*_a_ between DMG and glycolate.

For the basic pH range, a solution of glycolate (20 mM), acetate (20 mM), IM (20 mM), 2MI (20 mM), 4CN sodium salt (20 mM), and DMG sodium salt (20 mM) was layered on the top of 4–5 mg of 2,6-DHB. Quinine hydrochloride (2 mM) was included as an analyte in the 30% CD_3_CN/H_2_O sample. The 50% DMSO/H_2_O sample contained 2 mM *N*,*N*-bis(2-hydroxyethyl)-2-aminoethanesulfonate (Bes) as an analyte; however, the pH did not reach a sufficiently low value to determine the p*K*_a,0_ of this compound. With the exception of DMG, δ_L_ of the indicator was measured in the solution prior to layering on the top of 2,6-DHB. δ_L_ for the fully deprotonated state of DMG (p*K*_a,0_ > 9) was determined by fitting δ_obs_ to eq 13 with δ_H_, δ_L_, and p*K*_a,0_ as free variables, and δ_L_ constrained to be less than or equal to the chemical shift measured prior to layering on 2,6-DHB. IM and 4CN bridged the gap in p*K*_a_ between acetate and 2MI and between 2MI and DMG, respectively. However, IM was not required as an indicator in 50% DMSO/H_2_O due to the high p*K*_a_ of acetic acid in this solvent mixture.

**Figure 2:**
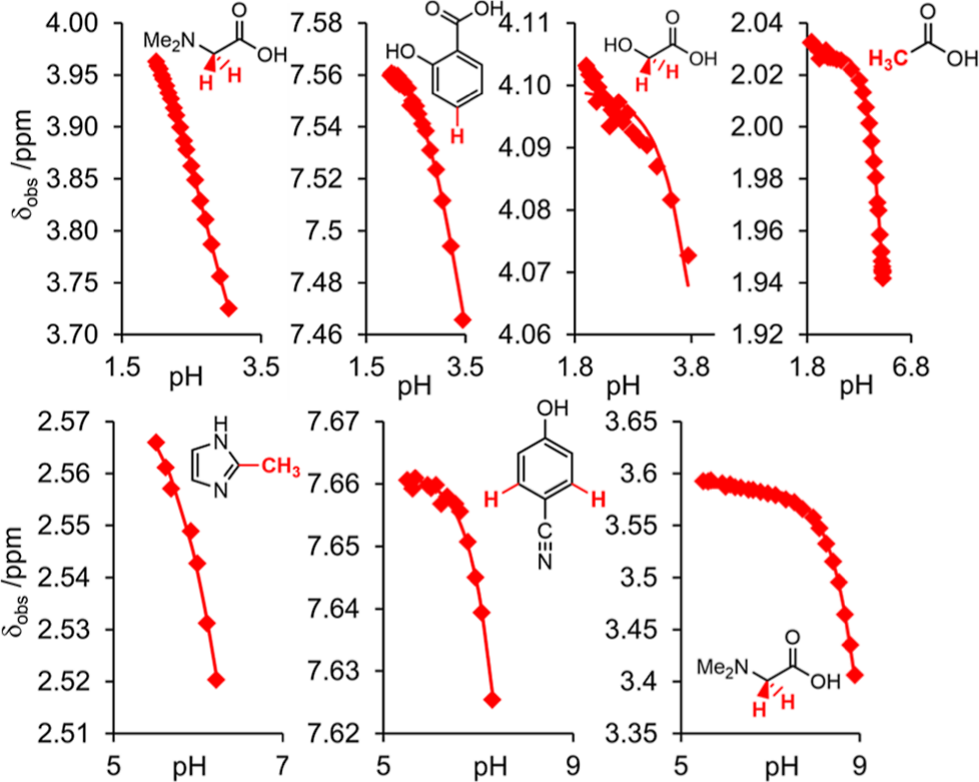
Plot of ^1^H chemical shifts of indicators vs pH in 50% DMSO/H_2_O. Solid lines are fits to eq 13.

The values of δ_H_, δ_L_, and p*K*_a,0_ for the indicators are provided in Table 1. The p*K*_a,0_ values of acetic acid determined in 50% 1-propanol/H_2_O and 30% CD_3_CN/H_2_O (5.47 ± 0.38 and 5.46 ± 0.54, respectively) agree with the literature values of 5.71^26^ and 5.40^6^ reported in these solvent mixtures. The plots of the ^1^H chemical shifts of the indicators and fits to eq 13 in 50% DMSO/H_2_O are provided in Figure 2. The plots for 50% 1-propanol/H_2_O and 30% CD_3_CN/H_2_O are provided in Section S9 of the Supporting Information.

### Determination of p*K*_a_ of Organic Molecules

Having established a set of indicator molecules to determine pH, we can measure the ^1^H chemical shifts of organic analyte molecules and fit them to eq 13 with δ_H_, δ_L_, and p*K*_a,0_ as free variables. With the exception of DMG in the determination of the p*K*_a,0_ of salicylate (50% DMSO/H_2_O and 30% CD_3_CN/H_2_O), all indicators were included at a 20 mM concentration, as sodium salts or neutral bases, to produce smooth pH gradients via buffering effects. As p*K*_a,0_ and δ_H_ are known for each indicator, δ_L_ does not need to be redetermined. The pH of a row is calculated by grouping the indicators into pairs in order of p*K*_a,0_ and calculating the sensitivity-weighted average pH_*i*_ of the pair with the highest combined sensitivity.^18^

**Figure 3:**
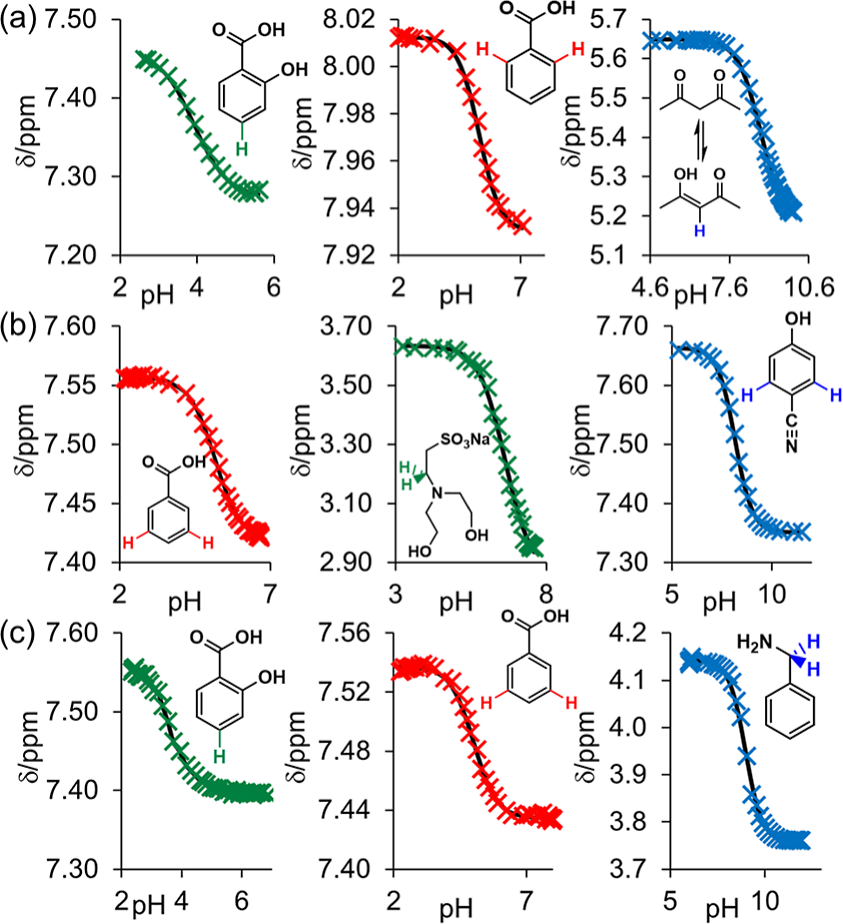
Plot of ^1^H chemical shifts of monoprotic analytes vs pH in 50% 1-propanol/H_2_O (a), 50% DMSO/H_2_O (b), and 30% CD_3_CN/H_2_O (c) and fits to eq 13 (solid lines).

Analytes were 2 mM in 50% DMSO/H_2_O and 30% CD_3_CN/H_2_O and 20 mM in 50% 1-propanol/H_2_O. A total of 4–5 mg of 2,6-DHB was used unless otherwise stated. Good fits to eq 13 are obtained for a range of monoprotic analytes (Figure 3), while the p*K*_a,0_ values obtained agree with values reported in the literature (Table 2). These literature p*K*_a,0_ values have been interpolated from published data to the solvent compositions used in the present work (Section S5, Supporting Information). The uncertainty in the fitted p*K*_a,0_ was taken as the uncertainty in pH at the data point where the pH was closest to the value of p*K*_a,0_ – Δ*z*^2^ log_10_(γ).

The p*K*_a,0_ of the enol form of acetylacetone was determined from the ^1^H chemical shift of the proton on the unsaturated carbon and converted to the overall p*K*_a,0_ of acetylacetone as p*K*_a,0(enol)_ – log_10_(*j*), where *j* is the fraction of compound in the enol form at low pH (Figure S21, Supporting Information).^38^^1^H spectra of analytes are provided in Section S8 of the Supporting Information.

**Figure 4:**
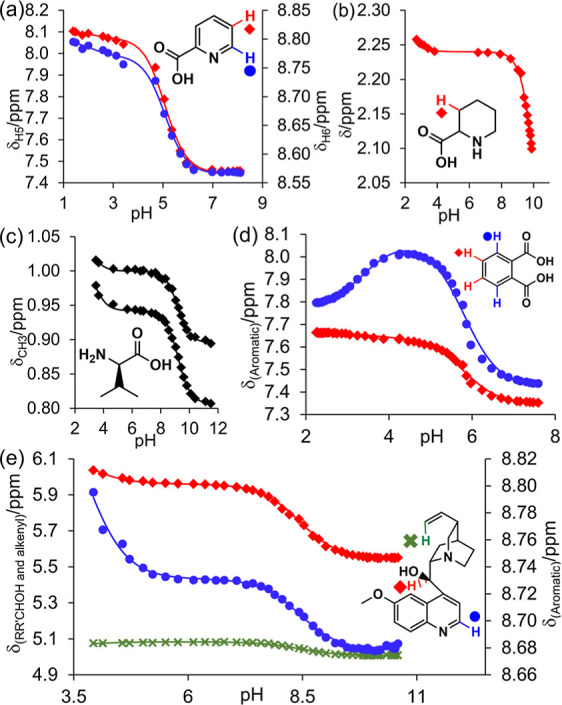
Plot of the ^1^H chemical shifts of diprotic analytes. Solid lines are fits to eq 14. (a) Picolinic acid, 50% 1-propanol/H_2_O. (b) Pipecolic acid, 50% 1-propanol/H_2_O. (c) d-valine, 50% DMSO/H_2_O. (d) Phthalic acid, 30% CD_3_CN/H_2_O. (e) Quinine, 30% CD_3_CN/H_2_O.

For analytes possessing two protonation steps (picolinic acid, pipecolic acid, valine, phthalic acid, and quinine), eq 13 is modified to include two protonation steps
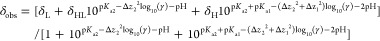
14where the subscript denotes the deprotonation step and δ_L_, δ_HL_, and δ_H_ are the chemical shifts of the fully deprotonated, monoprotonated, and fully protonated states, respectively, and the p*K*_a_ values are thermodynamic. Fits to eq 14 are shown in Figure 4. All p*K*_a_ values and limiting chemical shifts were free fitting parameters. For all compounds, p*K*_a2_ is determined with good accuracy. However, with the exception of phthalic acid, the pH does not attain a sufficiently low value to allow a reliable fitting of p*K*_a1_. Nevertheless, an estimate of p*K*_a1_ is obtained (Table 2). The p*K*_a1_ of valine compares with the value of 3.00 reported by Dogăn et al.^13^ in 40% (v/v) DMSO/H_2_O. For phthalic acid, complete titration curves are recorded, allowing both p*K*_a_ values to be fitted (Figure 4). The large number of pH values assessed in the CSI experiment also reveals the contrasting behavior of the two aromatic resonances.

For quinine, the proton at position 2 of the quinoline ring exhibits a much larger change in chemical shift below pH 6 than the proton adjacent to the hydroxyl, implying that p*K*_a1_ is associated with the protonation of quinoline nitrogen. Such information may not be apparent from a conventional NMR titration where fewer data points are collected or if p*K*_a_ was determined solely by potentiometric methods.

## Conclusions

We have shown how a set of indicators for the measurement of pH in aqueous–organic solvents can be calibrated using four ^1^H CSI experiments, avoiding completely the use of literature data or electrochemical measurements and liberating researchers to study pH-dependent processes at the solvent composition required by their work. The indicators allow the ^1^H spectra of organic molecules, including active pharmaceutical ingredients, to be recorded as a function of pH using CSI techniques. The uncertainty in p*K*_a_ determined by our method is comparable to the spread in the p*K*_a_ values reported in the literature. As well as organic solvents, our method could potentially reveal the effect of additives, such as molecular crowding agents, on p*K*_a_, thus providing a valuable tool to inform experimental design.

## Data Availability

The data underlying this study are openly available at https://research-portal.uea.ac.uk/en/datasets/data-for-measurement-of-the-pka-values-of-organic-molecules-in-aq.

## Supporting Information Available

Integration of 2,6-DHB; analysis of samples at different times since preparation; extraction of *A* and *B* (eq 3); interpolation of p*K*_a_ values from published data; extraction of p*K*_a_ of 2,6-DHB using 4-position; uncertainty analysis; example ^1^H spectra; calibration plots of indicators; automated processing of data sets; and CSI pulse sequences (PDF)
Spreadsheet for analysis of data sets (XLSX)
