# Supplementary material for: Measurement of the pKa Values of Organic Molecules in Aqueous–Organic Solvent Mixtures by 1H NMR without External Calibrants
Source: Anal Chem. 2023 Oct 13;95(42):15628–35. doi: 10.1021/acs.analchem.3c02771 (PMC10603606; doi:10.1021/acs.analchem.3c02771)
Supplement: Supplementary file 1 — ac3c02771_si_001.pdf [file ac3c02771_si_001.pdf]

Supporting Information for:

Measurement of the pK_a values of organic molecules in aqueous-organic solvent mixtures by ^{1}H NMR without external calibrants

Matthew Wallace*, Nduchi Abiama and Miranda Chipembere

School of Pharmacy, University of East Anglia, Norwich Research Park, Norwich, NR4 7TJ, UK

*Corresponding Author: Matthew Wallace, email: matthew.wallace@uea.ac.uk

Contents

S1. Quantification of concentration of 2,6-DHB by integration and analysis of homogeneous samples 2
S2. Analysis of samples at different times since preparation 5
S3. Extraction of A and B of Equation 3 in 1-propanol/H_2O and acetonitrile/H_2O from published mean activity coefficients of HCl 12
S4. Determination of pK_a values of analytes without correction for ionic strength 13
S5. Interpolation of pK_a values from published data 15
S6. Extraction of p$K_{a,0}$ of 2,6-DHB *via* observation of resonance of 4-position 15
S7. Uncertainty analysis in determination of pK_a and pH 16
S7.1 Calculation of uncertainty in p$K_{a,0}$ of 2,6-DHB and 1,2,4-triazole 16
S7.2 Calculation of uncertainty in p$K_{a,0}$ of other indicators 17
S7.3 Calculation of uncertainty in p$K_{a,0}$ of analyte molecules 17
S7.4 Derivation of Equations 8, 13 and 14 17
S8. Example ^{1}H spectra from CSI datasets 20
S9. Calibration plots of indicators in 50% 1-propanol/H_2O and 30% CD_3CN/H_2O 29
References 29
S10. Routines for automated processing of NMR datasets 30
S10.1. Processing using Mnova 14.3.1 30
S10.2 Data acquisition and processing scripts (Bruker) 34
S11. CSI pulse sequences (Bruker) 43
S11.1 Spin-echo sequence (no solvent suppression) 43
S11.2 CSI sequence with perfect-echo water suppression 44

S1. Quantification of concentration of 2,6-DHB by integration and analysis of homogeneous samples

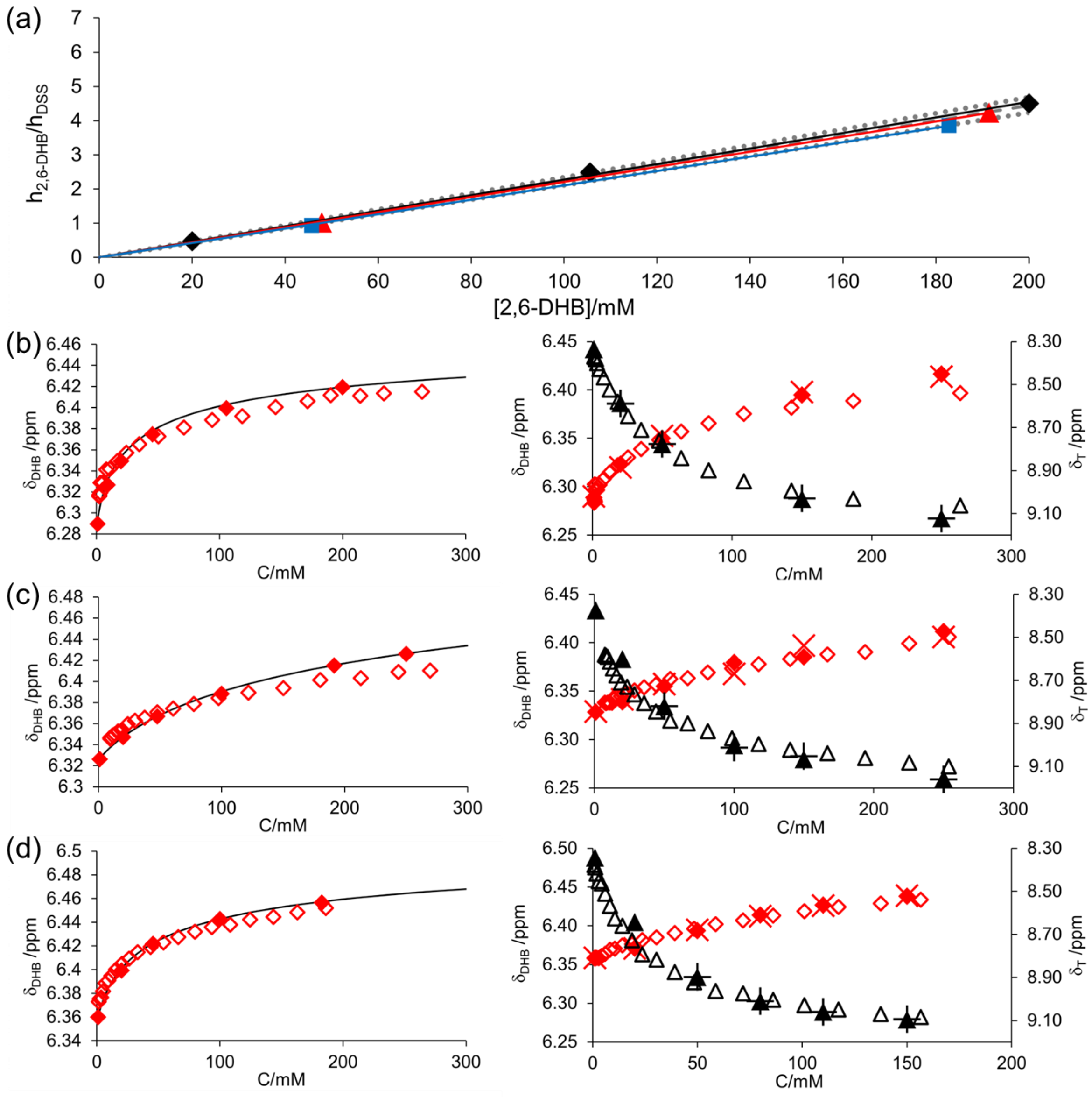

Figure S1. (a) Plot of ratio of ^{1}H integral of 2,6-DHB (3,5-position) to 10 mM DSS (methyl) versus known concentration of 2,6-DHB in 50% 1-propanol/H_2O (black diamond), 50% DMSO/H_2O (red triangle) and 30% CD_3CN/H_2O (blue square). Straight line fits pass through origin. Theoretical ratio based on the ratio of protons of 2,6-DHB and DSS (k = 45 mM, solid line), and this theoretical ratio ±5% (grey dotted lines). **(b-d) Left:** Plots of ^{1}H chemical shift of 2,6-DHB versus known concentration in homogeneous samples of 2,6-DHB and 10 mM DSS (red diamond), fit of homogeneous data to Equations 1-4 (black line), chemical shift of 2,6-DHB measured in CSI experiment versus apparent concentration determined by integration of resonance against DSS along concentration gradient (open symbols, data also plotted on Figure 1a). **Right:** Plots of ^{1}H chemical shifts of 2,6-DHB (red diamond) and 1,2,4-triazole (black triangle) versus known concentration of 2,6-DHB in homogeneous samples that contained 40 mM 1,2,4-triazole and 10 mM DSS (solid symbols), fits to Equation 7 (black vertical cross) and Equations 2 and 8 (red cross), ^{1}H chemical shifts of 2,6-DHB and 1,2,4-triazole measured in CSI experiment against apparent concentration of 2,6-DHB determined by integration of resonance against DSS (open symbols, data also plotted on Figure 1b). (b) 50% 1-propanol/H_2O, (c) 50% DMSO/H_2O, (d) 30% CD_3CN/H_2O.

k (Experimental Section) was determined by analysis of homogeneous samples of known concentration (Figure S1a). The concentration of 2,6-DHB in the stock solutions used for the homogeneous samples in Figure S1 was determined by volumetric titration against NaOH using bromothymol blue as indicator. Concentrations of 2,6-DHB in these experiments are assumed accurate to 5%. Spectra to determine the relationship between the integral and concentration of 2,6-DHB in 50% 1-propanol/H_2O (Figure S1a) were recorded with the spin-echo sequence used for CSI but without an encoding gradient pulse (τ = 381 μs), and an acquisition time and relaxation delay of 3.27 s and 1.88 s, respectively. Analogous spectra in 50% DMSO/H_2O and 30% CD_3CN/H_2O were recorded using the Bruker library sequence zgesgppe, with the same parameters as for CSI, but without the phase encoding gradient.

Table S1. $pK_{a,0}$, δ_H and δ_L for 2,6-DHB and 1,2,4-triazole determined from homogeneous samples of DSS, triazole and known concentrations of 2,6-DHB (Figure S1b-d, solid symbols), and parameters of other NMR indicators determined in CSI experiments using the homogeneous-derived parameters of 2,6-DHB and triazole.

50% 1-propanol/H_2O				50% DMSO/H_2O			30% CD_3CN/H_2O		
Indicator	$pK_{a,0}$	δ_H/ppm	δ_L/ppm	$pK_{a,0}$	δ_H/ppm	δ_L/ppm	$pK_{a,0}$	δ_H/ppm	δ_L/ppm
2,6-DHB[a]	1.80±0.11	6.4904	6.2833	0.80±0.30	6.5956	6.3253	1.53±0.11	6.5209	6.3558
1,2,4-triazole	1.70±0.13	9.3368	8.3164[b]	1.45±0.34	9.3791	8.3621[b]	2.14±0.10	9.2159	8.3278[b]
DMG[c]	-	-	-	2.40±0.34	4.0443	3.5755	2.28±0.11	4.0169	3.6370
Salicylic acid	-	-	-	3.10±0.37	7.5689	7.3772	-	-	-
Glycolic acid	4.41±0.14	4.1724	3.9171[d]	4.59±0.45	4.0994	3.7583[d]	4.28±0.14	4.1718	3.8643[d]
Acetic acid	5.42±0.16	2.0517	1.9160[d]	5.49±0.46	2.0293	1.8048[d]	5.31±0.20	2.0583	1.8485[d]
IM	5.88±0.22	8.7794	7.7155	-	-	-	6.46±0.25	8.6442	7.7254
2MI	6.82±0.24	2.6227	2.3574	6.44±0.49	2.5790	2.3181	7.40±0.28	2.5717	2.3233
4CN	8.49±0.25	7.5160	7.2840	8.06±0.52	7.6617	7.3510	8.28±0.30	7.6370	7.4052
DMG	9.45±0.27	2.9373[e]	2.2333[e]	9.22±0.62	3.5870[c]	2.8685[c]	9.67±0.38	3.6511[c]	2.9289[c]

[a]$pK_{a,0}$, δ_L and δ_H obtained in absence of 1,2,4-triazole using Equations 1-4, uncertainty obtained from experiment with 40 mM 1,2,4-triazole. [b]Average of δ_L determined in solution of triazole (40 mM) and DSS, and in acidic range sample. [c]CH_2 resonance of DMG. [d]Average of acidic and basic range samples in absence of 2,6-DHB. [e]Methyl resonance of DMG.

Table S2. Comparison of p$k_{a,0}$ of analyte molecules determined by ^{1}H CSI using parameters of 2,6-DHB and 1,2,4-triazole determined from homogeneous samples of DSS, triazole and known concentrations of 2,6-DHB (Figure S1b-d solid symbols, values for all indicators provided in Table S1) (p$K_{a,\ Homog}$), and using parameters of 2,6-DHB and 1,2,4-triazole determined by CSI (p$K_{a,0\ CSI}$, as provided on Table 2).

50% 1-propanol/H_2O				50% DMSO/H_2O				30% CD_3CN/H_2O			
Analyte	Indicator	p$K_{a,0}$ CSI	p$K_{a,0}$ Homog	Analyte	Indicator	p$K_{a,0}$ CSI	p$K_{a,0}$ Homog	Analyte	Indicator	p$K_{a,0}$ CSI	p$K_{a,0}$ Homog
Salicylic acid[a]	2,6-DHB, triazole, glycolate, acetate	4.12 ±0.37	4.08 ±0.16	Salicylic acid[a]	2,6-DHB, Triazole, DMG, glycolate, acetate	3.46 ±0.21	3.23 ±0.35	Salicylic acid[a]	2,6-DHB, Triazole, DMG, glycolate, acetate	3.69 ±0.42	3.53 ±0.12
Benzoic acid[b]	2,6-DHB, triazole, glycolate, acetate, 2MI	5.52 ±0.38	5.46 ±0.16	Benzoic acid	Triazole, glycolate, acetate, 2MI	5.25 ±0.31	5.02 ±0.45	Benzoic acid	Triazole, glycolate, acetate, 2MI	5.10 ±0.49	4.94 ±0.18
Picolinic acid[b]	2,6-DHB, triazole, glycolate, acetate, 2MI	1.85[c] 5.29 ±0.38	1.56[c] 5.24 ±0.16	Bes[d]	Triazole, glycolate, acetate, 2MI	6.72 ±0.34	6.49 ±0.49	Phthalic acid	Triazole, glycolate, acetate, 2MI	3.48 ±0.43, 6.07 ±0.51	3.32 ±0.12, 5.92 ±0.21
Acetylacetone	IM, 2MI, DMG	9.23 ±0.50[e]	9.17 ±0.28[e]	4CN[f]	2MI, DMG	8.25 ±0.45	8.03 ±0.59	Quinine[g]	DMG, glycolate, acetate, IM, 2MI, DMG	3.55[c] 8.35 ±0.65	3.38[c] 8.19 ±0.34
Pipecolic acid	Triazole, 2MI, DMG	2.33[c] 10.34 ±0.50	2.26[c] 10.29 ±0.27	D-valine[f]	2MI, DMG	3.29[c] 9.29 ±0.47	3.05[c] 9.06 ±0.62	Benzylamine[h]	DMG, 2MI, DMG	8.89 ±0.69	8.74 ±0.38

[a]Acidic-range dataset. [b]8-9 mg 2,6-DHB. [c]Approximate pK_{a1} from fitting to Equation 14. [d]Sample also contained DMG sodium salt (2 mM) and tricine (2 mM), formate (4 mM), *tert*-butylamine (10 mM) which were found unsuitable for use as indicators. 5-6 mg 2,6-DHB. [e]Value corrected for enol-ketone tautomerization. [f]Sample also contained NaOH (10 mM), D-valine Na salt (2 mM) and 4CN sodium salt (20 mM). [g]Basic-range dataset. [h]Sample contained NaOH (10 mM) in addition to indicators. 3-4 mg 2,6-DHB.

S2. Analysis of samples at different times since preparation

Assuming Gaussian diffusion, the concentration, C_Z, at distance Z from the diffusing acid is given by Equation S1:[100]

$$C_Z = \frac{m}{\pi r^2 M_r \sqrt{\pi D t}} \exp\left({-Z^2}/{4Dt_{opt}} \right) \qquad S1$$

where r is the radius of the NMR tube (2.1 mm) and M_r the molecular mass of 2,6-DHB. The time at which a ratio of C_b/C_0 will establish is given by Equation S2:[101]

$$t = \frac{{Z_0}^2 - {Z_b}^2}{4D\ln\left(\frac{C_b}{C_0}\right)} \qquad S2$$

where D is the diffusion coefficient of 2,6-DHB. The self-diffusion coefficient of 2,6-DHB at 298 K was measured in 50% 1-propanol/H_2O as 2.8 x 10^{-10} m^2s^{-1} using a double stimulated echo pulse sequence, with a diffusion delay and gradient pulse of 0.2 s and 2.4 ms, respectively. D is corrected for the ambient temperature of our NMR laboratory (295 K) using the Stokes-Einstein equation:

$$D = \frac{K_b T}{6\pi\eta R_h} \qquad S3$$

where η is taken as 2.6, 3.0 and 0.86 mPa.s for 50% 1-propanol/H_2O, 50% DMSO/H_2O and 30% CD_3CN/H_2O, respectively, and is uncorrected for temperature.[102-104] R_h for 2,6-DHB is obtained as 0.3 nm. Combining Equations S2 and S3, we obtain:

$$t = \frac{6\pi R_h}{4K_b T}\eta\left({Z_0}^2 - {Z_b}^2\right) \qquad S4$$

where the term $\frac{6\pi R_h}{4K_b T}$ has a value of 347 $mPa^{-1}mm^{-2}$ at 22 °C. α (Experimental Section) can thus be taken as 0.1 hours.$mPa^{-1}s^{-1}mm^{-2}$ at 22 °C.

The diffusion of 2,6-DHB up the NMR tube is in reasonable agreement with Equation S1 in terms of the concentration ranges spanned (Figure S2) while the $pK_{a,0}$ values obtained over the time window agree within the experimental uncertainties later obtained in the experiment with 1,2,4-triazole. The experiments used to determine $pK_{a,0\ DHB}$ (Figure S3) and $pK_{a,0\ triazole}$ (Figure S4) were chosen as the datasets with the largest concentration range, and with points at a low concentration of 2,6-DHB.

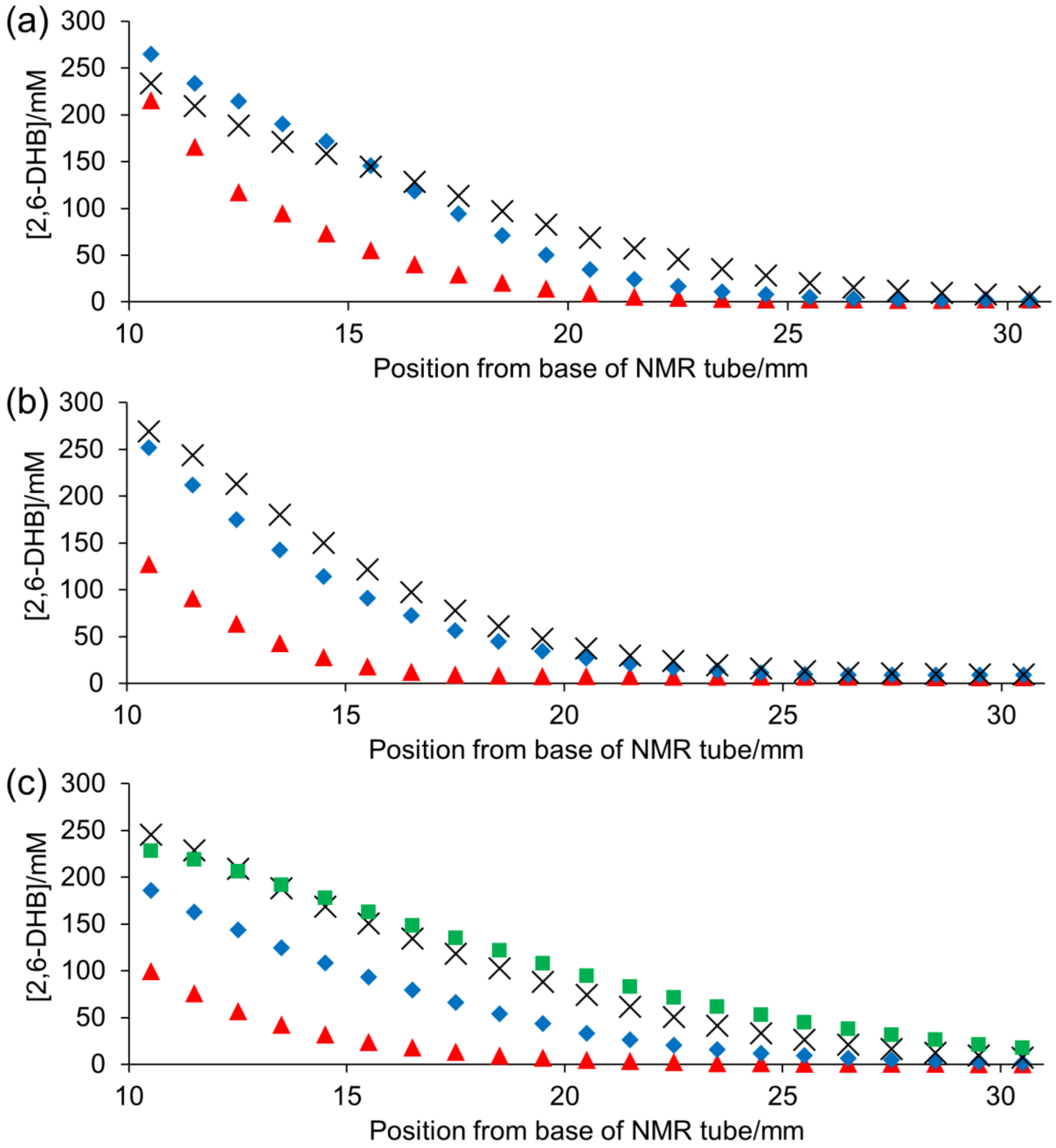

Figure S2. Plots of concentration of 2,6-DHB versus vertical position from absolute base of NMR tube at different times since a 10 mM solution of DSS was placed on top 8-9 mg of solid 2,6-DHB. (a) 50% 1-propanol/H_2O: 11.5 hours (red triangle), 17.5 hours* (blue diamond) and 28 hours (black cross). (b) 50% DMSO-d_6/H_2O: 15.4 hours (red triangle), 33.4 hours (blue diamond) and 39.4 hours* (black cross). (c) 30% CD_3CN/H_2O: 4.5 hours (red triangle), 8.5 hours* (blue diamond), 12.5 hours (black cross) and 30.5 hours (green square). *denotes experiments used to determine p$K_{a,0}$ of 2,6-DHB in main text.

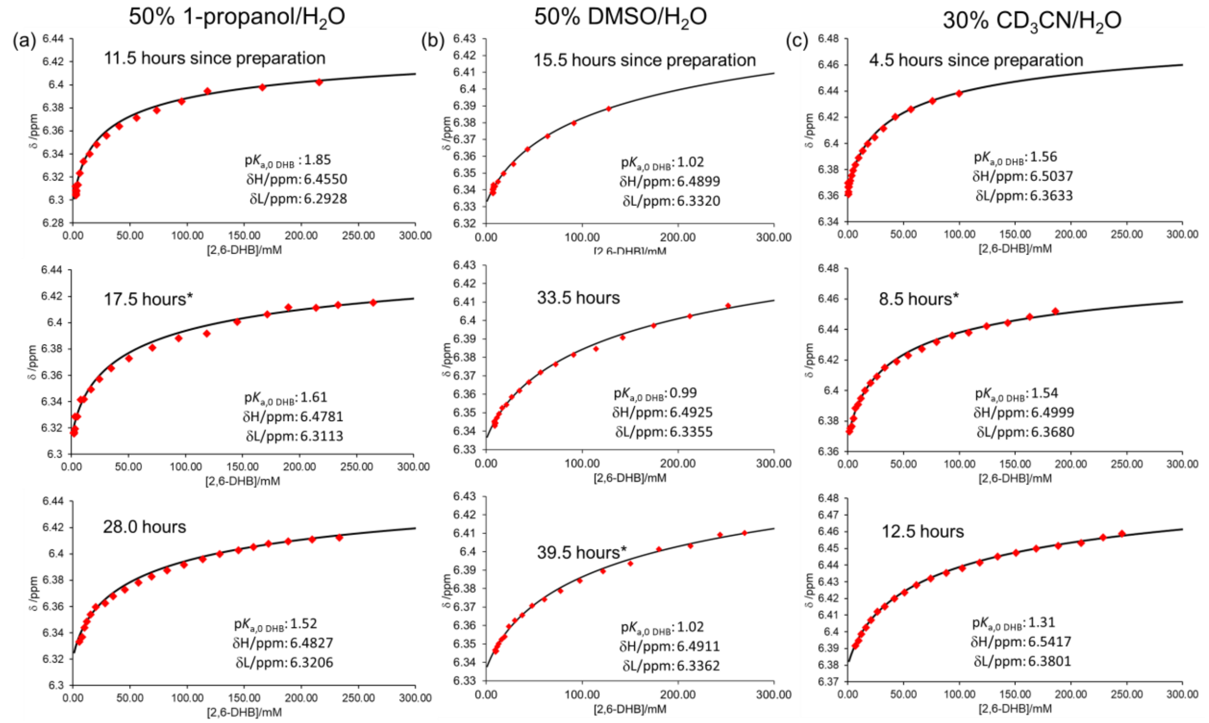

Figure S3. Plot of ^{1}H chemical shift of 2,6-DHB (3,5-position) versus concentration of 2,6-DHB (C, Equation 1). Solid lines are fits to Equation 1-4. *denotes experiments used to determine p$K_{a,0}$ of 2,6-DHB in main text.

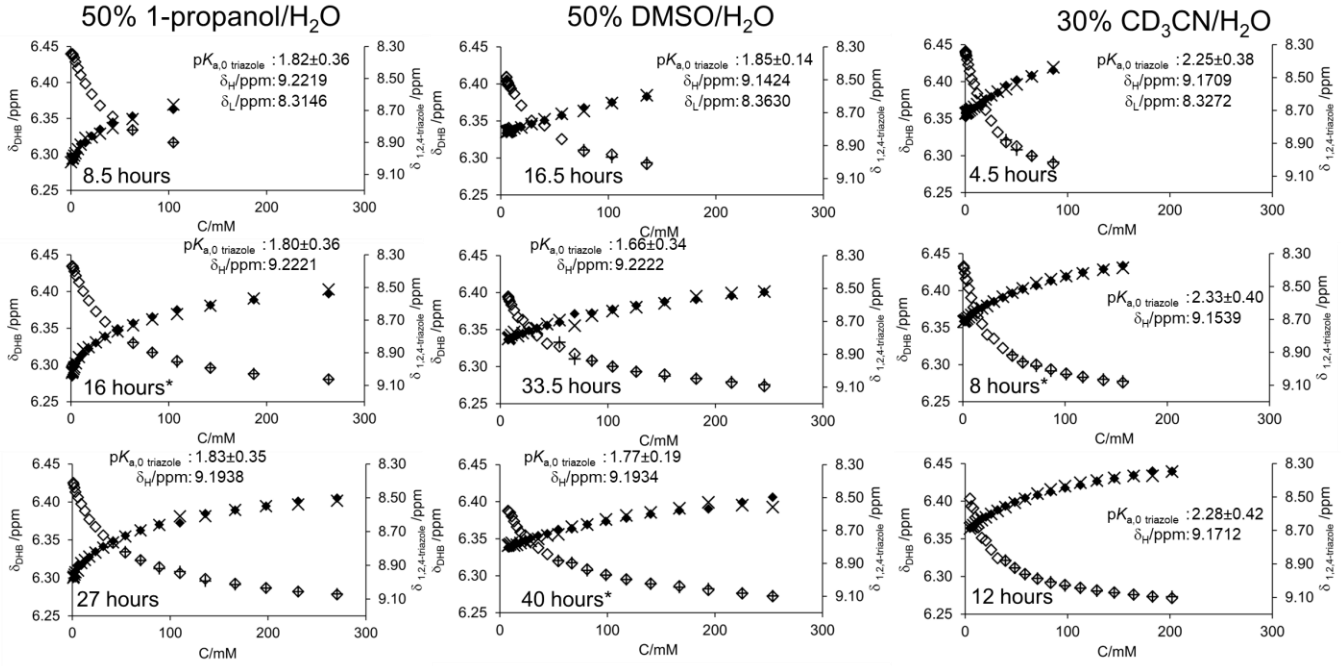

Figure S4. Plots of ^{1}H chemical shift of 2,6-DHB (solid symbols) and 1,2,4-triazole (CH resonance, open symbols) versus concentration of 2,6-DHB. Fits to Equation 7 (vertical cross), and Equations 2 and 8 (diagonal cross). *denotes experiments used to determine p$K_{a,0}$ of 1,2,4-triazole.

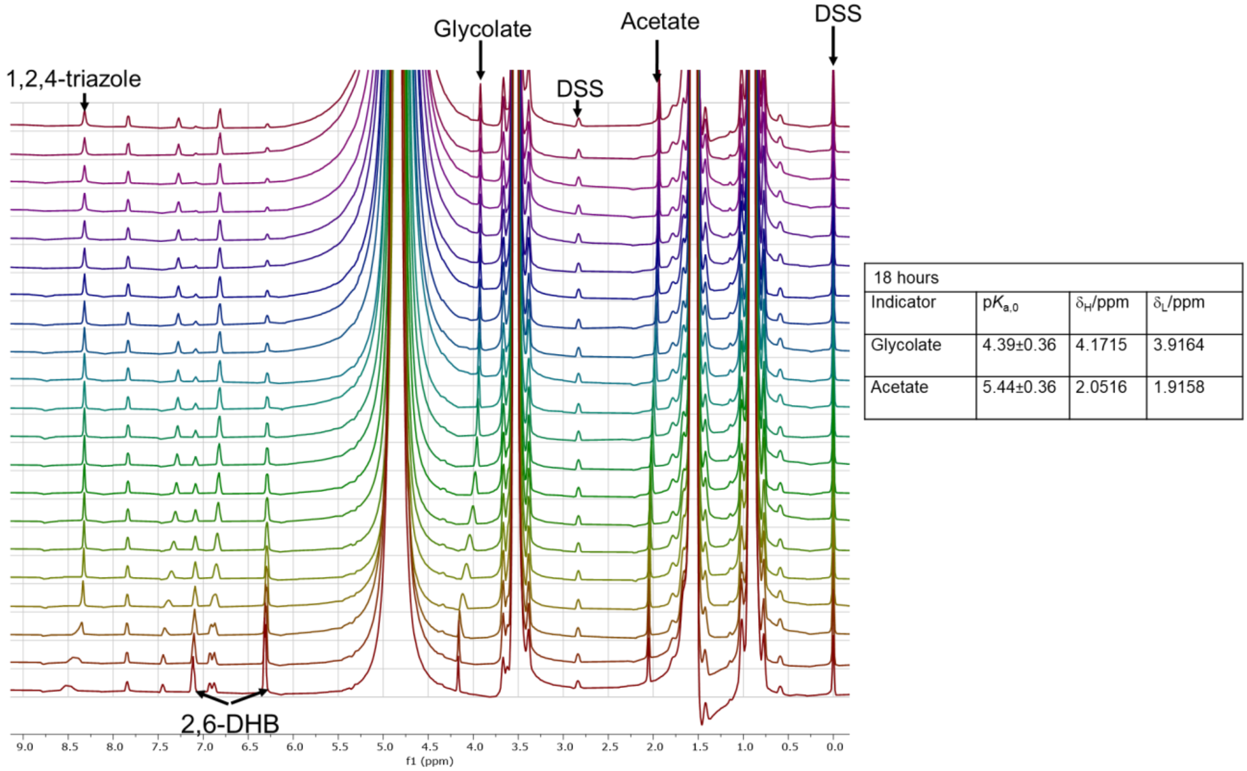

18 hours			
Indicator	p$K_{a,0}$	δ_H/ppm	δ_L/ppm
Glycolate	4.39±0.36	4.1715	3.9164
Acetate	5.44±0.36	2.0516	1.9158

Figure S5. Partial ^{1}H spectra of acidic-range CSI datasets to determine p$K_{a,0}$ values of indicators in 50% 1-propanol/H_2O, 18 hours after preparation. Dataset recorded at 35 hours (Figure S18) used to determine p$K_{a,0}$ values listed in Table 1.

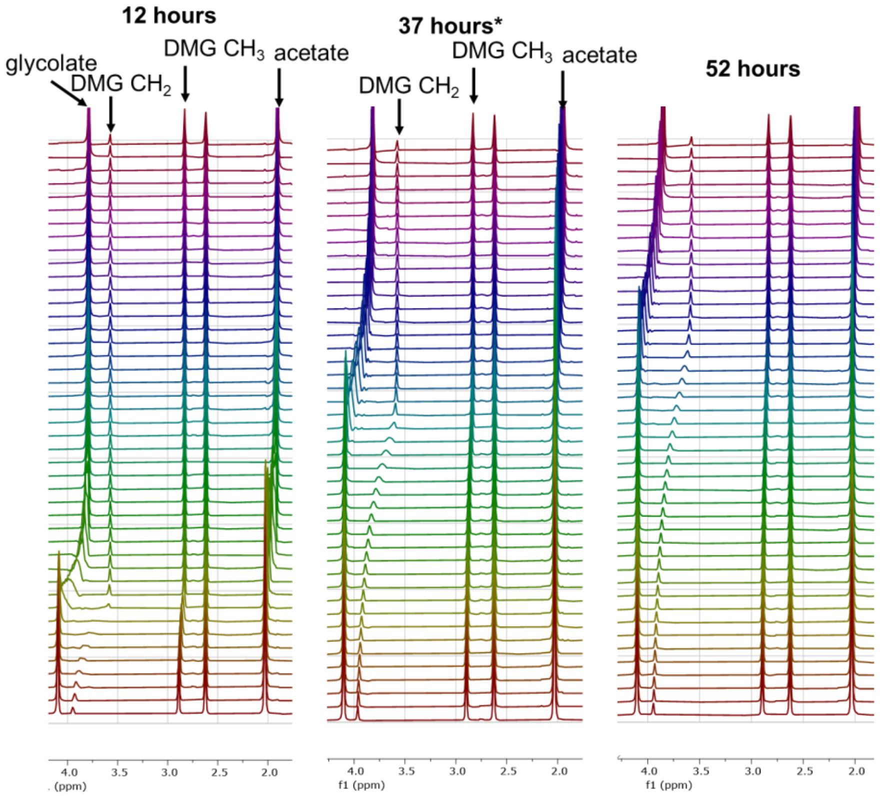

12 hours			
Indicator	p$K_{a,0}$	δ_H/ppm	δ_L/ppm
DMG	2.69±0.23	4.0224	3.5755
Salicylate	3.36±0.36	7.5702	7.3772
Glycolate	4.69±0.43	4.0985	3.7570
Acetate	5.63±0.47	2.0269	1.8038

52 hours			
Indicator	p$K_{a,0}$	δ_H/ppm	δ_L/ppm
DMG	2.64±0.21	4.0340	3.5755
Salicylate	3.35±0.22	7.5669	7.3772
Glycolate	4.82±0.29	4.0992	3.7570
Acetate	5.88±0.31	2.0287	1.8038

Figure S6. Partial ^{1}H spectra of acidic-range CSI datasets to determine p$K_{a,0}$ values of indicators in 50% DMSO/H_2O. Dataset marked * is used to determine p$K_{a,0}$ values listed in Table 1.

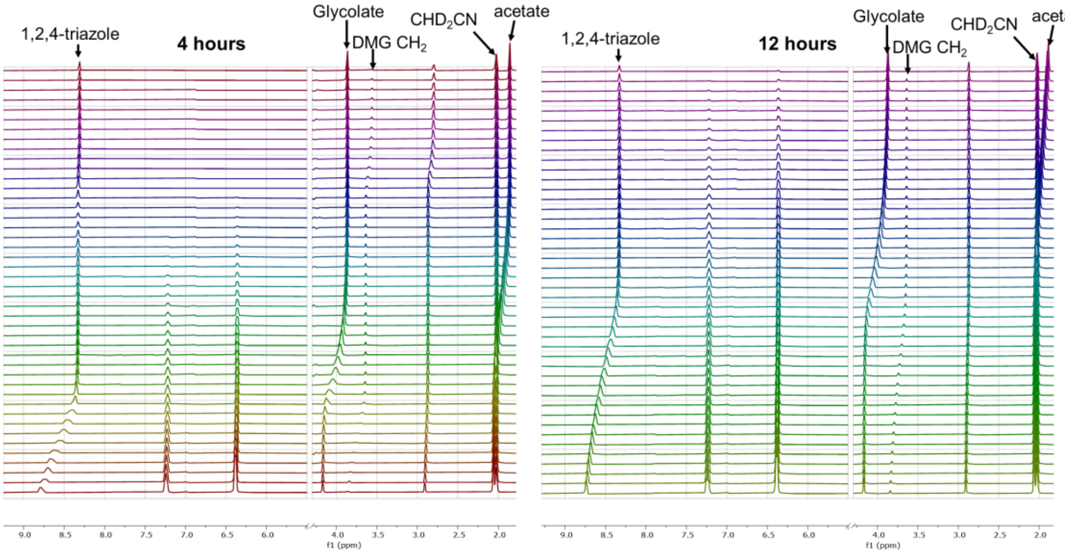

4 hours			
Indicator	$pK_{a,0}$	δ_H/ppm	δ_L/ppm
DMG	2.47±0.42	3.9919	3.6370
Glycolate	4.43±0.45	4.1708	3.8621
Acetate	5.48±0.51	2.0572	1.8360

12 hours			
Indicator	$pK_{a,0}$	δ_H/ppm	δ_L/ppm
DMG	2.47±0.41	3.9933	3.6370
Glycolate	4.45±0.44	4.1714	3.8621
Acetate	5.46±0.47	2.0581	1.8360

Figure S7. Partial ^1H spectra of acidic-range CSI datasets to determine $pK_{a,0}$ values of indicators in 30% CD_3CN/H_2O. Dataset recorded at 8 hours used to determine $pK_{a,0}$ values listed in Table 1.

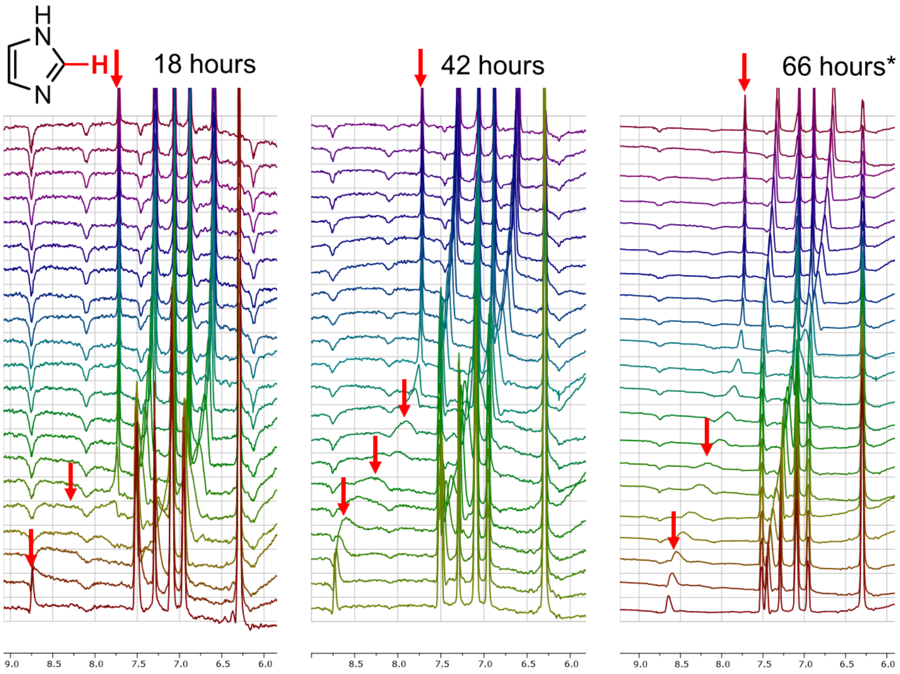

42 hours			
Indicator	$pK_{a,0}$	δ_H/ppm	δ_L/ppm
Imidazole	6.06±0.42	8.7657	7.7155
2MI	7.11±0.48	2.6211	2.3574
4CN	8.81±0.57	7.5159	7.2840
DMG	9.74±0.60	2.9374	2.3042

Figure S8. Partial ^1H spectra of basic-range CSI datasets to determine $pK_{a,0}$ values of indicators in 50% 1-propanol/H_2O. The 2-position resonance of imidazole is indicated and is too broad to observe at 18 hours due to the sharp pH gradient. $pK_{a,0}$ values determined at 42 and 66 hours agree within experimental uncertainties. Dataset marked * is used to determine $pK_{a,0}$ values listed in Table 1 (Figure S19).

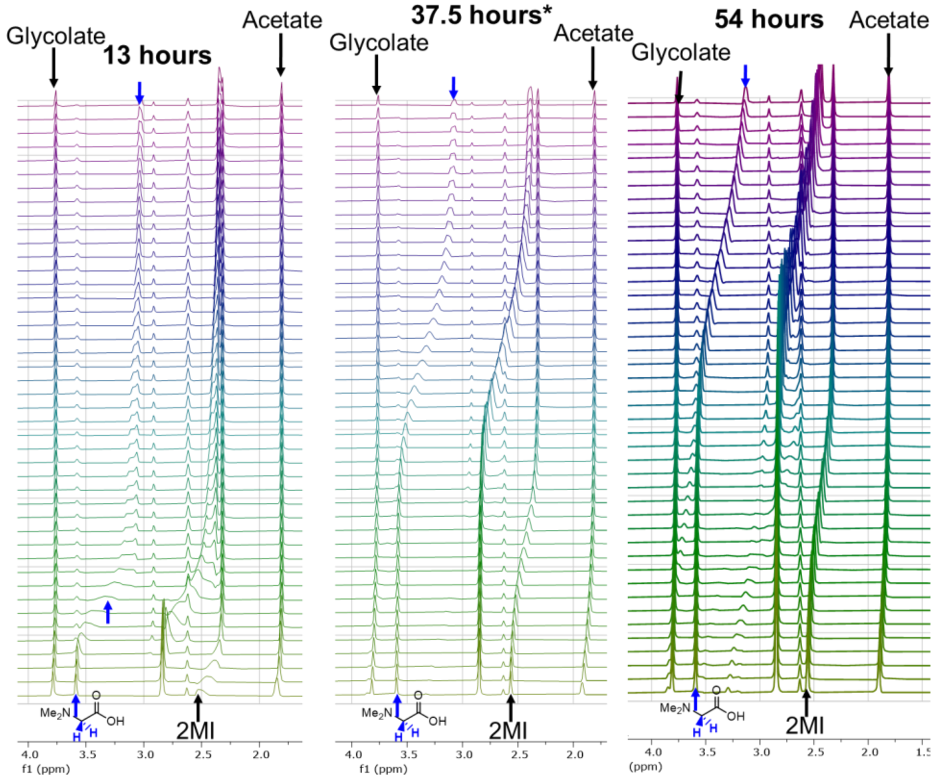

13 hours			
Indicator	p$K_{a,0}$	δ_H/ppm	δ_L/ppm
2MI	6.39±0.33	2.5946	2.3181
4CN	8.02±0.33	7.6653	7.3510
DMG	9.30±0.41	2.9374	2.3042

54 hours			
Indicator	p$K_{a,0}$	δ_H/ppm	δ_L/ppm
2MI	6.67±0.36	2.5768	2.3181
4CN	8.29±0.39	7.6626	7.3510
DMG	9.44±0.49	3.5874	2.8685

Figure S9. Partial ^{1}H spectra of basic-range CSI datasets to determine p$K_{a,0}$ values of indicators in 50% DMSO/H_2O. The methyl resonance of 2MI and the CH_2 resonance of DMG are broadened at 13 hours due to the sharp pH gradient. Dataset marked * is used to determine p$K_{a,0}$ values listed in Table 1.

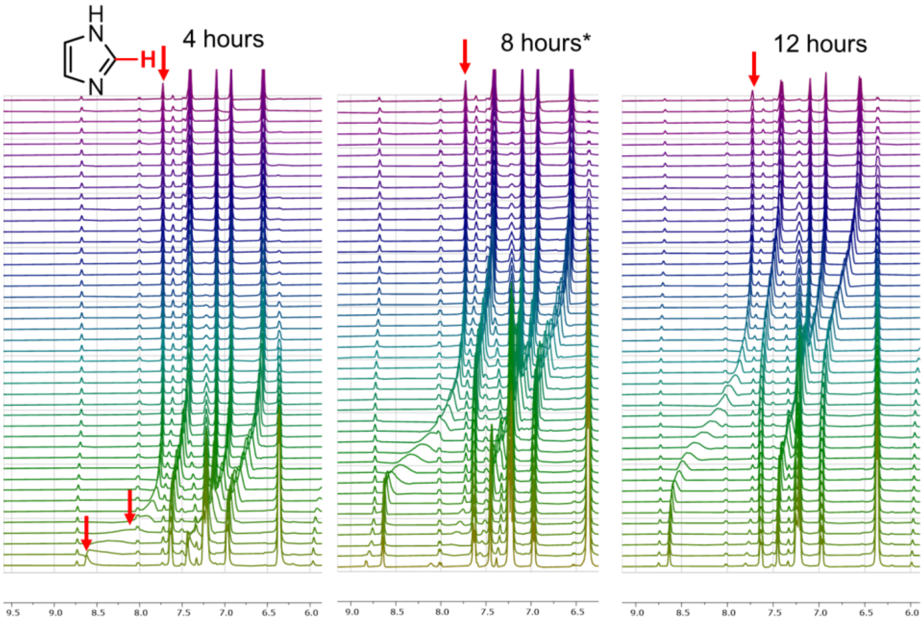

4 hours			
Indicator	p$K_{a,0}$	δ_H/ppm	δ_L/ppm
Imidazole	6.85±0.50	8.6476	7.7254
2MI	7.80±0.58	2.5754	2.3233
4CN	8.65±0.61	7.6410	7.4052
DMG	10.06±0.65	3.6498	2.9289

12 hours			
Indicator	p$K_{a,0}$	δ_H/ppm	δ_L/ppm
Imidazole	6.62±0.54	8.6440	7.7254
2MI	7.56±0.58	2.5712	2.3233
4CN	8.44±0.61	7.6367	7.4052
DMG	9.79±0.72	3.6538	2.9289

Figure S10. Partial ^{1}H spectra of basic-range CSI datasets to determine p$K_{a,0}$ values of indicators in 30% CD_3CN/H_2O. The 2-position resonance of imidazole is indicated. Dataset marked * is used to determine p$K_{a,0}$ values listed in Table 1.

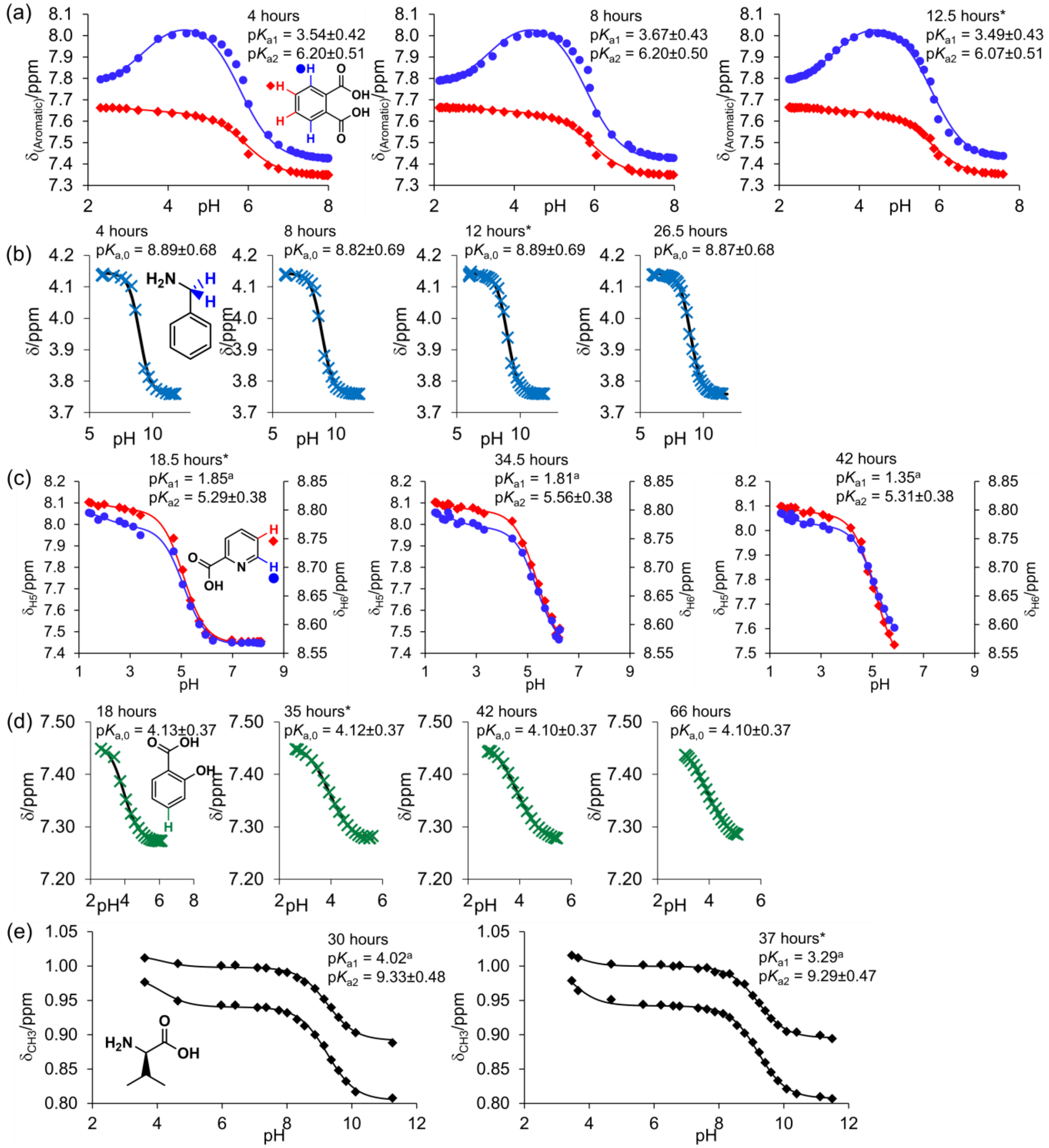

Figure S11. Experiments to determine p$K_{a,0}$ values of organic molecules at time indicated since layering a solution on top of 2,6-DHB: (a) phthalic acid in 30% CD_3CN/H_2O, (b) benzylamine in 30% CD_3CN/H_2O, (c) picolinic acid in 50% 1-propanol/H_2O, (d) salicylic acid in 50% 1-propanol/H_2O and (e) valine in 50% DMSO/H_2O. Experiments marked* are quoted in Table 2. [a]Approximate value from fitting to Equation 14. The two methyl resonances of valine overlap at very acidic pH < 3.4, preventing accurate measurement of a chemical shift.

S3. Extraction of A and B of Equation 3 in 1-propanol/H_2O and acetonitrile/H_2O from published mean activity coefficients of HCl

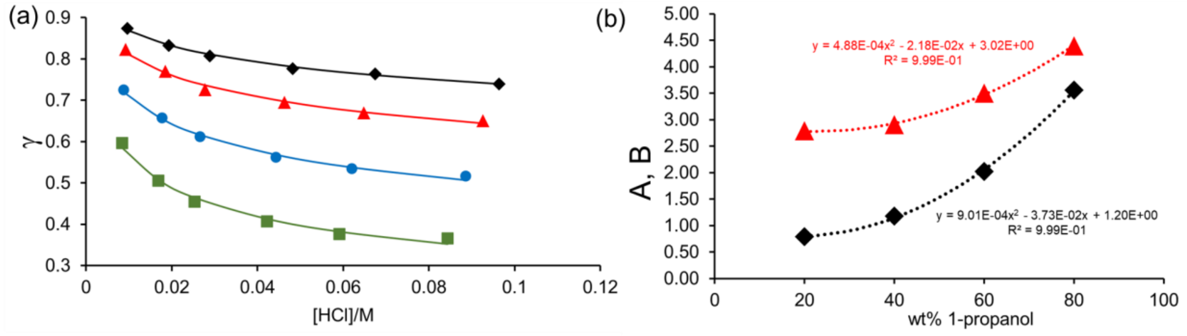

Figure S12. (a) Plot of mean activity coefficient of HCl, taken from Gentile *et al.*[105], versus molar concentration of HCl at 20 (black diamond), 40 (red triangle), 60 (blue circle) and 80 (green square) wt% 1-propanol/H_2O. Molarity of HCl in 1-propanol/H_2O mixtures was calculated from molality using density of 1-propanol/H_2O mixtures at 298K reported by Pang *et al.*[102] Solid lines are fits to Equation 3. (b) Plot of A (black diamond) and B (red triangle) obtained by fitting data of (a) to Equation 3. Lines for interpolations to 50% (v/v) 1-propanol/H_2O (44.6 wt%) are 2nd order polynomials, giving A = 1.32, B = 3.09. These values give values of γ of 0.724 and 0.615 at ionic strengths of 0.025 and 0.1 M, respectively, in agreement with the values of 0.724 and 0.624 presented by Jervis and Neelakantan.[106]

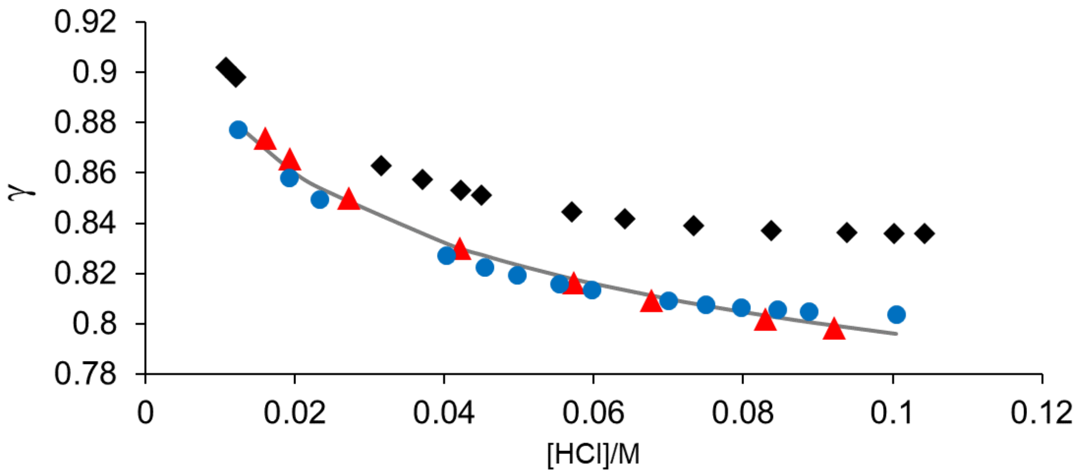

Figure S13. (a) Plot of mean activity coefficient of HCl, taken from Vega and Muñiz,[107] versus molar concentration of HCl at 10 (black diamond), 20 (red triangle) and 30 (blue circle) wt% acetonitrile/H_2O. Molarity of HCl in acetonitrile/H_2O mixtures was calculated from molality using density of acetonitrile/H_2O mixtures at 298K reported by Grande *et al.*[108] Grey line is fit of Equation 3 to activity coefficients at 20 and 30 wt% acetonitrile/H_2O at 298K taken from Vega and Muñiz,[107] which overlap. Activity coefficients at 30% (v/v) CD_3CN/H_2O are assumed equal to these values.

Parameters of Equation 3 for 50% (v/v) DMSO-d_6/H_2O are taken directly from Yang and Schulman,[109] setting a in Equation 5 of that work to 6.5 Å.

S4. Determination of pK_a values of analytes without correction for ionic strength

The fitting of the p$K_{a,0}$ values of 2,6-DHB, 1,2,4-triazole, the other indicators and the analytes was performed as described in the main text, but with A (Equation 3) set to zero.

50% 1-propanol/H_2O				50% DMSO/H_2O			30% CD_3CN/H_2O		
Indicator	pK_a	δ_H/ppm	δ_L/ppm	pK_a	δ_H/ppm	δ_L/ppm	pK_a	δ_H/ppm	δ_L/ppm
2,6-DHB[a]	1.39±0.43	6.4628	6.3124	0.91±0.21	6.4773	6.3362	1.43±0.41	6.4946	6.3684
1,2,4-triazole	1.95±0.53	9.2089	8.3164[b]	1.79±0.22	9.2026	8.3621[b]	2.37±0.43	9.1569	8.3278[b]
DMG[c]	-	-	-	2.66±0.22	4.0335	3.5755	2.49±0.44	3.9980	3.6370
Salicylic acid	-	-	-	3.35±0.25	7.5680	7.3772	-	-	-
Glycolic acid	4.22±0.53	4.1721	3.9171[d]	4.68±0.33	4.0993	3.7583[d]	4.31±0.47	4.1714	3.8644[d]
Acetic acid	5.23±0.55	2.0516	1.9160[d]	5.59±0.34	2.0293	1.8043[d]	5.34±0.53	2.0582	1.8485[d]
IM	6.10±0.61	8.7791	7.7155	-	-	-	6.68±0.58	8.6442	7.7254
2MI	7.04±0.64	2.6227	2.3574	6.72±0.37	2.5790	2.3181	7.62±0.61	2.5717	2.3233
4CN	8.29±0.65	7.5159	7.2840	8.15±0.40	7.6617	7.3510	8.30±0.63	7.6370	7.4052
DMG	9.25±0.67	2.9372[e]	2.2328[e]	9.31±0.50	3.5870[c,f]	2.8685[c]	9.69±0.71	3.6511[c,f]	2.9289[c]

Table S3. pK_a values of indicators determined with A = 0 (Equation 3). The datasets were the same as used for Table 1. [a]pK_a, δ_L and δ_H obtained in absence of 1,2,4-triazole using Equations 1-4, uncertainty obtained from experiment with 40 mM 1,2,4-triazole. [b]Average of δ_L determined with triazole (40 mM) and DSS alone, and in acidic range sample. [c]CH_2 resonance of DMG. [d]Average of acidic and basic range samples in absence of 2,6-DHB. [e]Methyl resonance of DMG. [f] δ_H differs from δ_L of the lower pK_a as we are approximating the protonation steps as separate due to the large difference in pK_a (Equation 14).

50% 1-propanol/H_2O				50% DMSO/H_2O				30% CD_3CN/H_2O			
Analyte	Indicator	pK_a	p$K_{a,0}$	Analyte	Indicator	pK_a	p$K_{a,0}$	Analyte	Indicator	pK_a	p$K_{a,0}$
Salicylic acid[a]	2,6-DHB, triazole, glycolate, acetate	3.87 ±0.54	4.07 (0.07 M)	Salicylic acid[a]	2,6-DHB, Triazole, DMG, glycolate, acetate	3.33 ±0.23	3.41 (0.05 M)	Salicylic acid[a]	2,6-DHB, Triazole, DMG, glycolate, acetate	3.66 ±0.44	3.74 (0.05 M)
Benzoic acid[b]	2,6-DHB, triazole, glycolate, acetate, 2MI	5.27 ±0.55	5.48 (0.09 M)	Benzoic acid	Triazole, glycolate, acetate, 2MI	5.11 ±0.33	5.20 (0.06 M)	Benzoic acid	Triazole, glycolate, acetate, 2MI	4.97 ±0.50	5.06 (0.06 M)
Picolinic acid[b]	2,6-DHB, triazole, glycolate, acetate, 2MI	1.40[c]<br>5.04 ±0.55	2.16[c] (0.11 M)<br>5.25 (0.09 M)	Bes[d]	Triazole, glycolate, acetate, 2MI	6.59 ±0.37	6.68 (0.07 M)	Phthalic acid	Triazole, glycolate, acetate, 2MI	3.34±0.44,<br>5.76 ±0.52	3.43 (0.06 M)<br>6.03 (0.07 M)
Acetylacetone	IM, 2MI, DMG	8.98 ±0.68[e]	9.15[e] (0.05 M)	4CN[f]	2MI, DMG	8.12 ±0.47	8.21 (0.06 M)	Quinine[g]	DMG, glycolate, acetate, IM, 2MI, DMG	3.76[c]<br>8.41 ±0.66	3.55 (0.12 M)<br>8.31 (0.11 M)
Pipecolic acid	Triazole, 2MI, DMG	2.46[c]<br>10.09 ±0.67	2.26[c] (0.07 M)<br>10.26 (0.05 M)	D-valine[f]	2MI, DMG	3.28[c]<br>9.15 ±0.50	3.19 (0.08 M)<br>9.24 (0.06 M)	Benzylamine[h]	DMG, 2MI, DMG	8.90 ±0.70	8.83 (0.03 M)

Table S4. pK_a values of organic analyte molecules uncorrected for ionic strength, determined using pK_a values and limiting chemical shifts of indicators in Table S3 by fitting to Equation 13 (γ = 1). p$K_{a,0}$ calculated from fitted pK_a using Equation 3 with values of A and B provided in main text, and ionic strength (brackets) when pH closest to pK_a of analyte. [a]Determined from acidic-range dataset. [b]8-9 mg 2,6-DHB. [c]Approximate value from fitting to Equation 14. [d]N,N-Bis(2-hydroxyethyl)-2-aminoethanesulfonate. Sample also contained DMG sodium salt (2 mM), tricine (2 mM), formate (4 mM), *tert*-butylamine (10 mM), which were unsuitable for use as indicators. 5-6 mg 2,6-DHB used. [e]Value corrected for enol-ketone tautomerization. [f]Sample also contained NaOH (10 mM), D-valine Na salt (2 mM) and 4CN sodium salt (20 mM). [g]Basic-range dataset. [h]Sample contained NaOH (10 mM) in addition to indicators. 3-4 mg 2,6-DHB used.

S5. Interpolation of pK_a values from published data

The solvent mixtures used in this study are equivalent in terms of the mole fraction of the organic solvent, X, to the non-deuterated solvent mixtures of 44.6 wt% 1-propanol/H_2O (X = 0.195), 52.4 wt% DMSO/H_2O (X = 0.202) and 25.1 wt% acetonitrile/H_2O (X = 0.128). These wt% and mole fractions were used to interpolate literature pK_a data reported in non-deuterated solvent mixtures. Published data was fitted using linear fits, second or third order polynomials as judged from the data. Example interpolation curves are shown below for 2,6-DHB and salicylic acid in 1-propanol/H_2O mixtures. All literature pK_a data used in this work was reported at 298 K. Where reported pK_a values were not thermodynamic, the thermodynamic pK_a was calculated from the reported ionic strength using Equation 3 and these corrected values are reported as literature values in Table 2.

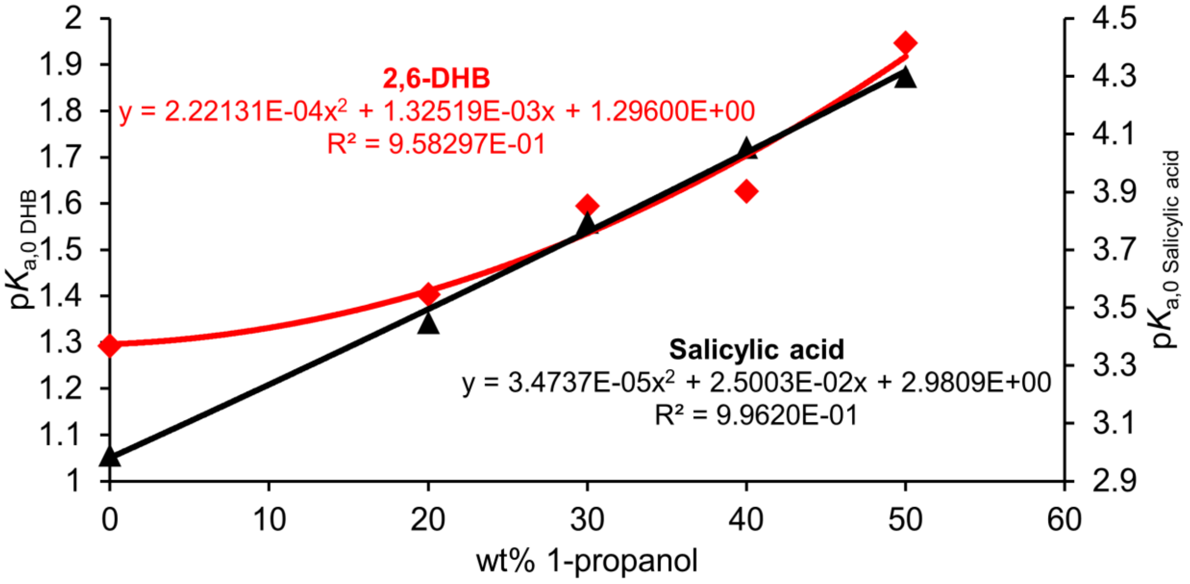

Figure S14. Plot of p$K_{a,0}$ of 2,6-DHB (red diamond) and salicylic acid (black triangle) versus wt% of 1-propanol in 1-propanol/H_2O mixtures from Papadopoulos and Avranas.[110] Fits to second order polynomials (solid lines) used to interpolate value at 50% (v/v) 1-propanol/H_2O (44.6 wt%).

S6. Extraction of p$K_{a,0}$ of 2,6-DHB *via* observation of resonance of 4-position

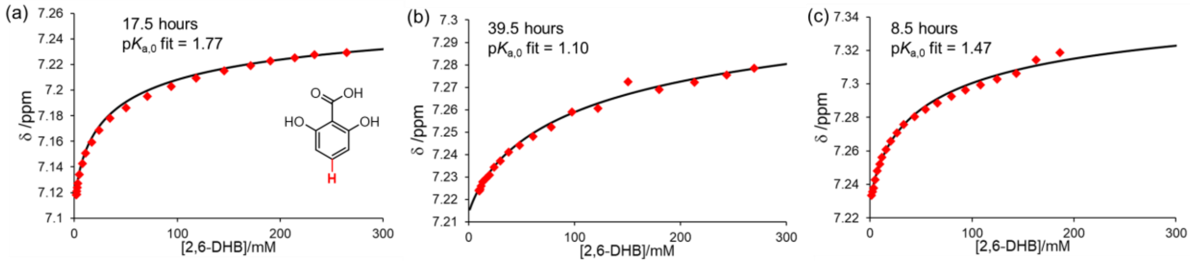

Figure S15. Plot of ^1H chemical shift of 4-position of 2,6-DHB versus concentration in experiment to determine p$K_{a,0}$ of 2,6-DHB in absence of base. (a) 50% 1-propanol/H_2O, (b) 50% DMSO/H_2O and (c) 30% CD_3CN/H_2O. Solid lines are fits to Equations 1 – 4. The fitted values of p$K_{a,0}$ are within experimental uncertainty (see p$K^*_{a,0\ DHB}$ in main text) of the values determined by fitting the 3,5-position (Table 1).

S7. Uncertainty analysis in determination of pK_a and pH

S7.1 Calculation of uncertainty in p$K_{a,0}$ of 2,6-DHB and 1,2,4-triazole

All uncertainty calculations are performed using the spreadsheets accompanying this work. A propagation of uncertainty analysis of Equation 9, ignoring γ, yields the overall uncertainty in pH_i for an indicator (Equation S5):[111]

$$\Delta_{\mathrm{pH}_i} = \sqrt{\Delta_{\mathrm{p}K_{\mathrm{a,0}}}{}^2 + \left(\frac{\Delta_{\delta_\mathrm{H}}}{2.303(\delta_\mathrm{obs} - \delta_\mathrm{H})}\right)^2 + \left(\frac{\Delta_{\delta_\mathrm{L}}}{2.303(\delta_\mathrm{L} - \delta_\mathrm{obs})}\right)^2 + \left(\frac{\Delta_{\delta_\mathrm{obs}}(\delta_\mathrm{L} - \delta_\mathrm{H})}{2.303(\delta_\mathrm{obs} - \delta_\mathrm{H})(\delta_\mathrm{L} - \delta_\mathrm{obs})}\right)^2} \quad \text{S5}$$

where Δ denotes the uncertainty in the subscripted variable. $\Delta_{\delta_\mathrm{H}}$ and $\Delta_{\delta_\mathrm{L}}$ are taken as 0.005 ppm, $\Delta_{\delta_\mathrm{obs}}$ as 0.001 ppm in this work.

In the determination of the p$K_{a,0}$ of triazole using the values of p$K_{a,0\ DHB}$, δ_H and δ_L of 2,6-DHB obtained in the absence of triazole, a provisional uncertainty in the pH determined from the ^{1}H chemical shift of 2,6-DHB (Equation 5) is calculated from Equation S5, with $\Delta_{\mathrm{pK_{a,0\,DHB}}}$ calculated using the following procedure: The difference between the experimental and fitted chemical shift of 2,6-DHB in the absence of triazole (Figure 1a) averaged over every point along the 2,6-DHB gradient, Δ_{av}, is used to calculate a maximum and minimum value of f at each point using Equation S6 and S7:

$$\mathrm{f_{max}} = \frac{\delta_\mathrm{H} - (\delta_\mathrm{DHB} - \Delta_\mathrm{av})}{\delta_\mathrm{H} - \delta_\mathrm{L}} \quad \text{S6}$$

$$\mathrm{f_{min}} = \frac{\delta_\mathrm{H} - (\delta_\mathrm{DHB} + \Delta_\mathrm{av})}{\delta_\mathrm{H} - \delta_\mathrm{L}} \quad \text{S7}$$

Maximum and minimum values of p$K_{a,0\ DHB}$ are calculated at each datapoint along the sample using Equations S8 and S9:

$$\mathrm{p}K_{\mathrm{a,0\ DHB\ max}} = -\log_{10}\left(\frac{\gamma^2 \mathrm{f_{min}^2 C}}{1 - \mathrm{f_{min}}}\right) \quad \text{S8}$$

$$\mathrm{p}K_{\mathrm{a,0\ DHB\ min}} = -\log_{10}\left(\frac{\gamma^2 \mathrm{f_{max}^2 C}}{1 - \mathrm{f_{max}}}\right) \quad \text{S9}$$

$\Delta_{\mathrm{pK_{a,0\,DHB}}}$ (Equation S5) is taken as half the difference between the average p$K_{a,0\ max}$ and the average p$K_{a,0\ min}$ over all points in the dataset recorded in the absence of triazole. This value is used to calculate a provisional uncertainty in the pH in the experiment with triazole using Equation S5 (with $\Delta_{\delta_\mathrm{obs}}$ and $\Delta_{\delta_\mathrm{H}}$ as 0.001 ppm, $\Delta_{\delta_\mathrm{L}}$ as 0.005 ppm) which is used to exclude fitting points for Equation 7 where this uncertainty exceeds 0.1 units. $\Delta_{\mathrm{pK_{a,0\,DHB}}}$was obtained as 0.08, 0.03 and 0.03 for 50% 1-propanol/H_2O, 50% DMSO/H_2O and 30% CD_3CN/H_2O, respectively.

Having fitted the ^{1}H chemical shift of 2,6-DHB to Equations 2 and 8, and the chemical shift of triazole to Equation 7, the overall uncertainty in pH is calculated from Equation S5, taking $\Delta_{\mathrm{pK_{a,0\,DHB}}}$, $\Delta_{\delta_\mathrm{H}}$ and $\Delta_{\delta_\mathrm{L}}$ as the difference in the values of p$K_{a,0}$, δ_H and δ_L of 2,6-DHB fitted in the presence (p$K^*_{a,0\ DHB}$) and absence (p$K_{a,0\ DHB}$) of triazole. The uncertainty in p$K_{a,0}$ of triazole is taken as the average uncertainty in pH, thus calculated, over all experimental points used to fit Equation 7.

S7.2 Calculation of uncertainty in p$K_{a,0}$ of other indicators

Known indicators with chemical shifts within Δ_{δ_H} or Δ_{δ_L} (0.005 ppm) of δ_H or δ_L were excluded from the calculation. For indicators for which pH_i could be calculated, the uncertainty in pH_i was calculated using Equation S5, along with an uncertainty arising from chemical shift alone, Δ_{pHi}', from Equation S5 with $\Delta_{pKa,0}$ set to zero. For the known indicators, if Δ_{pHi}' was less than 0.05 (0.1 for the calibration of imidazole in 50% 1-propanol/H_2O), S_i was calculated using Equation 11, and the pH of the row calculated using Equation 12. Δ_{pHi}' was also calculated for the new indicator being fitted. If Δ_{pHi}' for the new indicator was less than 0.4 (0.8 for 4CN in 50% 1-propanol/H_2O) following fitting of its chemical shift to Equation 13, it was included in the calculation of the pH of the row using Equation 12. The uncertainty in p$K_{a,0}$ for the new indicator was taken as the difference between the pH calculated using Equation 12 with only the known indicators and pH_i of the new indicator, for all rows where pH_i could be calculated, plus the highest $\Delta_{pKa,0}$ of the known indicators. The uncertainty in p$K_{a,0}$ of the indicators thus increases as p$K_{a,0}$ rises (Table 1).

S7.3 Calculation of uncertainty in p$K_{a,0}$ of analyte molecules

Indicators with chemical shifts within Δ_{δ_H} or Δ_{δ_L} (0.005 ppm) of δ_H or δ_L were excluded from the calculation of pH. The uncertainty in the pH of each row of the CSI dataset was calculated as the sensitivity-weighted average of the uncertainties in the pH_i reported by all indicators, analogous to Equation 12 (Equation S10):

$$\Delta_{pH} = \frac{\sum_{i=1}^{n} S_i \Delta_{pH_i}}{\sum_{i=1}^{n} S_i} \qquad \text{S10}$$

The uncertainty in the fitted p$K_{a,0}$ of the analyte was taken as the value of Δ_{pH} calculated for the row of the CSI dataset with pH closest to the value of p$K_{a,0\ analyte}$- $\Delta z^2 \log_{10}(\gamma)$, where $\log_{10}(\gamma)$ was calculated using Equations 3 and 10. The calculation of ionic strength of a row ignores the charge state of the analyte molecule. The same procedure was used for the p$K_{a,0}$ values of diprotic compounds (Figure 4).

S7.4 Derivation of Equations 8, 13 and 14

S7.4.1 Derivation of Equation 8

q (Equation 8) is the equilibrium constant for the reaction of a base (1,2,4-triazole) with an acid (2,6-DHB):

$$q = \frac{[DHB^-][TH^+]}{[DHB][T]} \qquad \text{S11}$$

where $[DHB^-]$ and [DHB] are the equilibrium concentrations of deprotonated and neutral 2,6-DHB, respectively. [T] and $[TH^+]$ are the equilibrium concentrations of neutral and protonated triazole, respectively. These equilibrium concentrations can be expressed in terms of $[DHB^-]$, $[H^+]$ and the total concentrations of 2,6-DHB (C) and triazole (T):

$$[TH^+] = [DHB^-] - [H^+] \qquad \text{S12}$$

$$[T] = T - [DHB^-] + [H^+] \quad S13$$

$$[DHB] = C - [DHB^-] \quad S14$$

Equation S11 can be rewritten as:

$$q = \frac{[DHB^-]([DHB^-] - [H^+])}{(C - [DHB^-])(T - [DHB^-] + [H^+])} \quad S15$$

$[DHB^-]$ can then be obtained from the quadratic formula, with f obtained as $[DHB^-]/C$ to give Equation 8. Assuming the activity coefficients of protonated 2,6-DHB and neutral triazole to be 1, and the activity coefficients of deprotonated 2,6-DHB and protonated triazole to be equal and given by Equation 3, q (concentration-based) is obtained from the thermodynamic pK_a values of triazole and 2,6-DHB as: $q = \gamma^{-2} 10^{pK_{a,0\,triazole} - pK^*_{a,0\,DHB}}$.

S7.4.2 Derivation of Equation 13 and 14

Assuming fast exchange on the chemical shift timescale between the protonated (HA) and non-protonated (A) states, the chemical shift of an observed species can be related to the pH of the solution *via* the Henderson-Hasselbalch equation:[112]

$$\delta_{obs} = \frac{\delta_H + \delta_L 10^{pH - pK_a}}{1 + 10^{pH - pK_a}} \quad S16$$

where the pK_a is of the 'mixed' type if pH is on an activity scale:[113]

$$K_{a\,mixed} = \frac{\gamma[H^+][A]}{[HA]} = \gamma[H^+]\left(\frac{\delta_H - \delta_{obs}}{\delta_{obs} - \delta_L}\right) \quad S17$$

Making the approximation that the activity coefficient only depends on charge, the activity coefficient of an ion of charge z (γ_z) is obtained from Equation 3 as:

$$\log_{10}(\gamma_Z) = -Az^2 \frac{\sqrt{I}}{1 + B\sqrt{I}} = z^2 \log_{10}(\gamma) \quad S18$$

The thermodynamic dissociation constant depends on the charge of the molecule in its protonated (z_H) and deprotonated (z_L) states:

$$K_{a,0} = \frac{\gamma[H^+]\gamma_{Z_L}[A]}{\gamma_{Z_H}[HA]} = \frac{\gamma[H^+][A]}{[HA]} 10^{-(z_H^2 - z_L^2)\log_{10}(\gamma)} \quad S19$$

p$K_{a\,mixed}$ and p$K_{a,0}$ are thus interconverted through Equation S20, and combined with Equation S16 to yield Equation 13.

$$pK_{a\ mixed} = pK_{a,0} - (z_H^2 - z_L^2)\log_{10}(\gamma) = pK_{a,0} - \Delta z^2 \log_{10}(\gamma) \quad S20$$

For compounds with two dissociation steps, δ_{obs} is a weighted average of the fully protonated (H_2A), monoprotonated (HA) and deprotonated (A) states:

$$\delta_{obs} = \frac{\delta_L[A] + \delta_{HL}[HA] + \delta_H[H_2A]}{[A] + [HA] + [H_2A]} \quad S21$$

$$= \frac{\delta_L + \delta_{HL}\frac{\gamma[H^+]}{K_{a2}} + \delta_H\frac{\gamma^2[H^+]^2}{K_{a2}K_{a1}}}{1 + \frac{\gamma[H^+]}{K_{a2}} + \frac{\gamma^2[H^+]^2}{K_{a2}K_{a1}}}$$

where K_a values are mixed (Equation S17). As $pH = -\log_{10}(\gamma[H^+])$, Equation S21 can be written as:

$$\delta_{obs} = \frac{\delta_L + \delta_{HL}10^{pK_{a2\ mixed}-pH} + \delta_H 10^{pK_{a2\ mixed}+pK_{a1\ mixed}-2pH}}{1 + 10^{pK_{a2\ mixed}-pH} + 10^{pK_{a2\ mixed}+pK_{a1\ mixed}-2pH}} \quad S22$$

Combining Equations S20 and S22 yields Equation 14.

S8. Example ^{1}H spectra from CSI datasets

Rows 1-6 and 28-32 (32 point datasets) and rows 1-13 and 58-64 (64 point datasets) have been deleted from the plots below as they are not used in the analysis (Experimental Section).

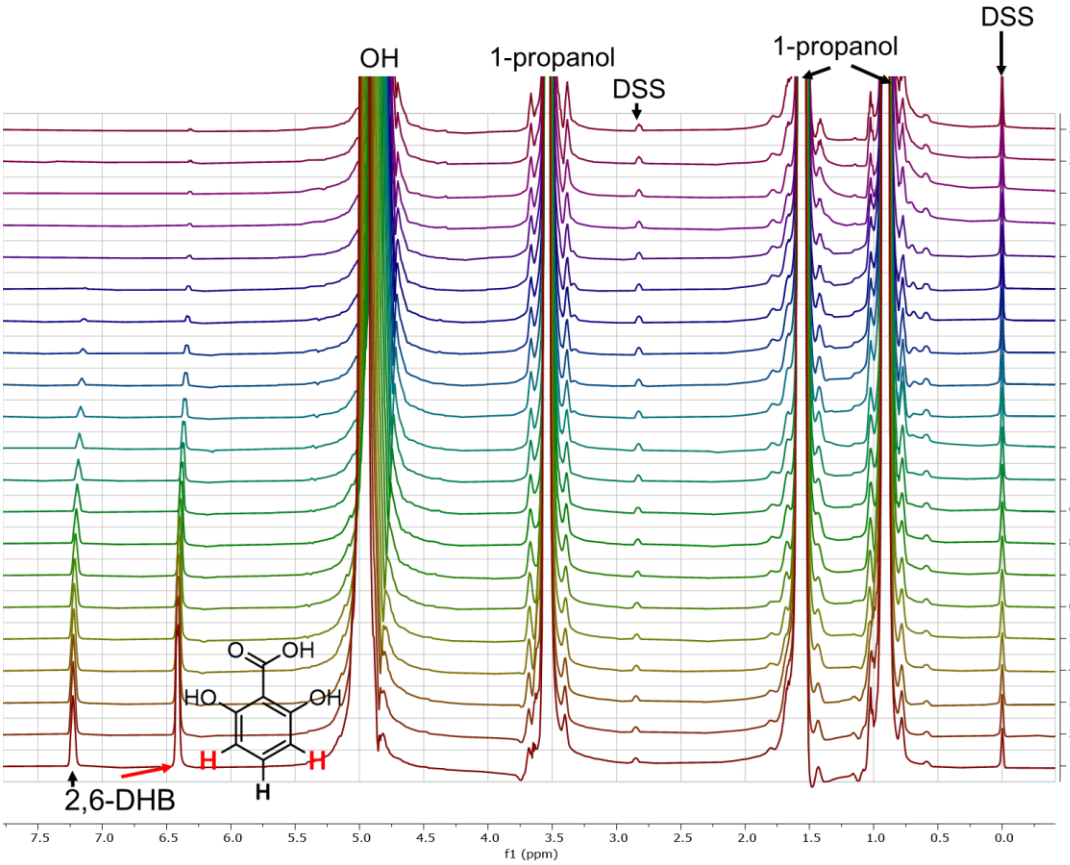

Figure S16. ^{1}H CSI dataset to determine pK_a of 2,6-DHB in 50% 1-propanol/H_2O (Figure 1a).

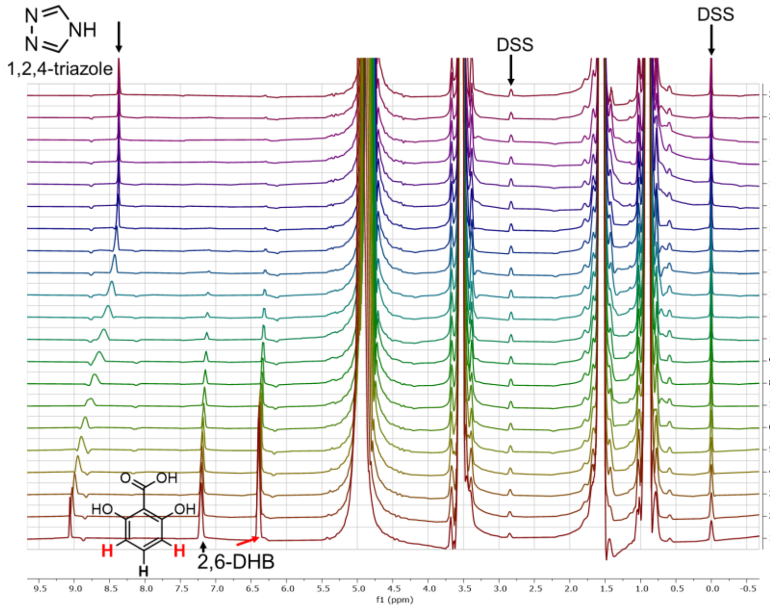

Figure S17. ^{1}H CSI dataset to determine pK_a of 2,6-DHB and 1,2,4-triazole in 50% 1-propanol/H_2O (Figure 1b).

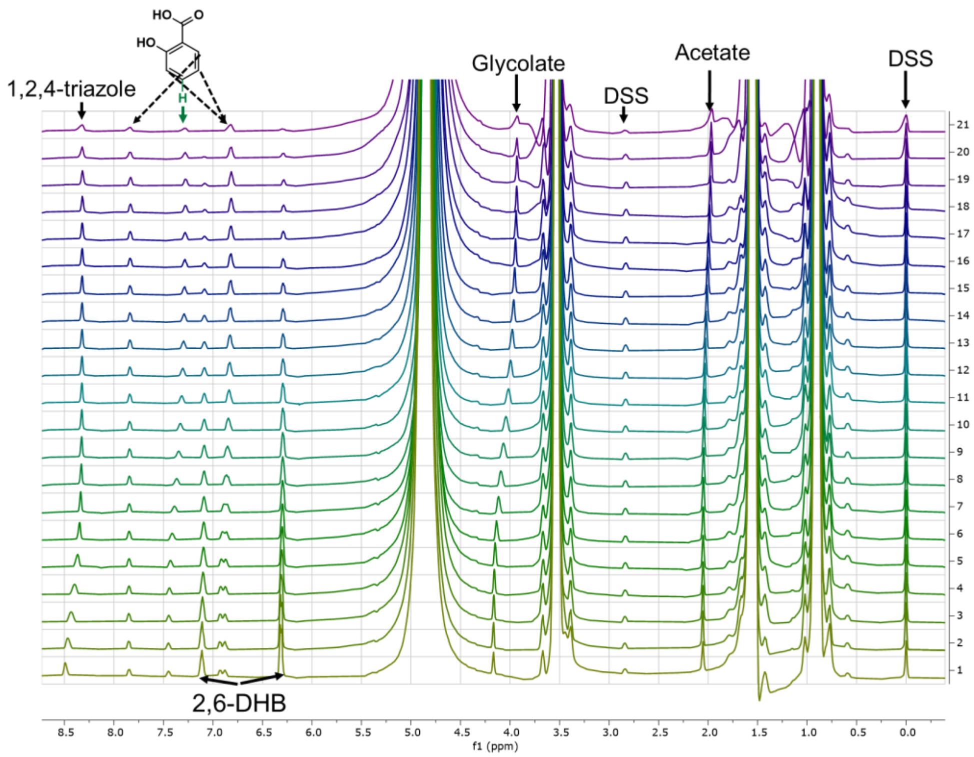

Figure S18. [1]H CSI dataset to determine pK_a of glycolate, acetate and salicylate in 50% 1-propanol/H_2O (Table 1). Observed shift of salicylic acid (Figure 3) indicated with green arrow.

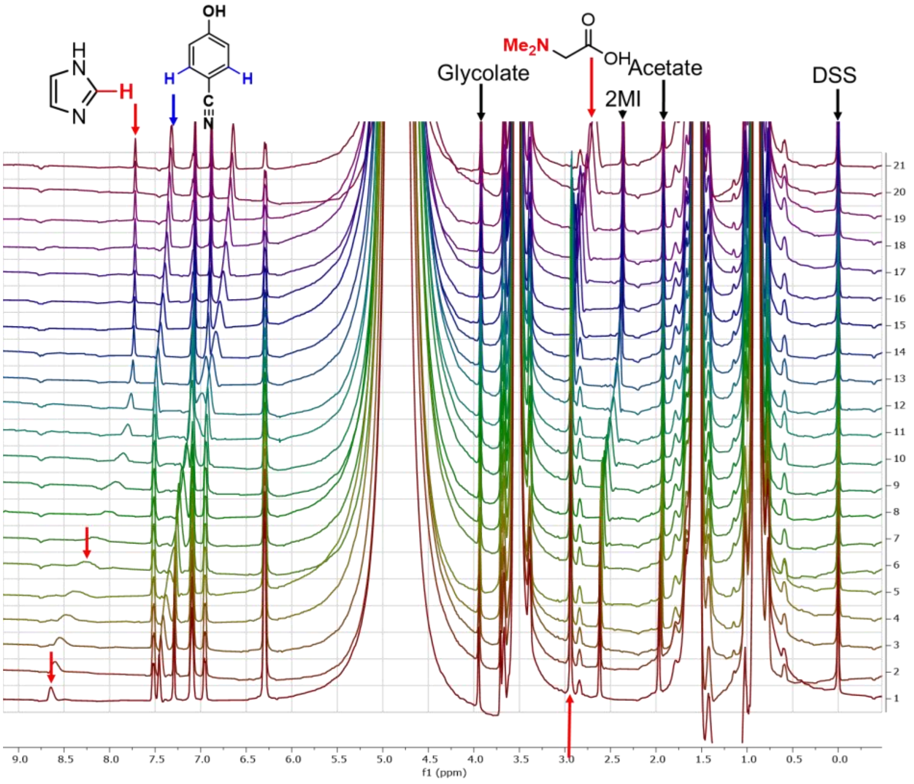

Figure S19. [1]H CSI dataset to determine pK_a of imidazole, 2MI, 4CN and DMG in 50% 1-propanol/H_2O (Table 1).

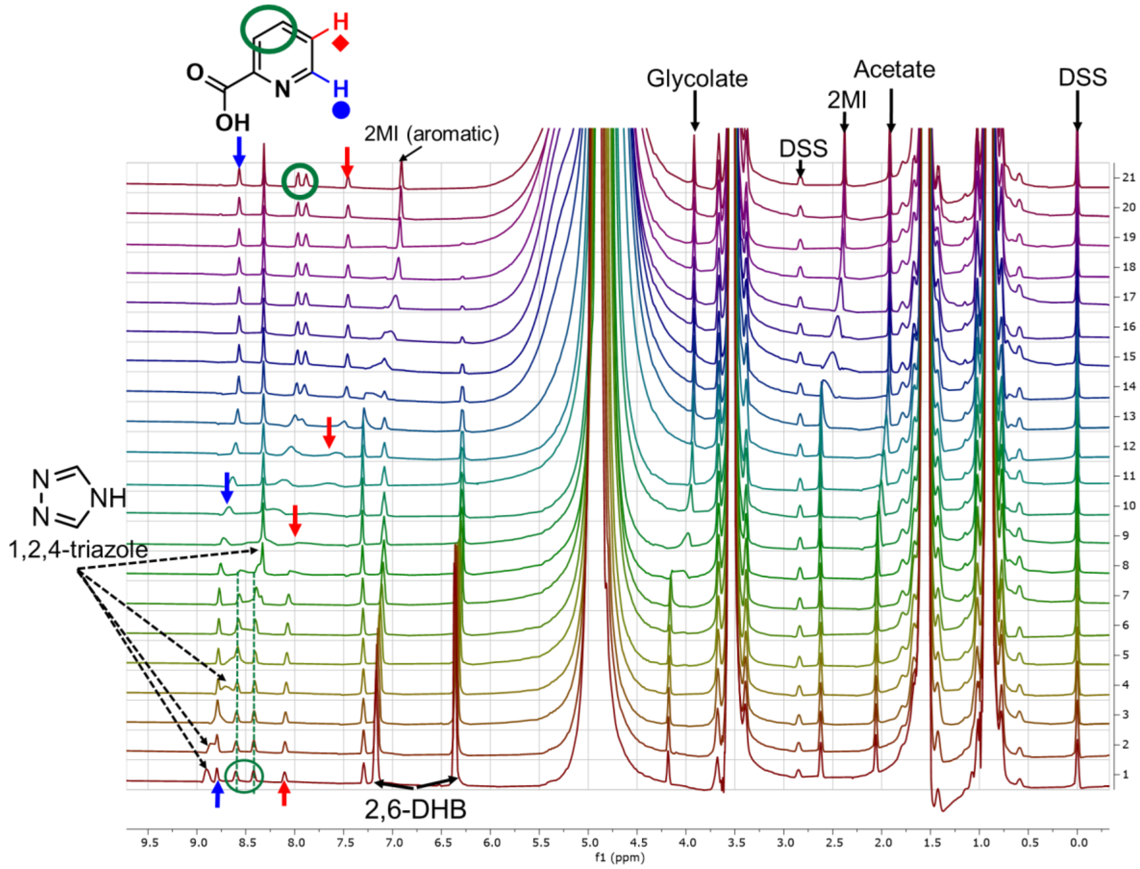

Figure S20. ^{1}H CSI dataset for determination of pK_a values of picolinic acid in 50% 1-propanol/H_2O (Table 2 and Figure 4). ^{1}H chemical shifts of 3- and 4-positions of picolinic acid (green circle) overlap so cannot be used to extract a pK_a. However, these resonances do not move significantly more than the 5- and 6-positions when the pH falls below 4 (green dashed lines) so would not provide a more accurate estimate of pK_{a1}.

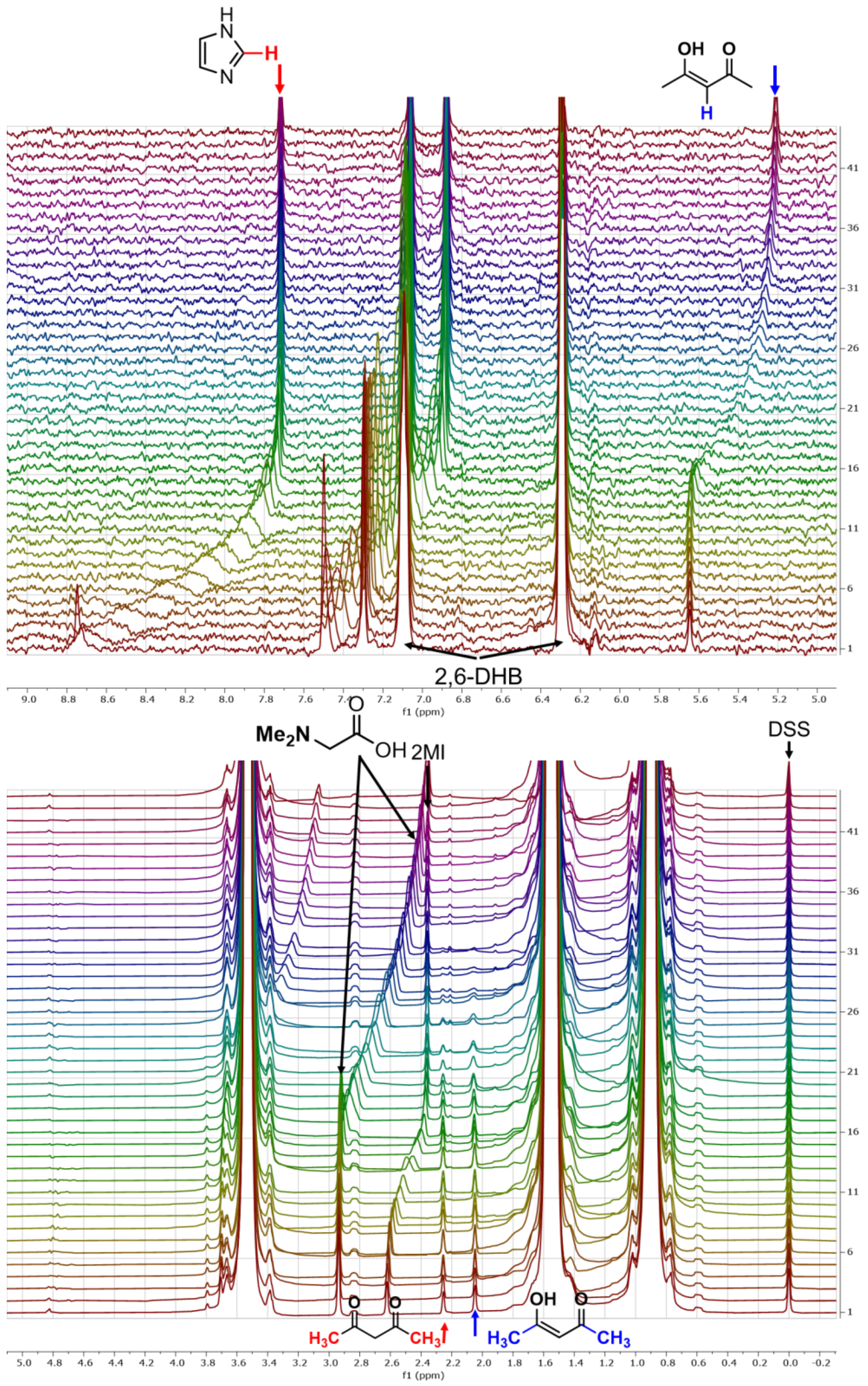

Figure S21. ^{1}H CSI dataset for determination of pK_a value of acetylacetone in 50% 1-propanol/H_2O. Methyl resonances of enol and ketone tautomers are indicated.[114] The ketone-enol tautomerisation is slow on the ^{1}H NMR chemical shift timescale so separate methyl signals are observed. However, the deprotonation of the enol tautomer is fast on the NMR timescale so a single pH-dependent chemical shift is observed for the proton on the unsaturated carbon (Figure 3). By fitting the ^{1}H chemical shift of this proton to Equation 13, we obtain the p$K_{a,0}$ of the enol (8.94 ± 0.50).

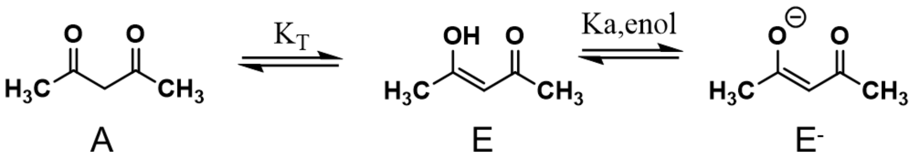

The enol-ketone tautomerisation constant (K_T) is given by [E]/[A] and can be measured directly from the lower rows of the CSI dataset (pH < 7, where deprotonated form is absent) by integrating the methyl resonances of the two tautomers.[114] We write the overall apparent K_a, K_{app}, of acetylacetone (as determined potentiometrically by Gentile *et al*.[115]) as:

$$K_{app} = \frac{[H^+][E^-]}{[A]+[E]} = \frac{[H^+][E^-]}{[E]\left(1+\frac{1}{K_T}\right)} = \frac{K_{a,enol}}{1+\frac{1}{K_T}} = jK_{a,enol} \quad \text{S23}$$

where j is the measured fraction of compound in the enol tautomer in the lower rows of the dataset (when pH < 7), determined as 0.52 by integration of the methyl resonances of the two tautomers.

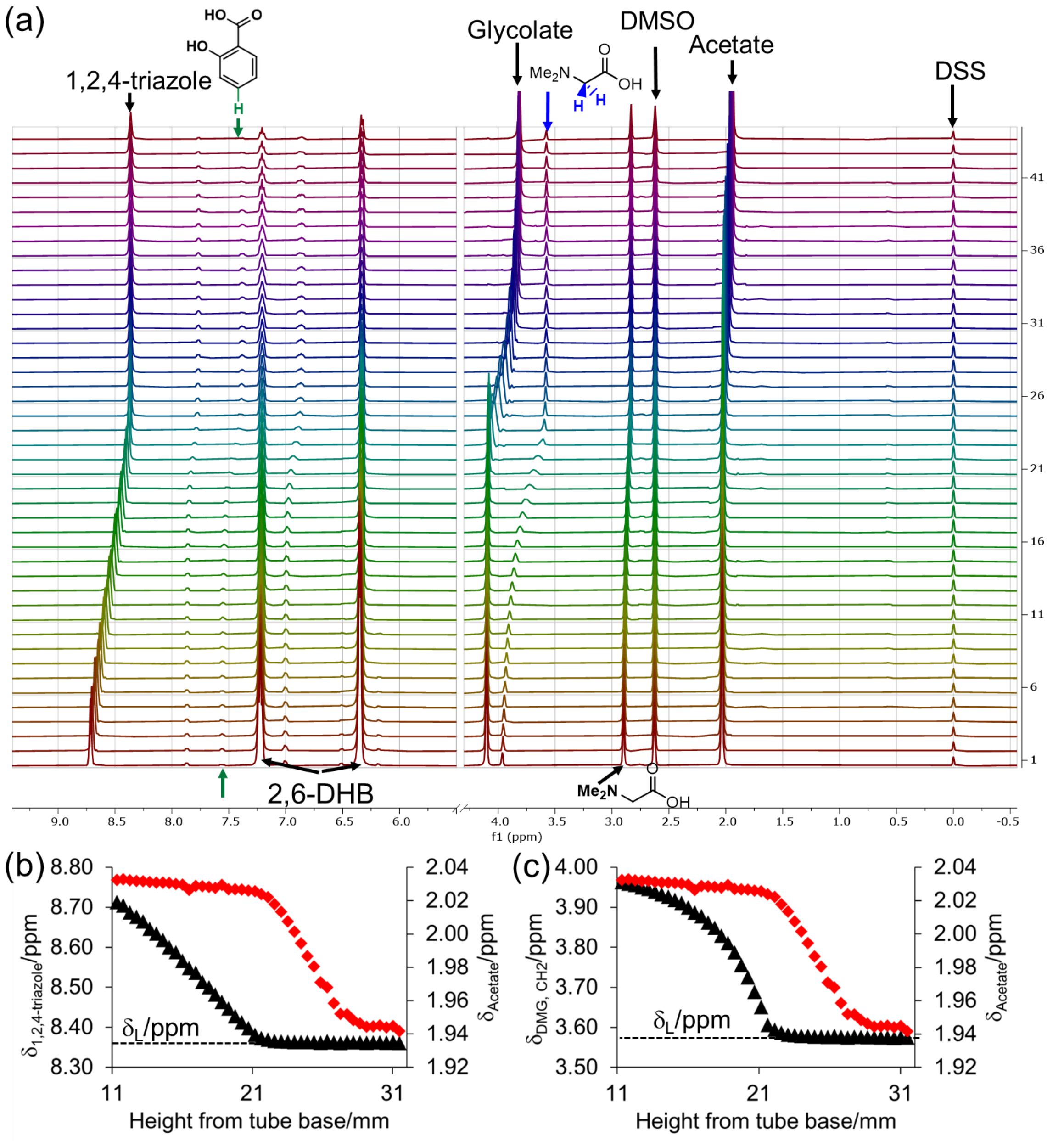

Figure S22. (a) ^{1}H CSI dataset for determination of pK_a values of DMG, glycolate, acetate and salicylate (acidic-range) in 50% DMSO/H_2O (Figure 2). (b) Plot of ^{1}H chemical shift of 1,2,4-triazole (black triangle) and acetate (red diamond) versus height from base of the NMR tube. Above 21 mm, the ^{1}H chemical shift of triazole does not change with position, whereas the chemical shift of acetate continues to fall as the in pH rises towards the top of the sample, indicating that triazole in its essentially fully neutral form 30 mm from the tube base. (c) Analogous plot for DMG (CH_2), showing that DMG is essentially fully zwitterionic 30 mm from the tube base, allowing δ_L to taken as the chemical shift of DMG at this position.

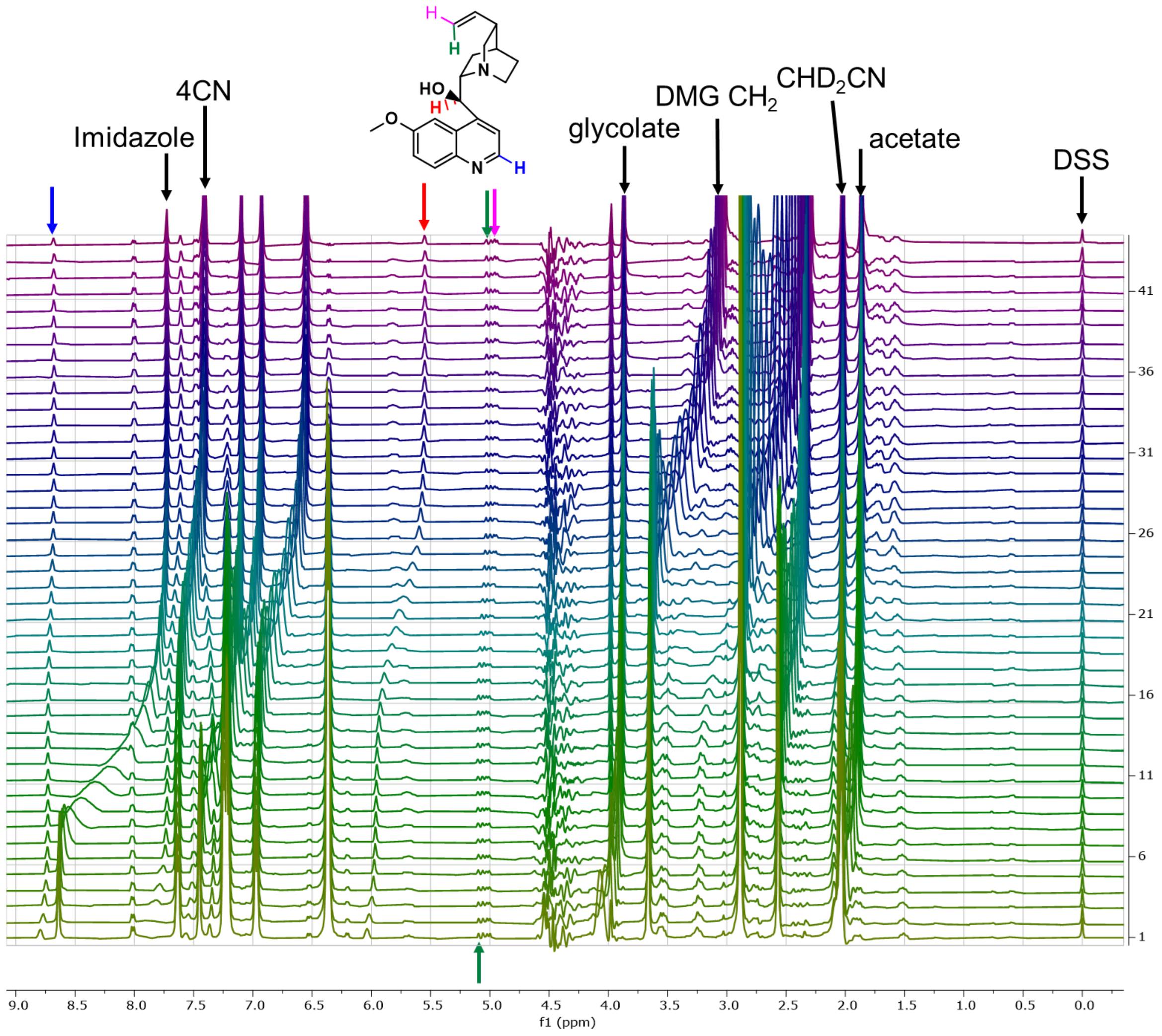

Figure S23. ^{1}H CSI dataset to determine pK_a of imidazole, 2MI, 4CN, DMG as indicators, and quinine as analyte in 30% CD_3CN/H_2O (Tables 1, 2 and Figure 4).

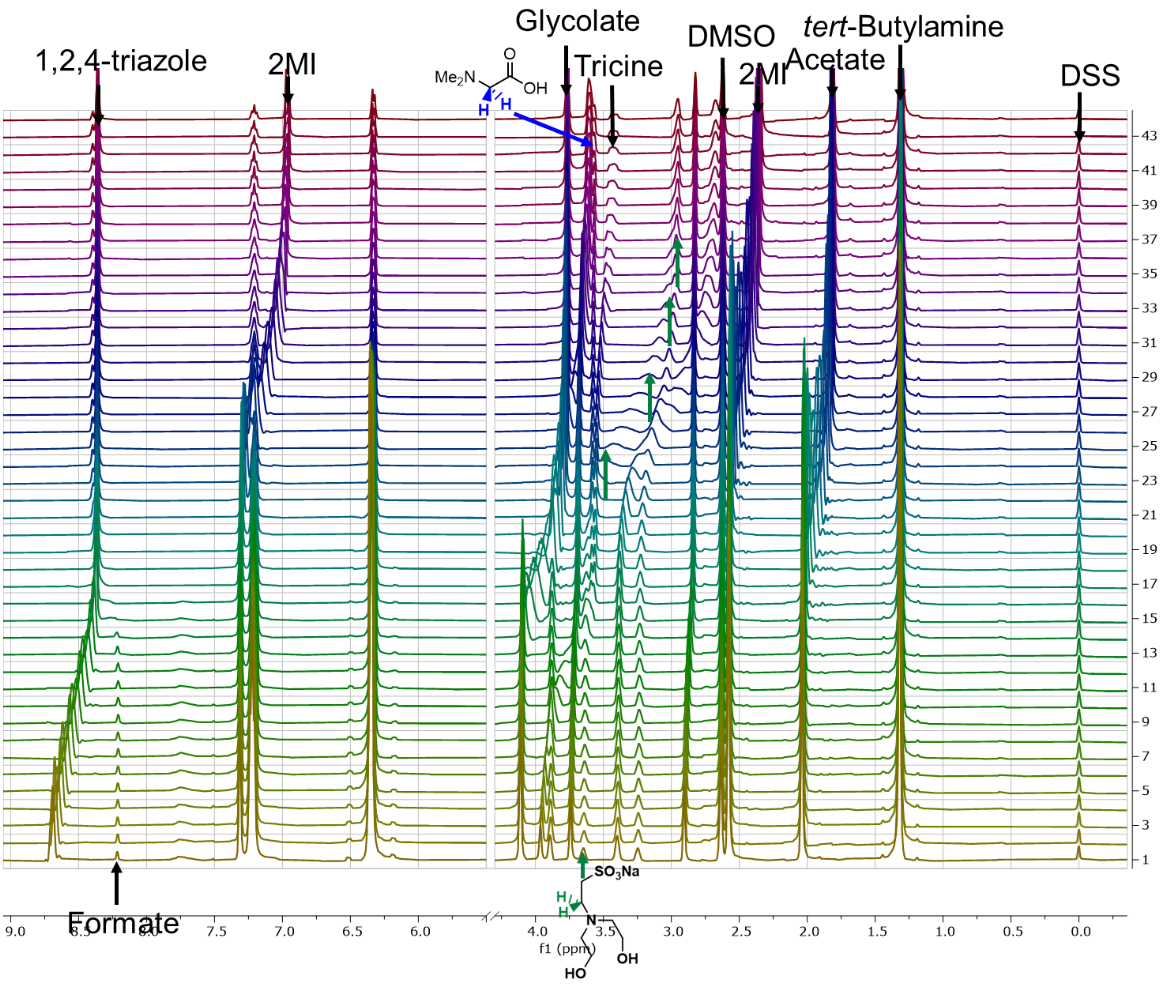

Figure S24. [1]H CSI dataset to determine pK_a of Bes in 50% DMSO/H_2O (Table 2 and Figure 3). Observed ^{1}H resonance of Bes is indicated with a green arrow. Other resonances could not be used due to excessive overlap. Sample also contained tricine (2 mM), formate (4 mM), *tert*-butylamine (10 mM), which were found unsuitable or unnecessary for use as indicators due to similar pK_a values to DMG (*tert*-butylamine) or due to overlap with other indicators (formate, tricine).

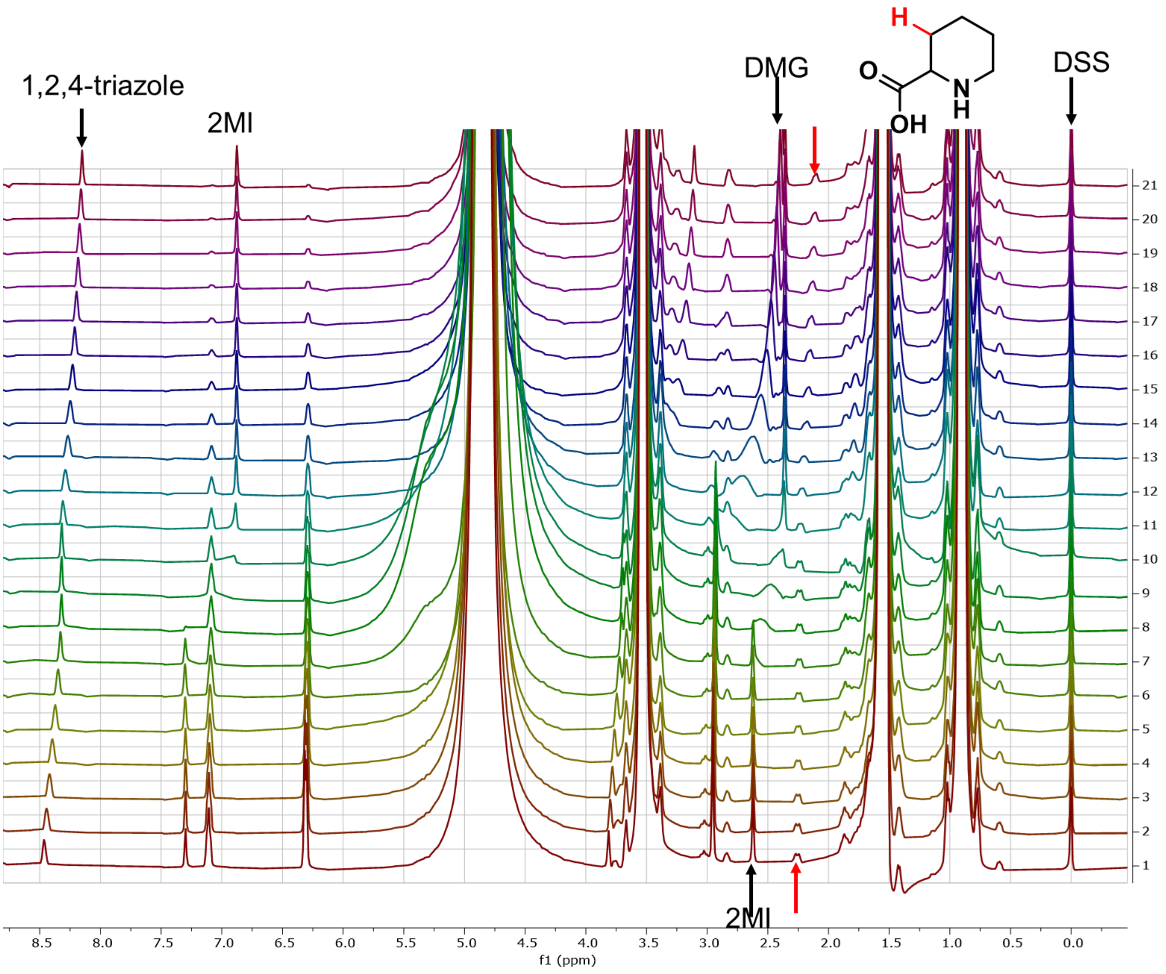

Figure S25. ^{1}H CSI dataset to determine pK_a of pipecolic acid in 50% 1-propanol/H_2O (Table 2 and Figure 4). Observed ^{1}H resonance of pipecolic acid is indicated with a red arrow. Other resonances are unsuitable for observation due to overlap with the resonances of 1-propanol, or other indicators.

S9. Calibration plots of indicators in 50% 1-propanol/H_2O and 30% CD_3CN/H_2O

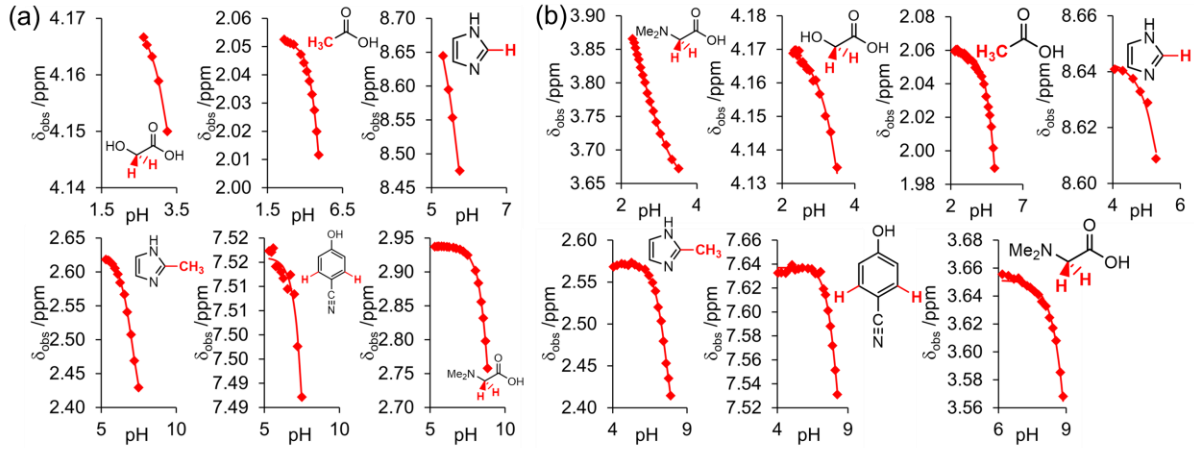

Figure S26. Plot of ^{1}H chemical shifts of indicators (red diamond) used to determine δ_H, δ_L and p$K_{a,0}$ in 50% 1-propanol/H_2O (a) and 30% CD_3CN/H_2O (b). Solid lines are fits to Equation 13.

References

100. Crank, J., *The Mathematics of Diffusion, 2nd ed.* Clarendon Press: Oxford 1975.

101. Wallace, M.; Adams, D. J.; Iggo, J. A., Titrations without the Additions: The Efficient Determination of p K a Values Using NMR Imaging Techniques. *Anal. Chem.* **2018,** *90* (6), 4160-4166.

102. Pang, F. M.; Seng, C. E.; Teng, T. T.; Ibrahim, M. H., Densities and viscosities of aqueous solutions of 1-propanol and 2-propanol at temperatures from 293.15 K to 333.15 K. *J. Mol. Liq.* **2007,** *136* (1-2), 71-78.

103. Carmen Grande, M. d.; Juliá, J. A.; García, M.; Marschoff, C. M., On the density and viscosity of (water + dimethylsulphoxide) binary mixtures. *J. Chem. Thermodyn.* **2007,** *39* (7), 1049-1056.

104. Cunningham, G. P.; Vidulich, G. A.; Kay, R. L., Several Properties Of Acetonitrile-Water, Acetonitrile-Methanol, and Ethylene Carbonate-Water Systems. *J. Chem. Eng. Data* **1967,** *12* (3), 336-337.

105. Gentile, P. S.; Eberle, L.; Cefola, M.; Celiano, A. V., Electromotive Force Measurements of the Cell-Pt/H2: HCl(m): Agcl/Ag-, in 1-Propanol-Water Mixtures. *J. Chem. Eng. Data* **1963,** *8* (3), 420-421.

106. Jervis, R. E.; Neelakantan, K., On the measurement of low acidity (pHc) in acetone-water and 1-propanol-water. *J. Inorg. Nucl. Chem.* **1975,** *37* (2), 531-535.

107. Vega, C. A.; Muñiz, M. D. L. A., Standard potential of the (Ag+AgCl) electrode in (acetonitrile+water). *J. Chem. Thermodyn.* **1985,** *17* (12), 1163-1170.

108. del Carmen Grande, M.; Juliá, J. A.; Barrero, C. R.; Marschoff, C. M.; Bianchi, H. L., The (water + acetonitrile) mixture revisited: A new approach for calculating partial molar volumes. *J. Chem. Thermodyn.* **2006,** *38* (6), 760-768.

109. Yang, R.; Schulman, S. G., An operational pH in aqueous dimethylsulfoxide based upon the acidity dependence of the rate of a simple ionic recombination reaction in the lowest excited singlet state. *Talanta* **2003,** *60* (2-3), 535-542.

110. Papadopoulos, N.; Avranas, A., Dissociation of salicylic acid, 2,4-, 2,5-and 2,6-dihydroxybenzoic acids in 1-propanol-water mixtures at 25°C. *J. Solution Chem.* **1991,** *20* (3), 293-300.
111. Wallace, M.; Lam, K.; Kuraite, A.; Khimyak, Y. Z., Rapid Determination of the Acidity, Alkalinity and Carboxyl Content of Aqueous Samples by 1H NMR with Minimal Sample Quantity. *Anal. Chem.* **2020,** *92* (19), 12789-12794.
112. Ackerman, J. J. H.; Soto, G. E.; Spees, W. M.; Zhu, Z.; Evelhoch, J. L., The NMR chemical shift pH measurement revisited: Analysis of error and modeling of a pH dependent reference. *Magn. Reson. Med.* **1996,** *36* (5), 674-683.
113. Irving, H. M.; Miles, M. G.; Pettit, L. D., A study of some problems in determining the stoicheiometric proton dissociation constants of complexes by potentiometric titrations using a glass electrode. *Anal. Chim. Acta* **1967,** *38*, 475-488.
114. Drexler, E. J.; Field, K. W., An NMR study of Keto-Enol tautomerism in β-Dicarbonyl compounds. *J. Chem. Educ.* **1976,** *53* (6), 392-393.
115. Gentile, P. S.; Cefola, M.; Celiano, A. V., Coördination compounds. V. Determination of the dissociation constants of acetylacetone in mixed solvents. *J. Phys. Chem.* **1963,** *67* (5), 1083-1086.

S10. Routines for automated processing of NMR datasets

S10.1. Processing using Mnova 14.3.1

Run this macro script in Bruker Topspin using edmac command and open 2rr file from procno folder into Mnova:

```
# Sets up a CSI dataset for opening and processing in Mnova
# Sets SI to 32 and 32768, LB 3, phase sensitive in both dimensions (adjust if not appropriate)
#For 64 point datasets, change 1 SI to 64 and PHC1 to 11520 (180*SI)
#The script works on Bruker Topspin 3.6.2 but has not been tested on other versions
#Matthew Wallace, 1/2023
#University of East Anglia, matthew.wallace@uea.ac.uk
1 SI 32
2 SI 32768
2 LB 3
2 WDW EM
1 WDW SINE
2 PHC1 0
2 PHC0 0
2 PH_mod pk
1 PH_mod pk
#1 PHC1 should be 180*number of gradient points acquired
1 PHC1 5760
XFB
```

Run processing template as below to phase, baseline correct and reference spectra of CSI dataset. A cut region of 4.3-5.5 ppm was used for experiments performed in 50% DMSO/H_2O and 30% CD_3CN/H_2O to remove the residual water signal, except for analysis of quinine where this region was required, and no cut was applied (Figure S23). Region-specific baseline correction (-2 to 12 ppm in 50% 1-propanol/H_2O) was not applied in 50% DMSO/H_2O or 30% CD_3CN/H_2O.

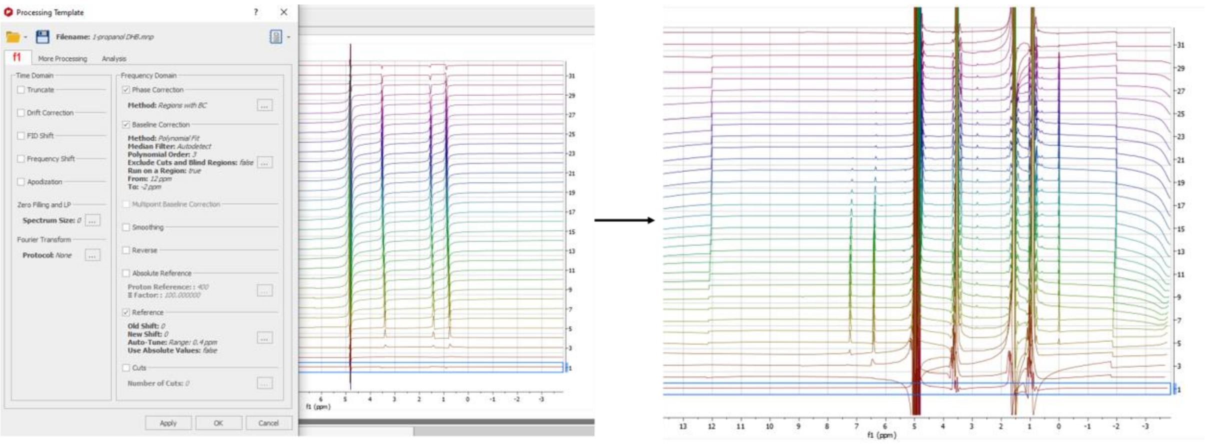

On our probe, rows 1-6 and 28-32 are slightly distorted as they arise from outside of the active region of the NMR coil. Nevertheless, the window of the CSI experiment (cnst0, Section S11) must be set to this to size (32 mm) to avoid folding artefacts from the strong signal of 1-propanol. These rows can be deleted using the stacked items table.

The chemical shift of DSS and other indicators, except those presenting doublet or quartet resonances, is extracted using the Max. Peak. Pos. function of the Data Analysis module. The chemical shift of the doublet of 2,6-dihydroxybenzoic acid is extracted by performing a clean line fitting on the region and running the script DoubletChemShift (below) to produce a .txt file of the chemical shift, running from the first row to the last. This procedure was also used for doublet resonances of pipecolic acid (Figure S25), 4CN (Figure S19) and quinine (Figure S23).

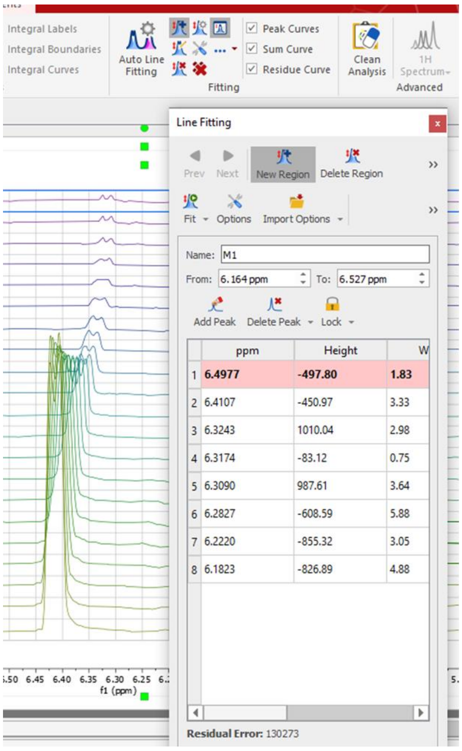

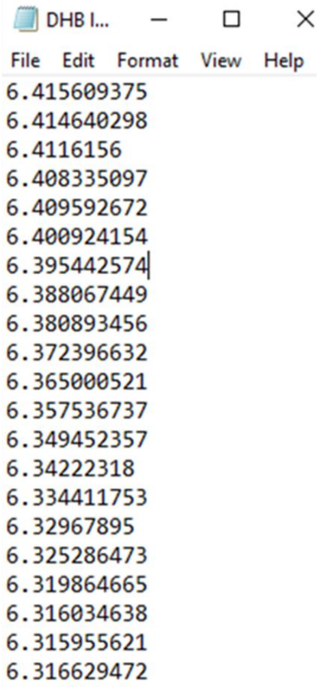

```
/*********************************************************************************************
For finding central chemical shift of multiplet (doublet or quartet) in stacked plot from Chemical Shift Imaging (CSI) dataset
Save this script in Mnova as DoubletChemShift, and Run this script
Define line fitting area with new fit region (clear all previous line fitting regions)
Click fit to fit all spectra in the stacked CSI dataset
Run this script

Script will find the most intense peak within the defined region of a spectrum
Will then find the most upfield and downfield peaks in the region with intensities within sens of biggest peak
Doublet or quartet chemical shift can then be copied and pasted into Excel

Matthew Wallace, University of East Anglia, 01/2023 (matthew.wallace@uea.ac.uk)

Based on Mnova script exportFitRegions (Copyright (C) 2014 Mestrelab Research S.L. All rights reserved, part of the Mnova scripting toolkit).
(Authorized users of Mnova Software may use this file freely, but this file is provided AS IS)
with NO WARRANTY OF ANY KIND, INCLUDING THE WARRANTY OF DESIGN, MERCHANTABILITY AND FITNESS
FOR A PARTICULAR PURPOSE.
*********************************************************************************************/

/*globals settings, Dir, FileDialog, File, TextStream, Application, NMRSpectrum, print, Peak, MnUi*/
/*jslint plusplus: true, indent: 4*/

function DoubletChemShift() {
"use strict";

function fitRegionToStream(aFitRegion, aFileStream, aNMRPeaks) {
var p, peak, tst, big, sens, tstppm, bigppm, smlppm, dbppm
sens=0.3;
tst=0;
big=0;
tstppm=0;
bigppm=-100;
fitPeaksIds = aFitRegion.peaks;
/*Find most intense peak in fitted region*/
for (p = 0; p < fitPeaksIds.length; p++) {
peak = new Peak(aNMRPeaks.byId(fitPeaksIds[p]));
tst=peak.intensity;
if(tst>big)
{
big=peak.intensity;
bigppm=peak.delta(1);
}
}
/*Find peak with highest chemical shift with intensity within sens of biggest peak*/
for (p = 0; p < fitPeaksIds.length; p++) {
peak = new Peak(aNMRPeaks.byId(fitPeaksIds[p]));
tst=peak.intensity;
tstppm=peak.delta(1);
if(tst>sens*big)
{
if(tstppm>bigppm)
{
bigppm=peak.delta(1);
}
}
}
smlppm=bigppm;
/*Find peak with most upfield chemical shift with intensity within sens of biggest peak*/
for (p = 0; p < fitPeaksIds.length; p++) {
peak = new Peak(aNMRPeaks.byId(fitPeaksIds[p]));
tst=peak.intensity;
tstppm=peak.delta(1);
if(tst>sens*big)
{
if(tstppm<smlppm)
{
smlppm=peak.delta(1);
}
}
}
dbppm=(bigppm+smlppm)/2;
aFileStream.write(dbppm, "\n");
}

var fout, sout, spc, peakList, fitRegions, fr, oldCurSpecIndex, i,
dirSettingsKey = "DoubletChemShift/LastDir",
saveDir = settings.value(dirSettingsKey, Dir.home()),
dw = Application.mainWindow.activeDocument,
spectra = dw.itemCount("NMR Spectrum"),
specIndex = 0,
fileName = FileDialog.getSaveFileName("ASCII Files (*.txt)", "", saveDir);

if (!fileName.length) {
return;
}

fout = new File(fileName);
settings.setValue(dirSettingsKey, fout.absDirPath);
if (!fout.open(File.WriteOnly)) {
throw "Impossible to open file";
}
sout = new TextStream(fout);
sout.precision = 10;

while (specIndex < spectra) {
spc = new NMRSpectrum(dw.item(specIndex, "NMR Spectrum"));
specIndex++;
if (!spc.isValid()) {
throw "Invalid Spectrum";
}
oldCurSpecIndex = spc.curSpecIndex;
for (i = 0; i < spc.specCount; i++) {
spc.curSpecIndex = i;
peakList = spc.peaks();
fitRegions = spc.fitRegions();
print(fitRegions);
for (fr = 0; fr < fitRegions.length; fr++) {
fitRegionToStream(fitRegions[fr], sout, peakList);
}
}
spc.curSpecIndex = oldCurSpecIndex;
}
fout.close();
}

if (this.MnUi && MnUi.scripts_nmr) {
MnUi.scripts_nmr.scripts_nmr_ExportASCIIFitRegions = DoubletChemShift;
}
```

To extract the integrals of DSS and 2,6-dihydroxybenzoic acid, the doublet and triplet of 2,6-dihydroxybenzoic acid can be placed in the zoom region and a baseline correction applied to this region (B). The integral of the doublet can then be extracted using the Integrals function of the Data Analysis tool. This process can be repeated for DSS and the integral added to the same table.

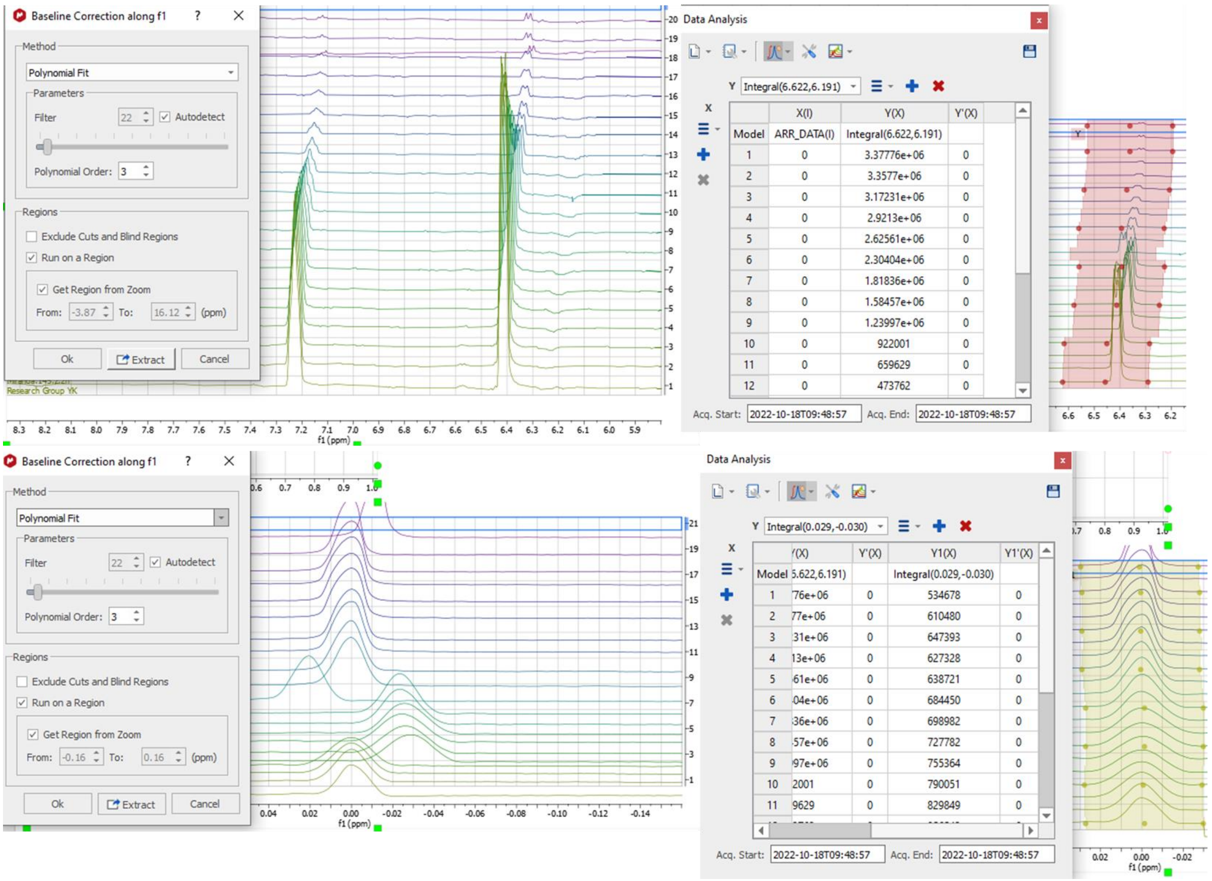

Paste the chemical shift of the doublet resonance of 2,6-dihydroxybenzoic acid and the Data Analysis table from Mnova containing the integrals into the spreadsheet. Fit the data using Solver to obtain p$K_{a,0}$, δ_H and δ_L of 2,6-dihydroxybenzoic acid. The same procedure is followed to extract p$K_{a,0}$ of 1,2,4-triazole.

S10.2 Data acquisition and processing scripts (Bruker)

S10.2.1 Script to find water suppression frequency when running under IconNMR (Bruker)

```
/* AU script for finding water suppression frequency*/
/*and running CSI experiment through IconNMR*/
/*Based on standard Bruker script, au_watersc*/
/*create a 1 scan proton parameter set (low rg) to find o1p of water, called H2O_SS or similar*/
/*Remember to change peak picking regions in this PAR set to cover the expected range for water signal*/
/*create a CSI parameter set called 1hcsi (or equivalent), this runs the csi experiment with water suppression*/
/*set parameter AUNM in this par set to the name of this script*/
/*Then set this PAR set in Icon config and run through Icon*/
/*The script works on Bruker Topspin 3.6.2 but has not been tested on other versions*/
/*This AU is not fully tested and comes without warranty.*/
/*Matthew Wallace, 9/2022*/
/*University of East Anglia, matthew.wallace@uea.ac.uk*/
 float peakFreqHz, peakFreqPPM, peakIntensity, maxpsh, maxpsp, maxips, rd;
    char path[PATH_MAX];
double sf, sfo1, sppm;
int noofscans, pscal_save, i, numPeaks;
GETCURDATA
/*Can set number of scans, sw and d1 in ICON. All other parameters will be overwritten at end*/
FETCHPAR("NS",&noofscans)
FETCHPAR("d1",&rd)
FETCHPAR("sw",&sppm)
RPAR("H2O_SS","all")
ZG
ERRORABORT
EF
ERRORABORT
APK
FETCHPAR("PSCAL",&pscal_save)
STOREPAR("PSCAL",0)
PP
ERRORABORT
  strcpy(path, PROCPATH(0));
    numPeaks = readPeakList(path);

    maxips=0.0;
    maxpsh=0.0;
    for (i=0; i<numPeaks; i++)
    {
                peakIntensity = getPeakIntensity(i);
                peakFreqHz = getPeakFreqHz(i);
                peakFreqPPM = getPeakFreqPPM(i);
                if (peakIntensity > maxips)
                {
                    maxips = peakIntensity;
                    maxpsh = peakFreqHz;
                    maxpsp = peakFreqPPM;
                }
    }
    freePeakList();

    FETCHPAR("SF",&sf);
    sfo1 = sf + maxpsh * 1.0e-6;
    STOREPAR("SFO1",sfo1);
    SETCURDATA
/*Having found water suppression frequency, read in d1, ns and sw that were set in Icon*/
RPAR("1hcsi","all")
STOREPAR("SFO1",sfo1)
STOREPAR("NS",noofscans)
STOREPAR("sw",sppm)
STOREPAR("d1",rd)
ZG
QUIT
```

S10.2.2 Script for performing receiver gain adjustment for CSI experiments under IconNMR (Bruker)

```
/*Script to perform receiver gain adjustment and run CSI dataset/*
/*No water suppression (as used for 50% 1-propanol/H2O)*/
/*Script sets gpz6 to zero (strongest signal) before performing RGA*/
/*Save this AU, compile and set AUNM to this script*/
/*This AU is not fully tested and comes without warranty.*/
/*The script works on Bruker Topspin 3.6.2 but has not been tested on other versions*/
/*Use kill command if all goes wrong*/
/*Matthew Wallace, 9/2022*/
/*University of East Anglia, matthew.wallace@uea.ac.uk*/
float gpow;
GETCURDATA
FETCHPAR("gpz6",&gpow)
STOREPAR("gpz6",0.0)
RGA
STOREPAR("gpz6",gpow)
ZG
QUIT
```

S10.2.3 Script to phase and baseline correct CSI datasets

```
/*To produce phase corrected chemical shift image from gradient encoded data*/
/*Set 1st order phase correction for f1 dimension to 180*Td1 (11520 when 64 points in image), 0th order to 0*/
/*PH_mod should be set to PK in both dimensions*/
/*XFB to produce image*/
/*This is done automatically by macro script in Section S10.1*/
/*With the 2D dataset selected, Run this Au*/
/*AU extracts each row in turn to a procno and automatically phase and baseline corrects/*
/*The script can focus on a particular region using apkf and absf if requested*/
/*Reversal of F1 axis may be necessary, depending on NMR probe*/
/*This AU is not fully tested and comes without warranty.*/
/*The script works on Bruker Topspin 3.6.2 but has not been tested on other versions*/
/*Use kill command if all goes wrong*/
/*Matthew Wallace, 9/2022*/
/*University of East Anglia, matthew.wallace@uea.ac.uk*/
char disk1[32], user1[32], location[128], phtyp[8];
float abf1=8;
float abf2=6;
int phpno=1;
int w=1;
int np=64;
int pno=5;
GETCURDATA
int steno=expno;
strcpy(location,disk);
strcpy(phtyp,"k");
GETSTRING("Enter location of dataset",location)
phpno=procno;
GETINT("Enter experiment number to process",steno)
GETINT("Enter procno containing XFB processed 2D data :",phpno)
pno=phpno+5;
GETINT("Enter procno to write rows to phase and baseline correct (empty):",pno)
REXPNO(steno)
RPROCNO(phpno)
SETCURDATA
FETCHPAR1("SI",&np)
GETINT("Enter number of points in image (autodetects) :",np)
GETSTRING("APKS (s) or APK (k) or apkf (f) auto phase correction?",phtyp)
if(strcmp(phtyp,"f")==0) {
GETFLOAT("Enter right limit for apkf and absf:",abf2)
GETFLOAT("Enter left limit for apkf and absf:",abf1) }
w=1;
TIMES(np)
        {
RPROCNO(phpno)
SETCURDATA
RSR(w,pno)
RPROCNO(pno)
SETCURDATA
if(strcmp(phtyp,"s")==0)
        {
APKS
ABS
}
if(strcmp(phtyp,"k")==0)
        {
APK
ABS
}
if(strcmp(phtyp,"f")==0)
        {
STOREPAR("absf1",abf1)
STOREPAR("absf2",abf2)
APKF
ABSF
}
WSR(w,phpno,steno,name,user,location)
w++;
}
END
QUIT
```

S10.2.4 Script to extract peak positions from phase and baseline corrected CSI datasets (Bruker)

```
/*Bruker AU script for extracting peak positions from a CSI dataset*/
/*CSI dataset should have been fully processed in phase-sensitive mode*/
/*Rough chemical shift referencing in F2 also helps*/
/*The script works on Bruker Topspin 3.6.2 but has not been tested on other versions*/
/*The script extracts each row in turn into the empty procno requested (will overwrite existing contents!!!)*/
/*Will write peak positions of 26-DHB, triazole, glycolate, acetate and DSS to separate .txt files/ppm in procno directory of CSI dataset*/
/*Adjust right and left hand peak-picking limits for each compounds as appropriate*/
/*These numbers go from first to final row of the dataset*/
/*Numbers can be copied and pasted into spreadsheet*/
/*Peak picking routine will find the centre of a multiplet (doublet, quartet, or singlet with bad shim)*/
/*Will treat two peaks as outer edges of a multiplet if their intensity is within ppsens of the biggest peak found in specified range*/
/*Adjust peak picking ranges as appropriate to avoid overlap*/
/*Verify that doublet of 2,6-DHB is being picked correctly and adjust ppsens as appropriate*/
/*Default values here apply to DMSO as chemical shift reference*/
/*This AU is not fully tested and comes without warranty.*/
/*Use kill command if all goes wrong*/
/*Matthew Wallace, 9/2022*/
/*University of East Anglia, matthew.wallace@uea.ac.uk*/
FILE *ftriz,*fref,*fac,*fglyc,*fdhb;
```

```
float min=0;
double f2pref=-0.4;
double f1pref=0.4;
double f2ptriz=7.8;
double f1ptriz=9.5;
double f2pac=1.75;
double f1pac=2.1;
double f2pdhb=6.0;
double f1pdhb=6.6;
double f2pglyc=3.7;
double f1pglyc=4.2;
float ppsens=0.8;
double pc=0.1;
int steno=15;
int eno;
int ne=5;
int m=-1;
int rn=1;
double peakFreqHz, peakFreqPPM, peakIntensity, maxpsh, maxpsp, maxips, sf,sfo1,so1p;
double mintpp,minpsp,peakppmneg,cent,ppmdif,maxpspneg;
int i, numPeaks;
int np=64;
int row=1;
int v=1;
int wrpno=5;
int phpno=1;
GETCURDATA
steno=expno;
phpno=procno;
GETINT("Enter experiment number of CSI dataset",steno)
GETINT("Enter procno of CSI dataset",phpno)
REXPNO(steno)
RPROCNO(phpno)
SETCURDATA
FETCHPAR1("td",&np)
GETINT("Enter number of gradient values",np)
GETINT("Enter procno to extract rows into for peak picking (blank)",wrpno)
GETDOUBLE("DSS (reference) right peak picking/ppm",f2pref)
GETDOUBLE("DSS (reference) left peak picking/ppm",f1pref)
GETDOUBLE("2,6-DHB right peak picking/ppm",f2pdhb)
GETDOUBLE("2,6-DHB left peak picking/ppm",f1pdhb)
GETDOUBLE("Triazole right peak picking/ppm",f2ptriz)
GETDOUBLE("Triazole left peak picking/ppm",f1ptriz)
GETDOUBLE("Glycolate right peak picking/ppm",f2pglyc)
GETDOUBLE("Glycolate left peak picking/ppm",f1pglyc)
GETDOUBLE("Acetate right peak picking/ppm",f2pac)
GETDOUBLE("Acetate left peak picking/ppm",f1pac)
GETFLOAT("Enter peak picking sensitivity factor",pc)
GETDOUBLE("Enter satellite sensitivity factor",ppsens)
STOREPAR("mi",min)
STOREPAR("pc",pc)
/*Global scaling*/
STOREPAR("pscal",0)
/*Creates text files to hold peak positions*/
if ((fref = fopen(PROCPATH("DSS.txt"),"wt")) == 0)
 STOPMSG("Cannot create file")
 if ((ftriz = fopen(PROCPATH("Triazole.txt"),"wt")) == 0)
 STOPMSG("Cannot create file")
 if ((fac = fopen(PROCPATH("Acetate.txt"),"wt")) == 0)
 STOPMSG("Cannot create file")
 if ((fdhb = fopen(PROCPATH("26-DHB.txt"),"wt")) == 0)
 STOPMSG("Cannot create file")
 if ((fglyc = fopen(PROCPATH("Glycolate.txt"),"wt")) == 0)
 STOPMSG("Cannot create file")
 /*No go through each row in turn and extract into requested procno, find peak positions*/
TIMES(np)
                    {
RSR(v,wrpno)
RPROCNO(wrpno)
SETCURDATA
/*Extract chemcial shift of reference peak first*/
STOREPAR("f2p",f2pref)
STOREPAR("f1p",f1pref)
PP
numPeaks = readPeakList(PROCPATH(0));
maxips=0.0;
 maxpsh=0.0;
 for (i=0; i<numPeaks; i++)
{
peakIntensity = getPeakIntensity(i);
peakFreqHz = getPeakFreqHz(i);
peakFreqPPM = getPeakFreqPPM(i);
if (peakIntensity > maxips)
                    {
 maxips = peakIntensity;
 maxpsh = peakFreqHz;
 maxpsp = peakFreqPPM;
 }
}
 /*Pick most downfield side of multiplet*/
 mintpp=maxips*ppsens;
maxpsp=0.0;
 for (i=0; i<numPeaks; i++)
{
 peakIntensity = getPeakIntensity(i);
 if(peakIntensity>mintpp)
{
peakFreqPPM = getPeakFreqPPM(i);
peakFreqHz = getPeakFreqHz(i);
```

```
if (peakFreqHz >= maxpsh)
                    {
 maxpsp = peakFreqPPM;
 maxpsh = peakFreqHz;
 }
}
}
/*Flips negative to choose most upfield peak of multiplet*/
for (i=0; i<numPeaks; i++)
{
 peakIntensity = getPeakIntensity(i);
 if(peakIntensity>mintpp)
{
peakFreqPPM = getPeakFreqPPM(i);
peakppmneg=peakFreqPPM*m;
maxpspneg=maxpsp*m;
if (peakppmneg >= maxpspneg)
                    {
 minpsp = peakFreqPPM;
 }
}
}
 freePeakList();
/*writes chemical shift into text document*/
ppmdif=maxpsp-minpsp;
cent=minpsp+ppmdif*0.5;
 fprintf(fref,"%f \n",cent);
/*Reads in for triazole*/
STOREPAR("f2p",f2ptriz)
STOREPAR("f1p",f1ptriz)
STOREPAR("mi",min)
PP
numPeaks = readPeakList(PROCPATH(0));
maxips=0.0;
 maxpsh=0.0;
 for (i=0; i<numPeaks; i++)
{
peakIntensity = getPeakIntensity(i);
peakFreqHz = getPeakFreqHz(i);
peakFreqPPM = getPeakFreqPPM(i);
if (peakIntensity > maxips)
                    {
 maxips = peakIntensity;
 maxpsh = peakFreqHz;
 maxpsp = peakFreqPPM;
 }
}
/*Pick most downfield side of multiplet*/
 mintpp=maxips*ppsens;
maxpsp=0.0;
 for (i=0; i<numPeaks; i++)

{
 peakIntensity = getPeakIntensity(i);
 if(peakIntensity>mintpp)

{
peakFreqPPM = getPeakFreqPPM(i);
peakFreqHz = getPeakFreqHz(i);
if (peakFreqHz >= maxpsh)
                    {
 maxpsp = peakFreqPPM;
 maxpsh = peakFreqHz;
 }
}
}
/*Flips negative to choose most upfield peak of multiplet*/
for (i=0; i<numPeaks; i++)

{
 peakIntensity = getPeakIntensity(i);
 if(peakIntensity>mintpp)
{
peakFreqPPM = getPeakFreqPPM(i);
peakppmneg=peakFreqPPM*m;
maxpspneg=maxpsp*m;
if (peakppmneg >= maxpspneg)
{
 minpsp = peakFreqPPM;
 }
}
}
 freePeakList();
/*writes chemical shift into text document*/
ppmdif=maxpsp-minpsp;
cent=minpsp+ppmdif*0.5;
 fprintf(ftriz,"%f \n",cent);
/*Reads in for Acetate*/
STOREPAR("f2p",f2pac)
STOREPAR("f1p",f1pac)
PP
numPeaks = readPeakList(PROCPATH(0));
maxips=0.0;
 maxpsh=0.0;
 for (i=0; i<numPeaks; i++)

{
peakIntensity = getPeakIntensity(i);
peakFreqHz = getPeakFreqHz(i);
```

```
peakFreqPPM = getPeakFreqPPM(i);
if (peakIntensity > maxips) {
 maxips = peakIntensity;
 maxpsh = peakFreqHz;
 maxpsp = peakFreqPPM; }

}
/*Pick most downfield side of multiplet*/
 mintpp=maxips*ppsens;
maxpsp=0.0;
 for (i=0; i<numPeaks; i++)

{
 peakIntensity = getPeakIntensity(i);
 if(peakIntensity>mintpp)

{
peakFreqPPM = getPeakFreqPPM(i);
peakFreqHz = getPeakFreqHz(i);
if (peakFreqHz >= maxpsh) {
 maxpsp = peakFreqPPM;
 maxpsh = peakFreqHz; }

}
}
/*Flips negative to choose most upfield peak of multiplet*/
for (i=0; i<numPeaks; i++)

{
 peakIntensity = getPeakIntensity(i);
 if(peakIntensity>mintpp)

{
peakFreqPPM = getPeakFreqPPM(i);
peakppmneg=peakFreqPPM*m;
maxpspneg=maxpsp*m;
if (peakppmneg >= maxpspneg) {
 minpsp = peakFreqPPM; }

}
}
 freePeakList();
/*writes chemical shift into text document*/
ppmdif=maxpsp-minpsp;
cent=minpsp+ppmdif*0.5;
 fprintf(fac,"%f \n",cent);
/*Reads in for glycolate*/
STOREPAR("f2p",f2pglyc)
STOREPAR("f1p",f1pglyc)
PP
numPeaks = readPeakList(PROCPATH(0));
maxips=0.0;
 maxpsh=0.0;
 for (i=0; i<numPeaks; i++)
{
peakIntensity = getPeakIntensity(i);
peakFreqHz = getPeakFreqHz(i);
peakFreqPPM = getPeakFreqPPM(i);
if (peakIntensity > maxips)
                    {
 maxips = peakIntensity;
 maxpsh = peakFreqHz;
 maxpsp = peakFreqPPM;
  }
}
/*Pick most downfield side of multiplet*/
 mintpp=maxips*ppsens;
maxpsp=0.0;
 for (i=0; i<numPeaks; i++)

{
 peakIntensity = getPeakIntensity(i);
 if(peakIntensity>mintpp)
{
peakFreqPPM = getPeakFreqPPM(i);
peakFreqHz = getPeakFreqHz(i);
if (peakFreqHz >= maxpsh) {
 maxpsp = peakFreqPPM;
 maxpsh = peakFreqHz; }
}
}
/*Flips negative to choose most upfield peak of multiplet*/
for (i=0; i<numPeaks; i++)
 {
 peakIntensity = getPeakIntensity(i);
 if(peakIntensity>mintpp)
 {
peakFreqPPM = getPeakFreqPPM(i);
peakppmneg=peakFreqPPM*m;
maxpspneg=maxpsp*m;
if (peakppmneg >= maxpspneg)
{
 minpsp = peakFreqPPM;
}
 }
}
 freePeakList();
/*writes chemical shift into text document*/
ppmdif=maxpsp-minpsp;
```

```
cent=minpsp+ppmdif*0.5;
 fprintf(fglyc,"%f \n",cent);
/*Reads in for 26-DHB*/
STOREPAR("f2p",f2pdhb)
STOREPAR("f1p",f1pdhb)
PP
numPeaks = readPeakList(PROCPATH(0));
maxips=0.0;
 maxpsh=0.0;
 for (i=0; i<numPeaks; i++)
 {
peakIntensity = getPeakIntensity(i);
peakFreqHz = getPeakFreqHz(i);
peakFreqPPM = getPeakFreqPPM(i);
if (peakIntensity > maxips)
{
 maxips = peakIntensity;
 maxpsh = peakFreqHz;
 maxpsp = peakFreqPPM;
}
 }
/*Pick most downfield side of multiplet*/
 mintpp=maxips*ppsens;
maxpsp=0.0;
 for (i=0; i<numPeaks; i++)
 {
 peakIntensity = getPeakIntensity(i);
 if(peakIntensity>mintpp)
 {
peakFreqPPM = getPeakFreqPPM(i);
peakFreqHz = getPeakFreqHz(i);
if (peakFreqHz >= maxpsh)
{
 maxpsp = peakFreqPPM;
 maxpsh = peakFreqHz;
}
 }
}
/*Flips negative to choose most upfield peak of multiplet*/
for (i=0; i<numPeaks; i++)
 {
 peakIntensity = getPeakIntensity(i);
 if(peakIntensity>mintpp)
 {
peakFreqPPM = getPeakFreqPPM(i);
peakppmneg=peakFreqPPM*m;
maxpspneg=maxpsp*m;
if (peakppmneg >= maxpspneg)
{
 minpsp = peakFreqPPM;
}
 }
}
 freePeakList();
/*writes chemical shift into text document*/
ppmdif=maxpsp-minpsp;
cent=minpsp+ppmdif*0.5;
 fprintf(fdhb,"%f \n",cent);
v++;
RPROCNO(phpno)
SETCURDATA
}
END
fclose(fref);
fclose(fac);
fclose(ftriz);
fclose(fglyc);
fclose(fdhb);
QUIT
```

S10.2.5 Script to extract chemical shifts and integrals of 2,6-DHB and DSS from CSI datasets (Bruker)

```
/*Script for integrating 3,5-resonance of 2,6-DHB and methyl resonance of DSS*/
/*Choose a low row of the CSI dataset with a high concentration of 2,6-DHB*/
/*Reference to DSS (0 ppm), then create an integral region spanning either side (e.g. 0.2 to -0.2 ppm)*/
/*Judge width of 3,5-resonance of 2,6-DHB then create integral region centred at 6.3 ppm and spanning either side*/
/*wide enough so that DHB resonance is fully covered when doublet of 2,6-DHB is centred at 6.3 ppm (e.g. 6.6 to 6.0 ppm*/
/*Integral file should only conatin these two integral regions*/
/*Save this integral file using wmisc command as dhbdss, or else change strcpy(text,"dhbdss") below to edit default name that the script uses*/
/*The script extracts each row in turn into the empty procno requested (will overwrite existing contents!!!)*/
/*Working in this procno, the spectrum is referenced to DSS and the integral read in and absolute value saved in text file*/
/*The same row is written into another procno (+100) and referenced to 2,6-DHB doublet at 6.3 ppm*/
/*The integral of 3,5-position of 2,6-DHB is then read and saved in a text file*/
/*All text files are stored in the procno directory of the CSI dataset*/
/*Change right and left peak picking limits if any risk of a non-reference peak being included in the referencing procedure*/
/*Verify that doublet of 2,6-DHB is being picked correctly into text files and adjust ppsens as appropriate*/
/*The script works on Bruker Topspin 3.6.2 but has not been tested on other versions*/
/*This AU is not fully tested and comes without warranty.*/
/*Use kill command if all goes wrong*/
/*Matthew Wallace*/
/*1/2023*/
/*University of East Anglia, matthew.wallace@uea.ac.uk*/
FILE    *fpnt,*fref,*fac,*fdhbppm,*fdssppm;
char savans[8],dummystr[256],intdir[256],location[128],ordans[8];
float min=0;
float lb=1;
```

```
double f2pdss=-0.5;
double f1pdss=0.5;
double ref=0;
double f2pdhb=5.7;
double f1pdhb=6.8;
double dhbref=6.3;
float ppsens=0.8;
double pc=0.1;
int m=-1;
int rn=1;
int steno;
double peakFreqHz, peakFreqPPM, peakIntensity, maxpsh, maxpsp, maxips, sf,sfn,sfo1,intgr;
double intnum,ppmdn,ppmup,intgrso1p,mintpp,minpsp,peakppmneg,cent,ppmdif,maxpspneg;
int i, numPeaks;
int ne=4;
int row=1;
int v=1;
int linenum=1;
int phpno=1;
int np=32;
int wrpno=5;
int wrpnod=6;
strcpy(location,disk);
strcpy(savans,"y");
strcpy(text,"dhbdss");
strcpy(ordans,"u");
GETCURDATA
steno=expno;
phpno=procno;
wrpno=phpno+605;
FETCHPAR1("td",&np)
wrpnod=wrpno+100;
GETINT("Enter experiment number to process",steno)
GETINT("Enter number of spectra in CSI image",np)
GETINT("Enter procno of 2D CSI dataset",phpno)
GETINT("Enter first procno for integration of reference (blank)",wrpno)
GETINT("Enter first procno for integration of DHB (blank)",wrpnod)
GETDOUBLE("DSS right peak picking/ppm",f2pdss)
GETDOUBLE("DSS left peak picking/ppm",f1pdss)
GETDOUBLE("2,6-DHB right peak picking/ppm",f2pdhb)
GETDOUBLE("2,6-DHB left peak picking/ppm",f1pdhb)
GETDOUBLE("Enter DSS reference shift/ppm",ref)
GETFLOAT("Enter peak picking senistitivty factor",pc)
GETDOUBLE("Enter satelite sensitivity factor for peak picking",ppsens)
GETSTRING("Which intrng file must be used?", text)
/*Create text files to hold integral data*/
if ((fref = fopen(PROCPATH("DSS integral.txt"),"wt")) == 0)
    STOPMSG("Cannot create file")
if ((fac = fopen(PROCPATH("2,6-DHB integral.txt"),"wt")) == 0)
    STOPMSG("Cannot create file")
if ((fdhbppm = fopen(PROCPATH("2,6-DHB chemical shift ppm.txt"),"wt")) == 0)
    STOPMSG("Cannot create file")
if ((fdssppm = fopen(PROCPATH("DSS chemical shift ppm.txt"),"wt")) == 0)
    STOPMSG("Cannot create file")
REXPNO(steno)
SETCURDATA
TIMES(np)
{
RPROCNO(phpno)
SETCURDATA
RSR(v,wrpno)
RPROCNO(wrpno)
SETCURDATA
STOREPAR("pscal",0)
STOREPAR("mi",min)
STOREPAR("pc",pc)
STOREPAR("CURPRIN","Integrals.txt")
sprintf(intdir,"%s/%s/%i/pdata/%i/integrals.txt",location,name,expno,procno);
/*Reference the spectrum.*/
/*If the reference peak is split, the program will reference based on the average shift*/
/*of the two peaks in the requested peak pikcing range which are within ppsens of the largest peak found*/
STOREPAR("f2p",f2pdss)
STOREPAR("f1p",f1pdss)
PP
 numPeaks = readPeakList(PROCPATH(0));
maxips=0.0;
    maxpsh=0.0;
    for (i=0; i<numPeaks; i++)
    {
                  peakIntensity = getPeakIntensity(i);
                  peakFreqHz = getPeakFreqHz(i);
                  peakFreqPPM = getPeakFreqPPM(i);
                  if (peakIntensity > maxips)
                  {
                      maxips = peakIntensity;
                      maxpsh = peakFreqHz;
                      maxpsp = peakFreqPPM;
                  }
    }
    /*Pick most downfield side of multiplet*/
    mintpp=maxips*ppsens;
maxpsp=0.0;
    for (i=0; i<numPeaks; i++)
    {
                                    peakIntensity = getPeakIntensity(i);
                                    if(peakIntensity>mintpp)
                                    {
                  peakFreqPPM = getPeakFreqPPM(i);
                  peakFreqHz = getPeakFreqHz(i);
```

```
                    if (peakFreqHz >= maxpsh)
                    {
                        maxpsp = peakFreqPPM;
                        maxpsh = peakFreqHz;
                    }
    }
                    }
/*Flips negative to choose most upfield peak of multiplet*/
for (i=0; i<numPeaks; i++)
    {
                                        peakIntensity = getPeakIntensity(i);
                                        if(peakIntensity>mintpp)
                                        {
                    peakFreqPPM = getPeakFreqPPM(i);
peakppmneg=peakFreqPPM*m;
maxpspneg=maxpsp*m;
                    if (peakppmneg >= maxpspneg)
                    {
                        minpsp = peakFreqPPM;
                    }
    }
                    }
    freePeakList();
/*References spectrum*/
ppmdif=maxpsp-minpsp;
cent=minpsp+ppmdif*0.5;
FETCHPAR("sf",&sf)
    sfn=sf+(cent-ref)*sf/(1e6);
    fprintf(fdssppm,"%f \n",cent);
    STOREPAR("sf",sfn)
/*Now do absf on dss*/
STOREPAR("absf2",f2pdss)
STOREPAR("absf1",f1pdss)
APKF
ABSF
/*Read in integral file and get integral of dss*/
                    RMISC("intrng", text)
LI
fpnt=fopen(intdir, "r");
fgets(dummystr, sizeof(dummystr), fpnt);
while (fgets(dummystr, sizeof(dummystr), fpnt) != NULL)
{
                    /*Need to selectively elimate rows, then scan for numbers*/
if(linenum>=5)
{
                    (void) sscanf(dummystr,"%lf %lf %lf %lf",
                                                            &intnum,&ppmdn,&ppmup,&intgr);
                                                            /*dss is first, then acetate*/
                                                                                if(linenum==5)
                                                            {
                                                                                if(strcmp(ordans,"d")==0)
                                                                                {
                                                            fprintf(fref,"%f\n",intgr);
                                                                                }
                                                            }
                                                                                if(linenum==6)
                                                            {
                                                                                                    if(strcmp(ordans,"u")==0)
                                                                                {
                                                            fprintf(fref,"%f\n",intgr);
                                                                                }
                                                            }
                                                            intnum=0;
                                                            ppmdn=0;
                                                            ppmup=0;
                                                            intgr=0;
                                                            linenum++;
}
                    else
{
                                                            linenum++;
}
}
linenum=1;
fclose(fpnt);
/*Now do same for DHB, starting with extracted row again*/
RPROCNO(phpno)
SETCURDATA
RSR(v,wrpnod)
RPROCNO(wrpnod)
SETCURDATA
STOREPAR("pscal",0)
STOREPAR("mi",min)
STOREPAR("pc",pc)
STOREPAR("CURPRIN","Integrals.txt")
sprintf(intdir,"%s/%s/%i/pdata/%i/integrals.txt",location,name,expno,procno);
STOREPAR("f2p",f2pdhb)
STOREPAR("f1p",f1pdhb)
PP
 numPeaks = readPeakList(PROCPATH(0));
maxips=0.0;
    maxpsh=0.0;
    for (i=0; i<numPeaks; i++)
    {
                    peakIntensity = getPeakIntensity(i);
                    peakFreqHz = getPeakFreqHz(i);
                    peakFreqPPM = getPeakFreqPPM(i);
                    if (peakIntensity > maxips)
                    {
```

```
                maxips = peakIntensity;
                maxpsh = peakFreqHz;
                maxpsp = peakFreqPPM;
            }
    }
    /*Pick most downfield side of multiplet*/
    mintpp=maxips*ppsens;
maxpsp=0.0;
    for (i=0; i<numPeaks; i++)
    {
                    peakIntensity = getPeakIntensity(i);
                    if(peakIntensity>mintpp)
                    {
            peakFreqPPM = getPeakFreqPPM(i);
            peakFreqHz = getPeakFreqHz(i);
            if (peakFreqHz >= maxpsh)
            {
                maxpsp = peakFreqPPM;
                maxpsh = peakFreqHz;
            }
    }
            }
/*Flips negative to choose most upfield peak of multiplet*/
for (i=0; i<numPeaks; i++)
    {
                    peakIntensity = getPeakIntensity(i);
                    if(peakIntensity>mintpp)
                    {
            peakFreqPPM = getPeakFreqPPM(i);
peakppmneg=peakFreqPPM*m;
maxpspneg=maxpsp*m;
            if (peakppmneg >= maxpspneg)
            {
                minpsp = peakFreqPPM;
            }
    }
            }
    freePeakList();
/*References spectrum*/
ppmdif=maxpsp-minpsp;
cent=minpsp+ppmdif*0.5;
FETCHPAR("sf",&sf)
if(f2pdhb<cent)
{
            if(f1pdhb>cent)
            {
    sfn=sf+(cent-dhbref)*sf/(1e6);
            }
}
    STOREPAR("sf",sfn)
fprintf(fdhbppm,"%f \n",cent);
    /*Now do absf on DHB*/
STOREPAR("absf2",f2pdhb)
STOREPAR("absf1",f1pdhb)
APKF
ABSF
/*Read in integral file and get integral of Dhb*/
            RMISC("intrng", text)
LI
fpnt=fopen(intdir, "r");
fgets(dummystr, sizeof(dummystr), fpnt);
while (fgets(dummystr, sizeof(dummystr), fpnt) != NULL)
{
            /*Need to selectively elimate rows, then scan for numbers*/
if(linenum>=5)
{
            (void) sscanf(dummystr,"%lf %lf %lf %lf",
                            &intnum,&ppmdn,&ppmup,&intgr);
                            /*dss is first, then acetate*/
                                        if(linenum==5)
                            {
                                        if(strcmp(ordans,"u")==0)
                                        {
                            fprintf(fac,"%f\n",intgr);
                                        }
                            }
                                        if(linenum==6)
                            {
                                                    if(strcmp(ordans,"d")==0)
                                        {
                            fprintf(fac,"%f\n",intgr);
                                        }
                            }
                            intnum=0;
                            ppmdn=0;
                            ppmup=0;
                            intgr=0;
                            linenum++;
}
            else
{
                            linenum++;
}
}
linenum=1;
v++;
wrpno++;
wrpnod++;
}
```

```
END
fclose(fpnt);
fclose(fac);
fclose(fref);
fclose(fdhbppm);
fclose(fdssppm);
QUIT
```

S11. CSI pulse sequences (Bruker)

S11.1 Spin-echo sequence (no solvent suppression)

```
;Sequence for 1H CSI using spin-echo with lock, spoil gradient after acquisition to allow short AQ
;swap UN(BLKGRAD) statements for UN(BLKGRAMP) if running without lock
;Modified from: "Probing spatial distribution of alignment by deuterium NMR imaging"
;Chem. Eur. J., 9, 2013, 7013-7019. DOI: 10.1002/chem.201300254
;2D sequence for z-imaging preserving chemical shift
;Original sequence written by Christian Merle, Martin Koos
;Modified to be on 1H with spin echo
;Set 1 SW to Z-range in mm (see cnst0) to get 1 Hz/mm scale in indirect dimension
;Make cnst0 bigger than actual sample size to avoid folding artefacts.
;Keep gpz6 at 100% and adjust cnst3 to get p30 to an acceptable length according to instrument (ca. 150-300 us)
;This pulse program is not fully tested and comes without warranty.
;Check the sequence and your parameters carefully before use.
;Matthew Wallace, 9/2022 (University of East Anglia, matthew.wallace@uea.ac.uk)
;1H-Version
;$CLASS=HighRes
;$DIM=2D
;$TYPE=
;$SUBTYPE=
;$COMMENT=
prosol relations=<triple>
#include <Avance.incl>
#include <Grad.incl>
#include <Delay.incl>
"cnst2= 0.8914027" ; integralfactor of gradient shape SMSQ10.32
"cnst4= 267.52220" ; * 10^6 /Ts = gamma1H
"p30=(td1/cnst0)*(1/(cnst1*cnst2*cnst3))*(1/cnst4)*(2*3.14159265/1000)*0.5 s"
"l1=td1-1"
lgrad r1d = l1
"acqt0=0"
"l1=td1-1"
"p2=p1*2"
"DELTA1=d6+p30+5u+d16"
 baseopt_echo
1 ze
2 30m
50u BLKGRAD
3 d1
 50u UNBLKGRAD
p19:gp3
d16
  p1 ph1
DELTA1
p2 ph2
d6
p30:gp6*r1d*cnst3
5u
d16
go=2 ph31
50u BLKGRAD
30m wr #0 if #0 zd igrad r1d
lo to 3 times l1
goto 5
; run last increment:
4 30m
50u BLKGRAD
5 d1
;spoil gradient from previous
50u UNBLKGRAD
p19:gp3
d16
p1 ph1
DELTA1
p2 ph2
d6
p30:gp6*r1d*cnst3
5u
d16
go=4 ph31
50u BLKGRAD
30m wr #0 if #0 zd
exit
ph1=0 0 2 2 1 1 3 3
ph2=1 3 1 3 0 2 0 2
ph31=0 0 2 2 1 1 3 3

;cnst0 : z-Range in cm
;cnst1 : GCC (G/mm) from Gradpar
;cnst3 : set to get GP of sufficient length
;pl1 : f1 channel - power level for pulse (default)
;p1 : f1 channel - 90 degree high power pulse
;p19: spoil pulse [600u]
;gpz6: 100% phase encoding gradient
;d16: standard eddy delay [200u]
;d1 :  relaxation delay
```

```
;d6 : pre GP delay [10u]
;ns: 2*n
;ds: 1*m
;td1: number of experiments
;FnMODE: QF
;gpz3: 50%
;use gradient files
;gpnam3: SMSQ10.100
;gpnam6: SMSQ10.32
;$Id: phaseenc,v 1.1 2011/08/10 15:12:45 ber Exp $
```

S11.2 CSI sequence with perfect-echo water suppression

```
;Modified from: "Probing spatial distribution of alignment by deuterium NMR imaging"
;Chemistry - A European Journal, Volume 19, Issue 22, 27 May 2013, Pages 7013-7019
;2D sequence for z-imaging preserving chemical shift. DOI: 10.1002/chem.201300254
;using a phase encoding gradient. Original sequence written by Christian Merle, Martin Koos
;Modified to be on 1H with perfect echo excitation sculpting for water suppression
;Water suppression component is taken from:
;zgesgppe
;avance-version (13/08/01)
;1D sequence
;water suppression using excitation sculpting with gradients
;   using perfect echo
;(R.W. Adams, C.M. Holroyd, J.A. Aguilar, M. Nilsson & G.A. Morris,
;   Chem. Commun. 49, 358-360 (2013))
;T.-L. Hwang & A.J. Shaka, J. Magn. Reson.,
;   Series A 112 275-279 (1995)
;
;This pulse program is not fully tested and comes without warranty.
;statements as detailed in the comments
;Matthew Wallace, 9/2022 (University of East Anglia, matthew.wallace@uea.ac.uk)
;
;Set 1 SW to Z-range in mm (see cnst0) to get 1 Hz/mm scale in indirect dimension
;Make cnst0 bigger than actual sample size to avoid folding artefacts.
;Keep gpz6 at 100% and adjust cnst3 to get p30 to an acceptable length according to instrument (ca. 150-300 us)
; 1H-Version
;$CLASS=HighRes
;$DIM=2D
;$TYPE=
;$SUBTYPE=
;$COMMENT=
prosol relations=<triple>
#include <Avance.incl>
#include <Grad.incl>
#include <Delay.incl>
;gradient duration equals aq in a regular 2D experiment
; AQ= TD/2SW, SW is determined by gradient strength
; SW= 2 gamma/2pi *G*zmax (all in SI), G=0.95*0.05T/mA*10A*integfactor
; p30=AQ= TD/deltaz * pi/(gamma*G), deltaz = 2*zmax
;to keep the numbers short and easy to enter the following dimensions are used:
; deltaz in cm
; gamma in Mega*1/Ts
; G in Gs/cm
;conversion of all variables combined is done by *1/100
"cnst2= 0.8914027" ; integralfactor of gradient shape smsq
"cnst4= 267.52220" ; * 10^6 /Ts = gamma1H
"p30=(td1/cnst0)*(1/(cnst1*cnst2*cnst3))*(1/cnst4)*(2*3.14159265/1000)*0.5 s"
;this function will return the gradient levels,
;using loopcounter as a workaround for brukers' functions' inabilities to deal
;with complicated arithmetics like differences.
;gradient function will never reach +1, this run will be covered separately
"l1=td1-1"
lgrad r1d = l1
;correct some phase shifts
"acqt0=0"
"DELTA1=p12+p16+d16+p2/2+de/2+p1/PI+12u"
"DELTA2=p30+d16"
"TAU=de+p1*2/PI"
"p2=p1*2"
"d12=20u"
"d4=d1-100m"
baseopt_echo

1 ze
2 30m BLKGRAD
d1
;spoil gradient from previous
3 50u UNBLKGRAD
p19:gp5
d16
;start of zggesgppe
d12 pl1:f1
 (p1 ph1)

  p16:gp3
  d16
 DELTA1
DELTA2
  (p2 ph7)
DELTA2
  DELTA1
  p16:gp3
  d16

  (p1 ph6)
```

```
  p16:gp1
  d16
  (p12:sp1 ph2:r):f1
  4u
  4u pl1:f1

  p2 ph3

  4u
  p16:gp1
  d16
  TAU
                    DELTA2
  p16:gp2
  d16
  (p12:sp1 ph4:r):f1
  4u
  4u pl1:f1

  p2 ph5

  4u
  p16:gp2
  d16
p30:gp6*cnst3*r1d
d16
go=2 ph31
30m BLKGRAD
100m wr #0 if #0 zd igrad r1d
d4
lo to 3 times l1
goto 5
; run last increment:
4 30m BLKGRAD
d1
;spoil gradient from previous
5 50u UNBLKGRAD
p19:gp5
d16
  ;start of zggesgppe
d12 pl1:f1
   (p1 ph1)

  p16:gp3
  d16
  DELTA1
DELTA2
  (p2 ph7)
DELTA2
  DELTA1
  p16:gp3
  d16

  (p1 ph6)

  p16:gp1
  d16
  (p12:sp1 ph2:r):f1
  4u
  4u pl1:f1

  p2 ph3

  4u
  p16:gp1
  d16
  TAU
                    DELTA2
  p16:gp2
  d16
  (p12:sp1 ph4:r):f1
  4u
  4u pl1:f1

  p2 ph5

  4u
  p16:gp2
  d16
p30:gp6*cnst3*r1d
d16
go=4 ph31
30m BLKGRAD
100m wr #0 if #0 zd
exit
ph1=0
ph2=0 1
ph3=2 3
ph4=0 0 1 1
ph5=2 2 3 3
ph6=1
ph7=0
ph31=0 2 2 0
;pl0 : 0W
;cnst0 : z-Range in cm
;cnst1 : GCC (G/mm) from Gradpar
;cnst3 : set max to get long GP of ca 100-200us [0.95 max]
;cnst9: Set to TD1
```

```
;pl1 : f1 channel - power level for pulse (default)
;sp1 : f1 channel - shaped pulse 180 degree
;p1 : f1 channel - 90 degree high power pulse
;p12: f1 channel - 180 degree shaped pulse (Gauss)   [4 msec]
;p16: homospoil/gradient pulse (1000 us)
;p19 : Spoil gradient pulse (1000 us)
;gpz6: 100% phase encoding gradient
;d1 :  Relaxation delay
;d12: delay for power switching                    [20 usec]
;d16: standard eddy delay (200u)
;ns: 8 * n, total number of scans: NS * TD0
;ds: 4*n [16]
;td1: number of experiments
;FnMODE: QF


;for z-only gradients:
;gpz1: 31%
;gpz2: 11%
;gpz3: 5%
;gpz5: 50%
;use gradient files:

;gpnam1: SMSQ10.100
;gpnam2: SMSQ10.100
;gpnam3: SMSQ10.100
;gpnam5: SMSQ10.100
;gpnam6: SMSQ10.32
;$Id: phaseenc,v 1.1 2011/08/10 15:12:45 ber Exp $
```
